# A Novel Mechanism for SIRT1 Activators That Does Not Rely on the Chemical Moiety Immediately C-Terminal to the Acetyl-Lysine of the Substrate

**DOI:** 10.3390/molecules27092714

**Published:** 2022-04-22

**Authors:** Nian-Da Yu, Bing Wang, Xin-Zhu Li, Hao-Zhen Han, Dongxiang Liu

**Affiliations:** 1 Center for Chemical Biology, Shanghai Institute of Materia Medica, Chinese Academy of Sciences, Shanghai 201203, China; nda_yu@163.com (N.-D.Y.); wangbing@simm.ac.cn (B.W.); hhaozhen@126.com (H.-Z.H.); 2 College of Pharmacy, University of Chinese Academy of Sciences, No. 19A Yuquan Road, Beijing 100049, China; 3 School of Chinese Materia Medica, Nanjing University of Chinese Medicine, Nanjing 210023, China; j20-lixinzhu-nj@simm.ac.cn

**Keywords:** SIRT1, activator, covalent bond, 2′-O-acetyl-ADP-ribose, deacetylase

## Abstract

SIRT1, an NAD^+^-dependent deacetylase, catalyzes the deacetylation of proteins coupled with the breakdown of NAD^+^ into nicotinamide and 2′-O-acetyl-ADP-ribose (OAADPr). Selective SIRT1 activators have potential clinical applications in atherosclerosis, acute renal injury, and Alzheimer’s disease. Here, we found that the activity of the potent SIRT1 activator CWR is independent of the acetylated substrate. It adopts a novel mechanism to promote SIRT1 activity by covalently bonding to the anomeric C1′ carbon of the ribose ring in OAADPr. In addition, CWR is highly selective for SIRT1, with no effect on SIRT2, SIRT3, SIRT5, or SIRT6. The longer distance between the anomeric C1′ carbon of the ribose ring in OAADPr and Arg274 of SIRT1 (a conserved residue among sirtuins) than that between the anomeric C1′ carbon in OAADPr and the Arg of SIRT2, SIRT3, SIRT5, and SIRT6, should be responsible for the high selectivity of CWR for SIRT1. This was confirmed by site-directed mutagenesis of SIRT3. Consistent with the in vitro assays, the activator also reduced the acetylation levels of p53 in a concentration-dependent manner via SIRT1 in cells. Our study provides a new perspective for designing SIRT1 activators that does not rely on the chemical moiety immediately C-terminal to the acetyl-lysine of the substrate.

## 1. Introduction

Unlike Zn^2+^-dependent type I, II, and IV lysine deacetylases (KDACs), class III KDACs, which are known as sirtuins, utilize nicotinamide adenine dinucleotide (NAD^+^) as a cofactor to catalyze the deacetylation of proteins. Human sirtuins have seven isoforms (SIRT1–SIRT7) with different subcellular localizations and functions. They generally catalyze the deacetylation of acetylated substrates, coupled with the breakdown of NAD^+^ into nicotinamide and 2′-O-acetyl-ADP-ribose (OAADPr). For SIRT1, more than sixty cellular substrates have been identified [1]. It plays an important role in many biological functions. For instance, it exerts anti-atherosclerotic effects by activating endothelial nitric oxide synthase (eNOS) or by reducing hepatic proprotein convertase subtilisin/kexin type 9 (Pcsk9) secretion [2,3]. The upregulation of SIRT1 expression attenuates acute kidney injuries via the inhibition of the p53 upregulated modulator of apoptosis (PUMA) induced by FOXO3a deacetylation [4]. By increasing the lysosome number and deacetylating lysosome-related proteins [5], SIRT1 also promotes β-amyloid peptide (Aβ) degradation in primary astrocytes, which is beneficial in Alzheimer’s disease. The protective role of SIRT1 in cardiovascular disease [2,3], metabolic disorders [6] neurodegenerative diseases [7], and inflammation [8,9] renders this enzyme an attractive therapeutic target.

**Figure 1:**
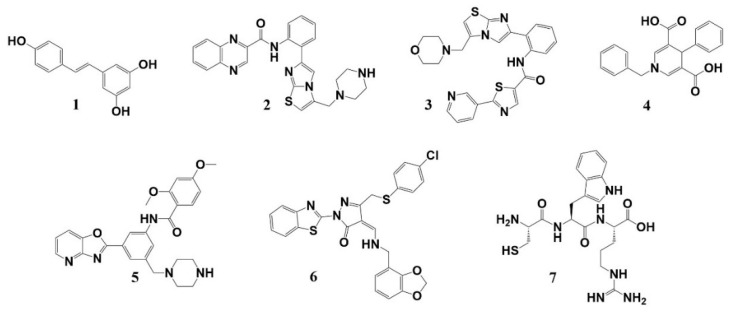
Representative SIRT1 activators. Compound **1**: resveratrol; Compound **2**: SRT1720; Compound **3**: SRT2104; Compound **4**: 1,4-dihydropyridine-based derivative; Compound **5**: Oxazolo [4,5-b] pyridines; Compound **6**: F0911-7667; Compound **7**: CWR tripeptide.

So far, a few SIRT1 activators have been discovered. Resveratrol (1), a natural compound, was the first reported activator, which could increase cell survival by stimulating the deacetylation of p53 (Figure 1) [10]. Synthetic SIRT1 activators, such as SRT1720 (2), can improve insulin sensitivity, lower plasma glucose, and increase mitochondrial capacity in diet-induced obese and leptin-deficient (Lep^ob/ob^) mice [11], but suffer from a limited target specificity [12]. SRT2104 (3), with the same core structure as SRT1720, also showed poor and variable pharmacokinetics upon oral administration [13]. As the activator of SIRT1/2/3, the activity of a 1,4-dihydropyridine-based derivative (4) on SIRT1 was confirmed in senescence assays performed on hMSCs and in mitochondrial function studies conducted with murine C2C12 myoblasts [14]. Oxazolo [4,5-b] pyridines (5) and their related heterocyclic analogs have also been described as SIRT1 activators [15]. In addition, the compound F0911-7667 (6), as a SIRT1 activator, induced autophagic cell death via the AMPK–mTOR–ULK complex in U87MG and T98G cells [16]. Furthermore, Kumar et al. identified the CWR tripeptide (7) as a SIRT1 activator, which was shown to decrease the acetylation of p53 in IMR32 neuroblastoma cells and protect cells against death caused by Aβ fragments [17]. The authors proposed that CWR spanned the binding pocket of resveratrol in the allosteric region of SIRT1 [18] and mediated the interaction between the N-terminal domain of SIRT1 and the acetylated substrate with fluorophore through hydrogen bonding and hydrophobic interactions. However, it is controversial whether resveratrol and the other compounds are true SIRT1 activators. For instance, several studies showed that the activities of resveratrol and the SRT compounds are due to their unphysiological binding to the fluorophore present after the acetyl-lysine of the substrates [19,20], while others demonstrated that the compounds promoted the deacetylation of substrates with specific amino acid sequences through an allosteric activation mechanism [21,22]. It has become apparent that the development of highly potent and selective SIRT1 activators is urgently needed for clinical applications.

In this study, we confirmed the effect of the tripeptide CWR on SIRT1. In contrast to the CWR activation mechanism proposed by Kumar et al. [17], we revealed that the tripeptide exhibited a novel activation mechanism by bonding covalently to the anomeric C1′ carbon of the ribose ring in OAADPr. CWR is highly selective for SIRT1, with no effect on SIRT2, SIRT3, SIRT5, or SIRT6. The longer distance between the anomeric C1′ carbon of the ribose ring in OAADPr and the Arg274 of SIRT1 (a conserved residue among sirtuins) than that between the anomeric C1′ carbon of the ribose ring in ADPr and the Arg of SIRT2, SIRT3, SIRT5, and SIRT6, should be responsible for the high selectivity of CWR for SIRT1. Elucidation of the activation mechanism of CWR, as well as the structural basis for the selectivity of the activator, provides a new perspective for designing potent and selective SIRT1 activators which could be effectively used in clinical settings.

## 2. Results and Discussion

### 2.1. Amino Acid Sequence Determines the Activity of CWR

**Figure 2:**
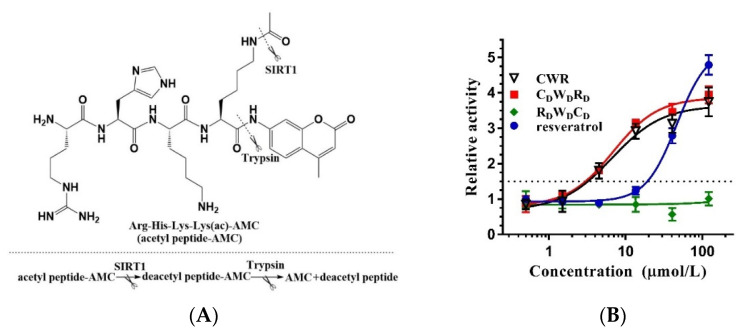
(**A**) The fluorescence assay that detects the deacetylation of an AMC-labeled acetyl peptide by SIRT1; (**B**) The dose–response curves of tripeptides and resveratrol measured by the fluorescence assay with an AMC-labeled acetyl peptide. For the relative activity, the initial enzyme activity is set to 1. When the enzyme activity reaches 1.5, the concentration represents EC_1.5_. The data are presented as means ± SD (*n* = 3).

The assay widely used for screening SIRT1 activators selected a “Fluor de Lys” (FdL) peptide, RHKK_ac_-AMC, as the substrate [10]. RHKK is derived from the amino acids at positions 379–382 of the SIRT1 endogenous substrate, p53. The lysine at position 382 is acetylated and attached to 7-amino-4-methyl coumarin (AMC) at the C-terminus, which constitutes FdL. However, the drawback of this assay is the unphysiological binding of the activator and the fluorophore AMC, which is recognized as an assay artifact [19]. When utilizing the activators identified through the FdL fluorescence assay for in vivo experiments, the activation activity may not be observed since the substrate of SIRT1 in its natural state does not have a fluorophore or equivalent chemical moiety immediately C-terminal to the acetyl-lysine. As a result, resveratrol is not considered a direct activator of SIRT1 [20]. To replicate the experimental results of Kumar et al. [17], we employed the FdL fluorescence assay to examine the activity of the tripeptide CWR (Figure 2A). The data revealed that CWR had an activation effect on SIRT1 with an EC_1.5_ of 3.16 μmol/L (Figure 2B), which was better than that of the control compound resveratrol (EC_1.5_ 19.2 μmol/L).

Interestingly, the activity of the peptide with D-type amino acids (C_D_W_D_R_D_) was comparable to that of CWR, whereas the reverse D-type peptide (R_D_W_D_C_D_) did not exhibit any activity. This indicated that the amino acid sequence of the peptide is crucial for its activity. To verify the effect of the amino acids’ chirality, we combined cysteine, tryptophan, and arginine with different chiralities (without changing the amino acid sequences) including C_D_WR, CW_D_R, CWR_D_, C_D_W_D_R, C_D_WR_D_, and CW_D_R_D_ (see Supplementary Materials for QC data). We tested their activities and found that D/L amino acids had little effect on the activation activity of CWR, each having a comparable EC_1.5_ to that of CWR (Table 1). These results clearly indicate that the activation activity of CWR is dependent on the amino acid sequence rather than the chirality, which led us to hypothesize that the reason for the loss of activation activity in R_D_W_D_C_D_ may be a clue to the activation mechanism of CWR, i.e., the recognition pocket of SIRT1 could accommodate the side chains of a peptide with a specific sequence, such as CWR. Alternatively, it could be possible that the cysteine at the amino end or the arginine at the carboxy end in CWR is critical for its activity.

### 2.2. CWR Activity Is Independent of AMC

**Figure 3:**
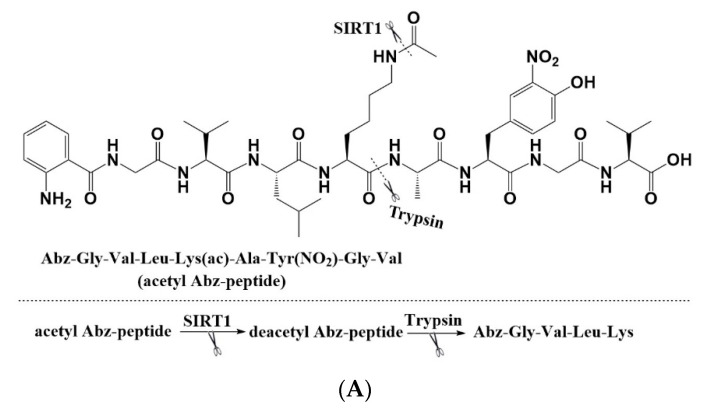

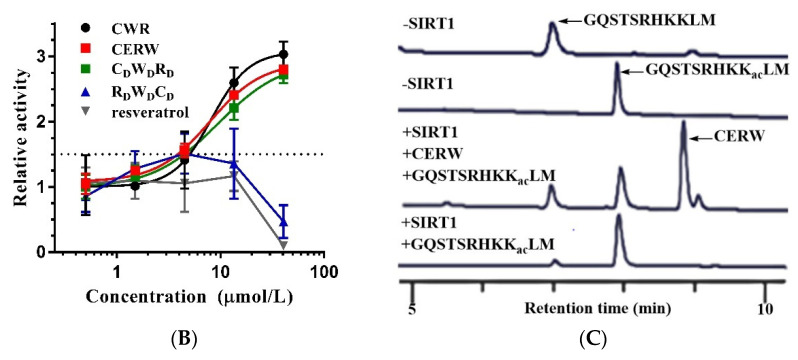
(**A**) The fluorescence assay that detects the deacetylation of an Abz-labeled acetyl peptide by SIRT1; (**B**) The dose–response curves of peptides and resveratrol measured by the fluorescence assay with an Abz-labeled acetyl peptide; the data are presented as means ± SD (*n* = 3); (**C**) Overlaid HPLC traces show that CERW promotes the deacetylation of GQSTSRHKK_ac_LM by SIRT1.

To exclude the possibility that the activation effect of CWR is due to the non-physiological binding of CWR and AMC, the activity of the peptide was verified with other assays and substrates. RHKK_ac_-AMC was replaced with the acetylated peptide Abz-GVLK_ac_AY(NO_2_)GV-NH_2_, derived from carbamoyl phosphate synthetase 1 (CPS1) (Figure 3A) [23]. CWR and C_D_W_D_R_D_ were still active, whereas R_D_W_D_C_D_ was not (Figure 3B). In comparison, we did not observe any activity of the control compound resveratrol, which confirmed that the activity of CWR is independent of the fluorophore AMC.

Abz-GVLK_ac_AY(NO_2_)GV-NH_2_ was modified with fluorophore 2-aminobenzoyl (Abz) at the amino end of the peptide. To examine the effect of the activator on the deacetylation of the substrate in its original unmodified state, we performed quantitative analysis of the SIRT1 reaction using high-performance liquid chromatography (HPLC). The retention times of the acetylated substrate derived from the p53 protein without any fluorophore (GQSTSRHKK_ac_LM) and of the deacetylated substrate (GQSTSRHKKLM) were 7.8 min and 7.0 min, respectively. In our preliminary experiment, we found that the retention times of CWR and GQSTSRHKKLM were indistinguishable, which impeded the quantitative analysis of GQSTSRHKKLM in the presence of CWR. Therefore, we synthesized another tetrapeptide, CERW, with a retention time clearly distinguishable from that of GQSTSRHKKLM, which displayed a better activity than CWR through the FdL fluorescence assay (Table 1) and Abz fluorescence assay (Figure 3B). As shown in Figure 3C, the peak area, at a retention time of 7.0 min for the experimental group with CERW, was significantly larger than that for the control group without CERW. This revealed that the tetrapeptide promoted deacetylation in the experimental group, which produced more deacetylated substrates than the control group under the same conditions. These results further confirmed that the activation activity of CERW is not dependent on the fluorophore behind the acetylated lysine of the substrate.

### 2.3. Cysteine of CWR Is Critical for Its Activity

**Figure 4:**
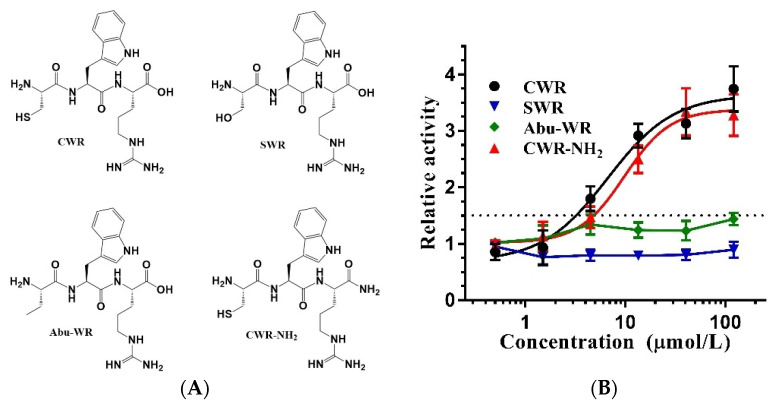
(**A**) Structures of CWR analogs; (**B**) The dose–response curves of tripeptide and its analogues measured by the fluorescence assay with an AMC-labeled acetyl peptide. The data are presented as means ± SD (*n* = 3).

The amino acid cysteine has a sulfhydryl group at the side chain, while serine has a hydroxy group instead. Other than that, there is no difference in their structures (Figure 4A). Considering the structural similarity between cysteine and serine, the substitution of cysteine with serine in CWR should improve its activity if cysteine in CWR interacts with SIRT1 via hydrogen bonding. Therefore, we synthesized tripeptide SWR and tested its effect on SIRT1 activity, but surprisingly, it did not show any effect (Figure 4B). This suggests that the sulfhydryl group of the cysteine in CWR is critical for SIRT1 activation and that the tripeptide is not bound to SIRT1 through hydrogen bonding, otherwise SWR should display activation activity.

In terms of hydrophobicity, the sulfhydryl group is stronger than the hydroxyl group; therefore, we hypothesized that CWR binds to SIRT1 through hydrophobic interactions. This led us to consider the amino acid derivative, 2-aminobutyric acid (Abu), which is structurally similar to cysteine (Figure 4A). When the sulfhydryl group of cysteine is replaced by a methyl group, it becomes Abu. We synthesized an Abu–WR peptide and examined its activation activity; however, no activity was observed (Figure 4B). Thus, hydrophobic interactions were ruled out. Considering that cysteine tends to form disulfide bonds, we speculated that CWR could form disulfide bonds intermolecularly or with the cysteine of SIRT1.

### 2.4. CWR Activity Is Unrelated to Disulfide Bonds

To examine whether CWR exerts its activation activity by forming dimers through intermolecular disulfide bonds, we synthesized a CWR–disulfide bond dimer and found it to be inactive (Table 1). Therefore, whether CWR forms a disulfide bond with SIRT1 became the focus of our next study.

**Figure 5:**
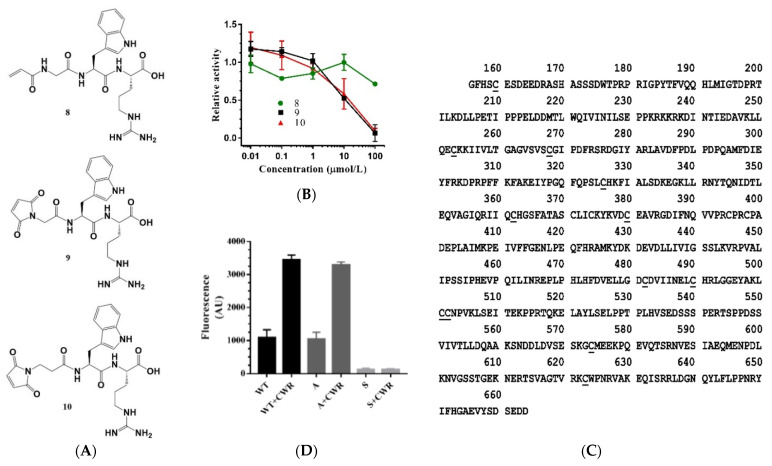
(**A**) Structures of CWR derivatives with chemical groups (acrylamide, maleimide) that can react with sulfhydryl; (**B**) The dose–response curves of the derivatives measured by the fluorescence assay with an AMC-labeled acetyl peptide; (**C**) Amino acid sequence of the SIRT1 protein, in which the cysteine that mutated to serine is marked with an underline; (**D**) CWR activities against SIRT1 and its mutants S and A at a concentration of 121.5 μmol/L. S is the mutant with all cysteines except for the cysteines in the zinc finger structure mutated to serine. A denotes the mutant of Ser362 in S mutated to alanine. The data are presented as means ± SD (*n* = 3).

In addition to the sulfhydryl group, which is known to covalently bond to the sulfhydryl group of cysteine, other chemical groups such as acrylamide and maleimide may also covalently bond to cysteine. For example, the acrylamide in afatinib, an irreversible EGFR inhibitor, forms a covalent bond with cysteine in the active site of EGFR [24]. Thus, we replaced the cysteine of CWR with other groups (acrylamide and maleimide) to obtain compounds 8, 9, and 10 (Figure 5A). Surprisingly, compound 8 did not exhibit any activity, whereas compounds 9 and 10 exhibited inhibitory effects (Figure 5B). These results suggest that covalent bonding with the cysteine residue(s) of SIRT1 may not activate SIRT1 and may even inhibit its activity, implying that covalent bonding with the cysteine residue(s) of SIRT1 might not be the reason for the activity of CWR.

To further verify whether the disulfide bond with SIRT1 is responsible for the activity of CWR, we mutated the cysteine residues of SIRT1 to serine residues (denoted as mutant S), excluding the four cysteine residues necessary for the zinc finger structure (Figure 5C). Surprisingly, the deacetylase activity of mutant S vanished, which led us to question whether one or several cysteine residues in SIRT1 are crucial for enzyme activity. We analyzed SIRT1 residues near the mutant cysteine residues and found that His363 of SIRT1 was adjacent to Cys362. His363 is a conserved residue in sirtuins and is essential for their activity [6]. In a previous study, it was shown that in the absence of an acetylated peptide substrate, the imidazole ring of the conserved His248 occupied the acetyl group binding site in SIRT3, but rotated to a state parallel to the acetyl group once the acetylated substrate was bound to SIRT3 [25]. Therefore, we proposed that when Cys362 of SIRT1 is mutated to a serine, it may easily form hydrogen bonds with His363, thus stabilizing the conformation of the imidazole ring of His363 so that rotation cannot occur when the acetylated substrate binds SIRT1. This ultimately inhibits the binding of the acetylated substrate to SIRT1, thereby diminishing the deacetylase activity of the sirtuin. In addition, we found that the Cys362 residue in SIRT1 is not conserved among sirtuins. The corresponding residues in SIRT2/3/4/5/6/7 are alanine/alanine/leucine/isoleucine/leucine/leucine, respectively. Thus, we mutated Ser362 from S to alanine to construct mutant A. In agreement with our hypothesis, the enzyme activity was restored. Notably, CWR exhibited activation activity in mutant A (Figure 5D). Therefore, we concluded that CWR activity is unrelated to disulfide bond formation.

The above results show that the cysteine of CWR is crucial for its activity; however, it does not form hydrogen bonds, hydrophobic interactions, or disulfide bonds with SIRT1. Considering that in SIRT1-catalyzed deacetylation, there are substrates (the acetylated peptide and NAD^+^) as well as products (nicotinamide and OAADPr), it is possible that CWR may bind to one of them to activate SIRT1, as its mechanism of action.

### 2.5. Covalent Bonding of OAADPr and Cysteines of CWR

**Figure 6:**
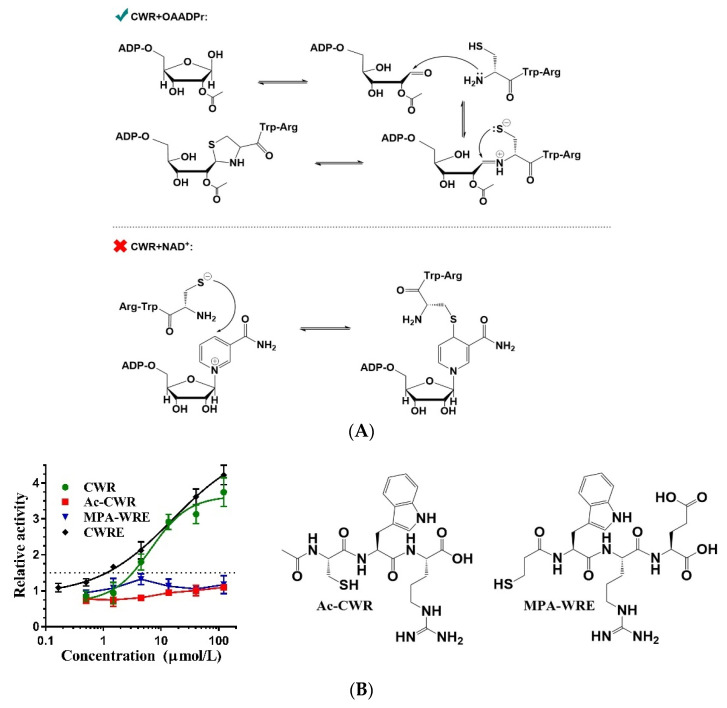
(**A**) The putative mechanisms for covalent bonding of CWR and OAADPr or NAD^+^; (**B**) The dose–response curves of CWR, CWRE, and their analogues measured by the fluorescence assay with an AMC-labeled acetyl peptide. Ac-CWR, acetylation at the N-terminus of CWR; MPA, 3-mercaptopropionic acid. The data are presented as means ± SD (*n* = 3).

It has been reported that cysteine can covalently bond to NAD^+^ and ADPr [26,27]. In terms of NAD^+^ (a substrate of SIRT1-catalyzed deacetylation), previous studies have demonstrated that the sulfhydryl group of cysteine and the C4 atom of the nicotinamide ring of NAD^+^ form a covalent bond [26,28]. For OAADPr (a product of SIRT1-catalyzed deacetylation), the anomeric C1′ carbon of the ribose ring can be covalently bound to both the N-terminal α-amino nitrogen and the side chain sulfur of cysteine to form a thiazolidine ring [27,29]. Therefore, we hypothesized that CWR may form a covalent bond with NAD^+^ or OAADPr to promote SIRT1-catalyzed deacetylation (Figure 6A). Comparing their differences, the formation of a covalent bond between cysteine and NAD^+^ is a simple substitution reaction, while the covalent bond of cysteine and OAADPr involves ring opening as well as ring formation, in which the N-terminal α-amino nitrogen is necessary. This would be a breakthrough in elucidating the essential force of CWR to activate SIRT1. To test this hypothesis, we synthesized an N-terminally acetylated CWR, which led to the disappearance of the activation activity (Figure 6B). In contrast, the C-terminal amidation peptide of CWR retained its activity (Figure 4B). To exclude any possible steric hindrance from the N-terminal acetyl in Ac-CWR, we replaced the cysteine with 3-mercaptopropionic acid (MPA) and synthesized the analogue of CWRE (Table 1), named as MPA-WRE. As shown in Figure 6B, removal of -NH_2_ from the cysteine abolished the activation activity. Consistent with the loss of the activation activity of R_D_W_D_C_D_, these results proved that the covalent bonding of CWR and OAADPr is essential for SIRT1 activation.

**Figure 7:**
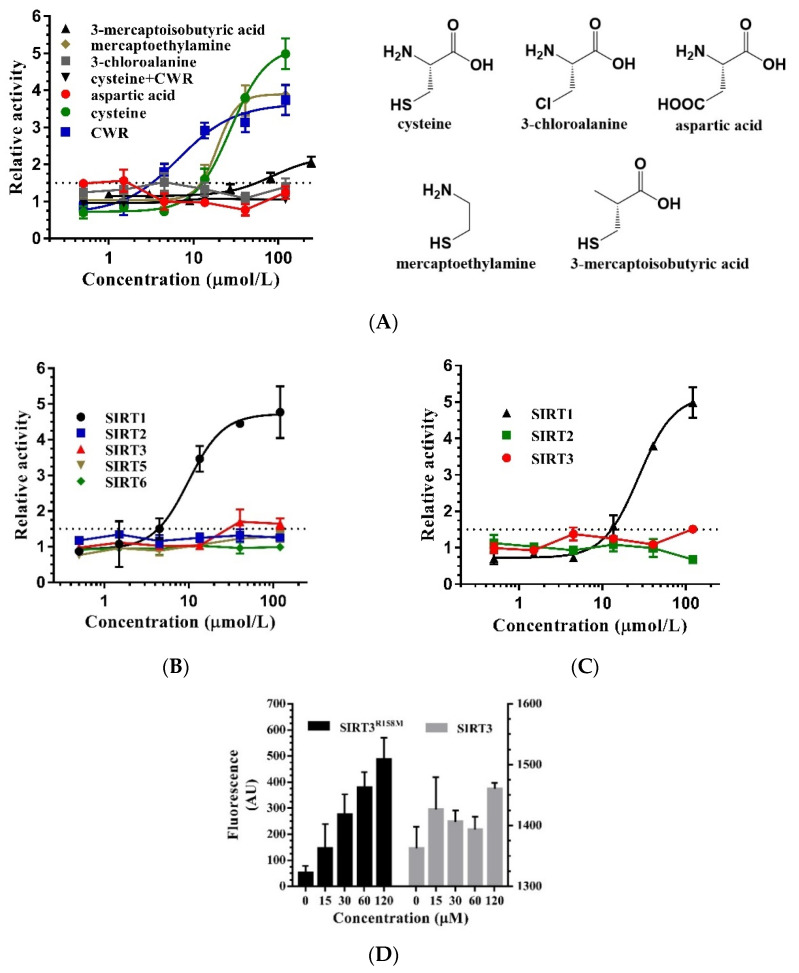
(**A**) The dose–response curves of cysteine and its analogues measured by the fluorescence assay with an AMC-labeled acetyl peptide. “Cys + CWR” represents the addition of CWR to the sample with excessive cysteine (243 μmol/L) to test whether CWR can further activate SIRT1; (**B**) The dose–response curves of CWR with SIRT1, SIRT2, SIRT3, SIRT5, SIRT6, measured by the fluorescence assay with an AMC-labeled acetyl peptide; (**C**) The dose–response curves of cysteine with SIRT1, SIRT2, SIRT3, measured by the fluorescence assay with an AMC-labeled acetyl peptide; (**D**) The dose–response histogram of CWR with SIRT3 and SIRT3^R158M^, measured by the fluorescence assay with an AMC-labeled acetyl peptide. The data are presented as means ± SD (*n* = 3).

To further confirm the covalent bonding of OAADPr and the cysteine of CWR, we examined the effect of cysteine and its analogues on the deacetylation catalyzed by SIRT1. Cysteine showed a relatively lower activation activity than CWR, whereas substitution of sulfhydryl or an amino group of cysteine abolished the activation activity, but substitution of a carboxyl group did not affect the activity (Figure 7A). Furthermore, the addition of CWR to the SIRT1-catalyzed deacetylation sample with excessive cysteine did not enhance deacetylation. These results suggest that CWR and cysteine adopt the same mechanism to promote SIRT1 activity by forming covalent bonds with OAADPr.

### 2.6. Mechanism of CWR Selectivity

**Figure 8:**
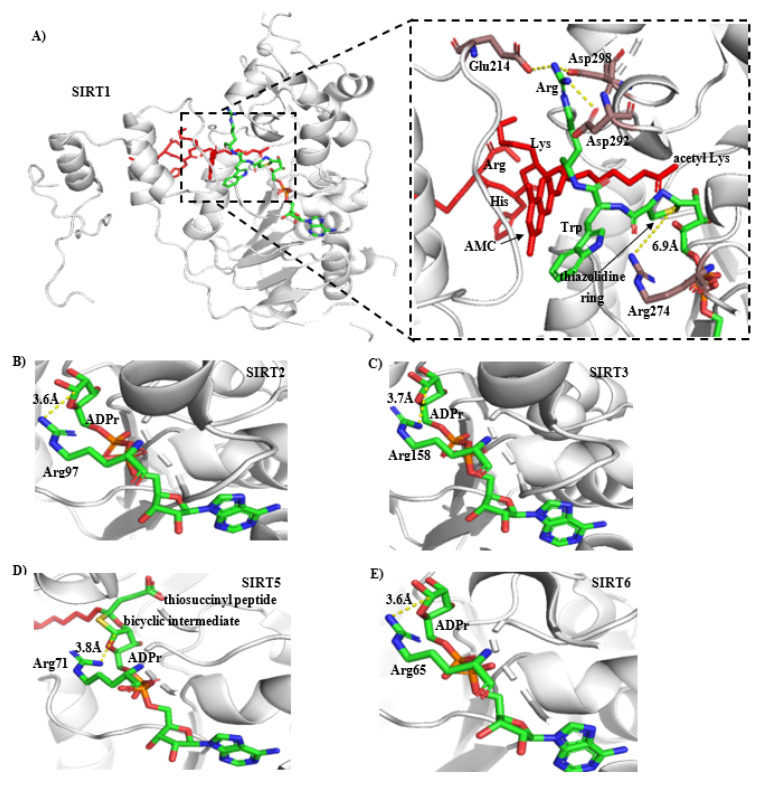
(**A**) Bind model of CWR in SIRT1. The structure in red represents the peptide substrate RHKK_ac_-AMC. The atoms of C, N, S, O are in green, blue, yellow, and red, respectively, except for the C atoms of Glu214, Asp292, Asp 298, Arg274 in SIRT1, which are shown in brown. The dashed lines in yellow indicate electrostatic interactions or distances between atoms; (**B**–**E**) The distances between the conserved arginine in SIRT2, SIRT3, SIRT5, or SIRT6 and the anomeric C1′ carbon of the ribose ring in ADPr or the ADPr bicyclic intermediate.

Each sirtuin has unique roles in biological processes; thus, the selectivity should be considered when developing SIRT1 activators. We tested the activation activity of CWR as well as cysteine on SIRT2/3/5/6 and found that neither had any effect on these sirtuins (Figure 7B,C). These results suggest that the high selectivity of CWR or cysteine for SIRT1 originates from the SIRT1 region, where the cysteine and OAADPr are covalently bound, and this region must be different between SIRT1 and SIRT2/3/5/6. Thus, we docked CWR into SIRT1 via the formation of a thiazolidine ring by the sulfhydryl group and ADPr (Figure 8A). We found that the arginine in CWR is surrounded by the acidic amino acids Glu214, Asp292, and Asp298 and forms electrostatic interactions with the residues, which could explain the lower EC_1.5_ of CWR than that of cysteine and CWN (Table 1).

According to the CWR binding model, the amino acids surrounding the thiazolidine ring are highly conserved among SIRT1/2/3/5/6. However, we noticed that the distances between the anomeric C1′ carbon of the ribose ring in ADPr and a conserved arginine residue (Arg274 in SIRT1) are different. The distance between the Arg274 residue of SIRT1 and the anomeric C1′ carbon of the ribose ring in ADPr is 6.9 Å, while the distance between the corresponding arginine residues in SIRT2 (PDB ID: 5D7O), SIRT3 (PDB ID: 4BN4), SIRT5 (PDB ID: 4F56), SIRT6 (PDB ID: 3PKI) to the anomeric C1′ carbon is 3.6 Å, 3.7 Å, 3.8 Å, and 3.6 Å, respectively (Figure 8B–E). We speculated that in SIRT2/3/5/6, the interaction between the conserved arginine and OAADPr prohibits the attack on the anomeric C1′ carbon of the ribose ring in OAADPr by the N-terminal α-amino nitrogen and sulfhydryl of the cysteine, thus preventing the formation of the thiazolidine ring. This would explain the activation selectivity of CWR and cysteine for SIRT1.

To test this hypothesis, we mutated Arg158 of SIRT3 to methionine and obtained mutant SIRT3^R158M^. Interestingly, the enzymatic activity of SIRT3^R158M^ was abolished, indicating that the conserved Arg158 residue is critical for the enzymatic activity of SIRT3. When CWR was added to the reaction samples, CWR restored the enzyme activity and activated SIRT3^R158M^ in a concentration-dependent manner (Figure 7D). This indicated that the selectivity of CWR was due to the different distances between the conserved arginine in SIRT1/2/3/5/6 and the anomeric C1′ carbon of the ribose ring in OAADPr.

### 2.7. CWR Activation Mechanism

To reveal the mechanism by which CWR promotes the enzymatic activity of SIRT1, we performed enzyme kinetic assays and found that CWR decreased the value of K_m_ in the acetylated substrate but not in NAD^+^ (Table 2). Therefore, we envisage the activation as follows: NAD^+^ first binds to SIRT1, and then the acetylated substrate binds to the “SIRT1/NAD^+^” complex, at which point the acetyl-lysine binds to the D-pocket, and the nicotinamide-moiety of NAD^+^ is shifted to the C-pocket of SIRT1 [30]. Subsequently, when the acetyl-lysine forms an intermediate with ADPr in SIRT1 [31], CWR binds to “SIRT1/the acetyl-lysine–ADPr intermediate” and forms reversible covalent bonds with O-acetyl-ADPr. When the deacetylated substrate is released from SIRT1, the adduct of “CWR/O-acetyl-ADPr” is also released. Previously, we confirmed that a conserved arginine in sirtuins (i.e., Arg274 of SIRT1, Arg158 of SIRT3) is critical for enzyme activity, and we deduced that this conserved arginine might interact with O-acetyl-ADPr and pull it out of the enzyme. According to the crystal structures of SIRT1, SIRT2, SIRT3, SIRT5, and SIRT6 in complex with ADPr or the ADPr bicyclic intermediate, the conserved arginine, Arg274 in SIRT1, is more distant from O-acetyl-ADPr than the corresponding arginine in other sirtuins; thus, CWR may have the opportunity to form an adduct with O-acetyl-ADPr, which contributes to the release of O-acetyl-ADPr from SIRT1. In this way, CWR promotes the SIRT1-catalyzed deacetylation reaction.

**Figure 9:**
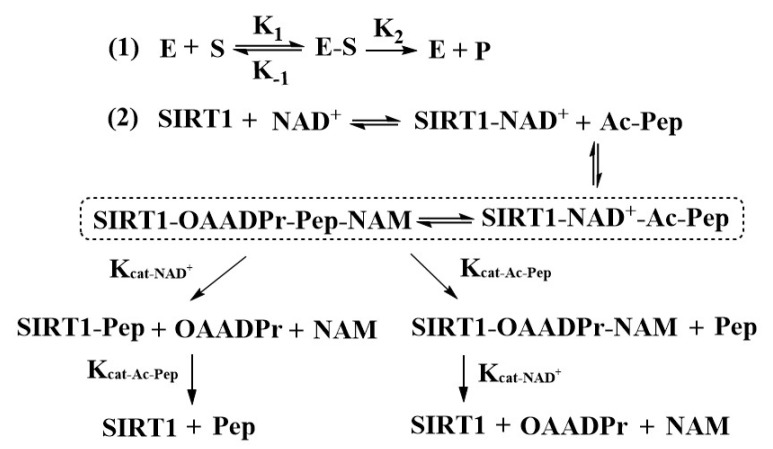
Reaction flow of SIRT1-catalyzed substrate deacetylation. E: enzyme; S: substrate; P: product; NAM: nicotinamide; Ac-Pep: acetylated peptide; and Pep: deacetylated peptide.

Further, as shown in Figure 9, Formula (1) represents the classic enzyme reaction in which the release of P is the rate-limiting step. Notably, the Michaelis–Menten constant K_m_ = (K_-1_ + K_2_)/K_1_, where K_2_ is the K_cat_, and since K_-1_ >> K_2_, thus K_m_ = K_-1_/K_1_, which represents the binding affinity of E and S. In the case of the SIRT1-catalyzed deacetylation reaction represented by Formula (2), the binding of CWR did not affect the K_m,NAD+_, indicating that CWR did not bind into SIRT1 at this step, otherwise the value of K_m,NAD+_ should be decreased. Next, acetyl-substrate (i.e., Ac-Pep) binds to SIRT1-NAD^+^ and forms the SIRT1-OAADPr-Pep-NAM, at which point CWR binds to SIRT1-OAADPr-Pep-NAM and decreases the K_m,Ac-Pep_. Thereafter, although the order of release of OAADPr and the deacetylated product remains unclear, CWR significantly increased the value of K_cat-NAD+_ over that of K_cat-Ac-Pep_ (Table 2), implying that CWR facilitated the release of OAADPr. This is further evidence supporting our proposed activation mechanism.

### 2.8. Tripeptide Promotes p53 Deacetylation in Cells via SIRT1

**Figure 10:**
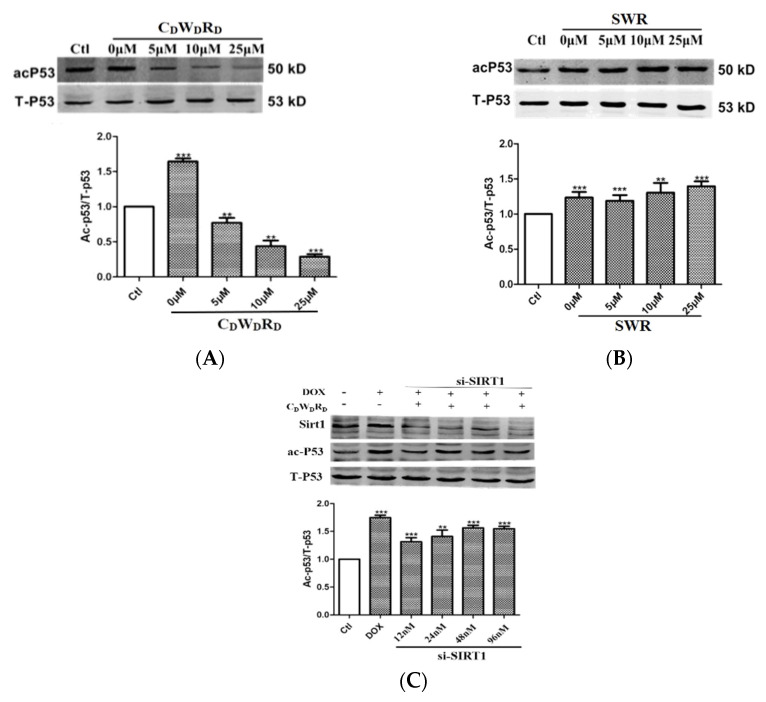
Effects of C_D_W_D_R_D_ and SWR on acetylation of p53 in cells. (**A**,**B**) The experimental groups were treated with 10 μmol/L DOX, while the control group was treated an equal volume of DMSO, then the experimental groups were added with 0 μmol/L, 5 μmol/L, 10 μmol/L, and 25 μmol/L tripeptides, where 0 μmol/L indicates the addition of an equal volume of ultrapure water; (**C**) Effect of C_D_W_D_R_D_ on p53 acetylation with SIRT1 knockdown. The DOX group was treated with 10 μmol/L DOX, while the control group was treated with an equal volume of DMSO, the experimental groups were treated with 10 μmol/L DOX and 25 μmol/L C_D_W_D_R_D_, and SIRT1 siRNA were treated with concentrations of 12 nmol/L, 24 nmol/L, 48 nmol/L, and 96 nmol/L. The error bars represent means ± SEM (*n* = 3). ** *p* < 0.01, *** *p* < 0.001, compared with the control group. One-way ANOVA with Newman–Keuls post hoc test.

As p53 is a substrate of SIRT1, modulating the activity of SIRT1 may alter the acetylation level of p53 [32]. To examine the activity of the tripeptide activator at the cellular level, we treated a human breast cancer cell line, MCF-7, with C_D_W_D_R_D_/SWR and doxorubicin (DOX), which induces apoptosis and produces large amounts of acetylated p53. The results showed that the p53 acetylation levels gradually decreased with increasing concentrations of C_D_W_D_R_D_, whereas SWR had no effect (Figure 10A,B).

Next, we examined whether the tripeptide activator promoted p53 deacetylation via SIRT1 in MCF-7 cells using siRNA to knockdown SIRT1 expression. SIRT1 levels gradually decreased with increasing SIRT1 siRNA concentrations (Figure 10C), indicating that the SIRT1 knockdown was successful. In addition, the effect of C_D_W_D_R_D_ on reducing p53 acetylation gradually diminished as the concentration of SIRT1 siRNA increased. This reveals that C_D_W_D_R_D_ decreases the acetylation level of p53 by selectively activating SIRT1 in cells.

## 3. Experimental Section

### 3.1. Protein Expression and Purification

Human SIRT1(156–664) was cloned into a pET28a vector between NdeI and XhoI with a hexa-histidine-tag at the N-terminal. The protein was expressed in *Escherichia coli* BL21(DE3) cells. A single colony was inoculated in LB medium containing 50 μg/mL of kanamycin at 37 °C. When OD_600_ reached 0.6, the culture was then transferred to 16 °C. As OD_600_ reached about 0.8, 0.5 mmol/L IPTG was added into the culture, and the protein expression was induced at 16 °C overnight. Cells were then collected by centrifugation, and the cell pellet was resuspended with lysis buffer (20 mmol/L Tris-HCl, pH 8.0, 200 mmol/L NaCl, 5 mmol/L β-mercaptoethanol) and sonicated to break the cells. The supernatant was separated from cell debris by centrifugation at 13,000× *g* for 30 min at 4 °C and loaded on Ni-NTA resin that was pre-equilibrated with the lysis buffer containing 20 mmol/L imidazole. After washing the unbound proteins with 40 mmol/L imidazole in lysis buffer, SIRT1 was eluted with 250 mmol/L imidazole in the lysis buffer and dialyzed in the deacetylation assay buffer mixed with glycerol (i.e., 25 mmol/L Tris-HCl, pH 8.0, 137 mmol/L NaCl, 2.7 mmol/L KCl, 1 mmol/L MgCl_2_, 10% glycerol). SIRT1 mutants S and A were expressed and purified as the wild-type SIRT1(156–664). The purity of SIRT1 and its mutants was assessed by SDS-PAGE.

Human SIRT2(1–356) was cloned into a pGEX-4T-1 vector between BamH1 and EcoR1 with a GST-tag at the N-terminal. The protein was expressed in *Escherichia coli* BL21(DE3) cells. A single colony was inoculated in LB medium containing 100 μg/mL of ampicillin at 37 °C. When OD_600_ reached 0.8, 0.5 mmol/L IPTG was added into the culture and the protein expression was induced at 37 °C for 5 h. Cells were then collected by centrifugation, and the cell pellet was resuspended with lysis buffer (20 mmol/L Tris-HCl, pH 8.0, 200 mmol/L NaCl, 5 mmol/L β-mercaptoethanol) and sonicated to break the cells. The supernatant was separated from cell debris by centrifugation at 13,000× *g* for 30 min at 4 °C and loaded on GSTrap™ HP pre-equilibrated with the lysis buffer. After washing the unbound proteins with lysis buffer, SIRT2 was eluted with 10 mmol/L GSH in lysis buffer and dialyzed in the deacetylation assay buffer mixed with glycerol (i.e., 25 mmol/L Tris-HCl, pH 8.0, 137 mmol/L NaCl, 2.7 mmol/L KCl, 1 mmol/L MgCl_2_, 10% glycerol). Human SIRT3, SIRT5, and SIRT6 were expressed and purified as previously described [33,34,35].

### 3.2. Screening of Sirtuin Activators and EC_1.5_ Determination

To measure the activation activity of compounds for SIRT1, SIRT2, SIRT3, SIRT5, and SIRT6, the acetylated peptide with the sequence of RHKK_ac_ derived from residues 379–382 of the p53 protein was used as the substrate of the deacetylation reaction. 7-amino-4-methyl coumarin (AMC) was attached at the C-terminus of the peptide substrate. The reaction was carried out with 0.5 μmol/L SIRT1, 750 μmol/L NAD^+^, 31.25 μmol/L RHKK_ac_-AMC, and different concentrations (0.5 μmol/L, 1.5 μmol/L, 4.5 μmol/L, 13.5 μmol/L, 40.5 μmol/L, 121.5 μmol/L) of the compounds in the deacetylation assay buffer (i.e., 25 mmol/L Tris-HCl, pH 8.0, 137 mmol/L NaCl, 2.7 mmol/L KCl, 1 mmol/L MgCl_2_) at 37 °C for 45 min. Then, trypsin (0.5 mg/mL) containing 10 mmol/L of nicotinamide was added and incubated at 37 °C for 30 min. The fluorescence was measured at an excitation wavelength of 340 nm and an emission wavelength of 490 nm with a microplate reader (POLARstar OPTIMA, BMG Labtech).

Alternatively, an acetylated peptide (Abz-GVLK_ac_AY(NO_2_)GV-CA) derived from carbamoyl phosphate synthetase 1 (CPS1) was used as the substrate. The reaction was carried out with 0.05 μmol/L SIRT1, 500 μmol/L NAD^+^, 10 μmol/L Abz-GVLK_ac_AY(NO_2_)GV-NH_2_, and different concentrations (0.5 μmol/L, 1.5 μmol/L, 4.5 μmol/L, 13.5 μmol/L, 40.5 μmol/L, 121.5 μmol/L) of the compound in the deacetylation assay buffer at 37 °C for 30 min. Then, trypsin (0.01 mg/mL) containing 10 mmol/L of nicotinamide was added and incubated at 37 °C for 15 min. The fluorescence was measured at an excitation wavelength of 320 nm and an emission wavelength of 420 nm. EC_1.5_ were analyzed using GraphPad 7.0. For SIRT2, SIRT3, SIRT5, and SIRT6, the activity of the compounds was tested like SIRT1, with concentrations of 0.62 μmol/L, 1.0 μmol/L, 1.2 μmol/L, 5.0 μmol/L, respectively. All reactions were performed in triplicate.

### 3.3. HPLC Assay

The SIRT1 deacetylation reaction submitted for HPLC assaying was performed as above without the addition of trypsin and nicotinamide. Briefly, SIRT1 (0.1 μmol/L), peptide GQSTSRHKK_ac_LM (31.25 μmol/L) and 750 μmol/L NAD^+^ in the presence and absence of CWR (40.5 μmol/L) were incubated in the assay buffer (25 mmol/L, pH 8.0) at 37 °C for 45 min. The deacetylation reaction was terminated by adding 6 μL of cold 10% formic acid and centrifuged for 10 min. The samples were analyzed by a Dionex UltiMate 3000 HPLC system. The separation was achieved at 25 °C by using the Zorbax Extend-C18 column (150 mm × 4.6 mm, PN:763953-902). The mobile phase consisted of buffer A (0.065% trifluoroacetic acid/water, *v*/*v*) and buffer B (0.05% trifluoroacetic acid/acetonitrile, *v*/*v*) at a flow rate of 1.0 mL/min. The deacetylated product (GQSTSRHKKLM) and the substrate (GQSTSRHKK_ac_LM) peaks were monitored and subsequently quantified by measuring the area under the curve.

### 3.4. Enzyme Kinetics Assay

To determine the effect of the activator on the binding of SIRT1 with the acetylated substrate and NAD^+^, the concentrations of SIRT1 and NAD^+^ were fixed at 0.5 μmol/L and 750 μmol/L, respectively, and the concentrations of the acetyl peptide substrate were set at 40 μmol/L, 80 μmol/L, 160 μmol/L, 320 μmol/L, 640 μmol/L, and 1280 μmol/L, and the concentrations of CWR/SWR at 40.5 μmol/L. The reaction started at 6, 12, 18, 24, and 30 min in the deacetylation assay buffer, respectively. Then, trypsin (0.5 mg/mL) containing 10 mmol/L of nicotinamide was added and incubated at 37 °C for 30 min. The fluorescence was measured at an excitation wavelength of 340 nm and an emission wavelength of 490 nm. Michaelis–Menten was analyzed using GraphPad 7.0. The effect of CWR/SWR on the binding of SIRT1 and NAD^+^ was determined similarly except that the concentrations of SIRT1 and the acetyl peptide substrate were fixed at 0.5 μmol/L and 500 μmol/L, respectively. The concentrations of NAD^+^ were set to 40 μmol/L, 80 μmol/L, 160 μmol/L, 320 μmol/L, 640 μmol/L, and 1280 μmol/L. All reactions were performed in triplicate.

### 3.5. Molecular Docking

The coordinates of SIRT1/RHKK_ac_-AMC were extracted from the crystal structure of SIRT1/RHKK_ac_-AMC/resveratrol (PDB ID: 5BTR). After alignment of SIRT1/RHKK_ac_-AMC to the structure of SIRT1/ADPr (PDB ID: 4KXQ), ADPr was merged into the structure of SIRT1/RHKK_ac_-AMC. The structure of CWR was generated in Discovery Studio 2.5, and manually set into SIRT1/RHKK_ac_-AMC/ADPr to form a thiazolidine ring between the cysteine and the anomeric C1′ carbon atom of the ribose of ADPr. The structure of SIRT1/RHKK_ac_-AMC/ADPr/CWR was assigned with a CHARMm force field. The non-hydrogen atoms of SIRT1, RHKK_ac_-AMC, and ADPr in the structure were constrained. The structure was optimized with the default parameters in the energy minimization module until the RMS gradient was less than 0.01. The optimized structure was then submitted to the standard cascade of molecular dynamics simulations with a target temperature of 300 kelvin. The SHAKE constraint was not applied. The time for the heating and equilibration procedures was 20 ns. The conformation of “SIRT1/RHKK_ac_-AMC/ADPr/CWR” was sampled every 1ps during the NVT-type molecular dynamics simulation of 1μs. The final structure obtained from the molecular dynamics simulation was optimized without any structural constraint in the energy minimization module as described above.

### 3.6. Cell Culture

The human breast cancer cell line MCF-7 was grown in DMEM (Gibco, catalog no.: 12430–054) plus 10% FBS (Gibco, catalog no.: 10091-148) at 37 °C. Cells were incubated in the humidified incubator containing 5% CO_2_. To test the effect of the activators on the acetylation level of p53, MCF-7 cells were treated with tripeptides (5 μmol/L, 10 μmol/L, 25 μmol/L) and 10 μmol/L of DOX for 6 h. As the control, no DOX and activator were incubated with MCF-7 cells for 6 h. Then, the cells were washed with PBS three times and were harvested for western blot analysis.

### 3.7. Transient Transfection

250 μL opti culture medium containing 8 μL of liposomal lipo 2000 was added to another 250 μL opti culture medium containing SIRT1 siRNA (12 nmol/L, 24 nmol/L, 48 nmol/L, 96 nmol/L). After mixing thoroughly and resting for 20 min, they were added to MCF-7 cells containing 4.5 mL DMEM culture medium, which was incubated at 37 °C with 5% CO_2_ for 4 h. The culture medium was discarded and replaced with 5 mL DMEM culture medium containing 10% FBS, and continued to incubate for 12 h. The culture medium was discarded and replaced with 5 mL DMEM culture medium without serum, and 25 μmol/L of C_D_W_D_R_D_ and 10 μmol/L of DOX were incubated with them for 6 h. As the control, no DOX and activator were incubated with MCF-7 cells for 6 h, while 10 uM of DOX was incubated with MCF-7 cells for 6 h as the DOX group. These were harvested for western blot analysis.

### 3.8. Western Blotting

Cells were harvested in 10 mmol/L Tris buffer (pH 7.4) containing 1% SDS and a cocktail inhibitor (Roche, catalog no.11836170001). Equal amounts of protein were electrophoresed on 10% SDS-PAGE and transferred to nitrocellulose membranes. The membrane was incubated with a primary antibody (Anti-p53 antibody, Abcam, catalog no.: ab75754; Anti-p53 (acetyl K382) antibody, Abcam, catalog no.: ab75754) diluted in TBS containing 5% BSA and 0.05% Tween-20 overnight, and then incubated with peroxidase-conjugated secondary antibodies (Goat anti-mouse IgG (H&L), Aksomics, catalog no.: KC-MM-035; Goat anti-rabbit IgG(H&L), Aksomics, catalog no.: KC-RB-035) for 2 h at room temperature. The membrane was processed by using Pierce™ ECL Western Blotting Substrate (Bioscience, catalog no.: HLX-P82011). The bands on the film were quantified with Quantity One (Bio-Rad).

## 4. Conclusions

SIRT1 is an attractive therapeutic target for age-related diseases. Considerable efforts have been directed towards the development of both the activators and inhibitors of this enzyme. However, many SIRT1 activators suffer from low bioavailability and potency, poor pharmacokinetics, or a limited target specificity [12,13,36,37,38]. Therefore, the development of SIRT1 activators with better pharmacokinetics, tolerability, and selectivity is essential. Peptidic or peptidomimetic drugs, on the other hand, may be an option due to their druggability, potent biological activity, high specificity, and low toxicity. In this study, we confirmed the activation activity of the tripeptide CWR on SIRT1. We demonstrated that unlike resveratrol, CWR activity does not rely on the fluorophore immediately C-terminal to the acetylated lysine of the substrate. Furthermore, we elucidated that CWR adopts a novel mechanism to activate SIRT1 by covalently bonding to the anomeric C1′ carbon of the ribose ring in OAADPr, the product of SIRT1-catalyzed deacetylation. In addition, CWR is highly selective for SIRT1 and had no effect on SIRT2, SIRT3, SIRT5, or SIRT6. According to the binding mode of CWR in SIRT1, the Arg274 residue of SIRT1 (conserved in sirtuins) is more distant from the anomeric C1′ carbon of the ribose ring in ADPr than in SIRT2/3/5/6. This distance is unfavorable for the interaction of Arg274 with OAADPr. Thus, cysteine could form a thiazolidine ring with the anomeric C1′ carbon of ribose in OAADPr. This is also the key structural basis for the high selectivity of CWR for SIRT1, which was confirmed by site-directed mutagenesis of SIRT3. Consistent with in vitro assays, C_D_W_D_R_D_ significantly attenuated the acetylation of p53 in MCF-7 cells via SIRT1. Previously, it had been shown that CWR was non-toxic to human erythrocytes by the hemolytic assay [16]. It is well known that D-type peptides have longer gastrointestinal, plasma, and intracellular half-lives than L-type peptides [39,40,41,42]. Here we showed that substitution of each residue of the peptide with D-type amino acids did not abolish the activity (Table 1), rending C_D_W_D_R_D_ and its related derivatives as promising lead compounds for drug development targeting SIRT1. Elucidation of the activation mechanism of CWR, as well as the structural basis for the selectivity of the activator, also sheds light on designing SIRT1-selective activators whose activity does not rely on the chemical moiety immediately C-terminal to the acetyl-lysine of the substrate.

## Figures and Tables

**Table 1:** Activation activity of the peptides and resveratrol to wild-type SIRT1.

Compounds	EC_1.5_ (μmol/L) ^a^	Max A (%) ^b^
Resveratrol	19.2 ± 1.60	528 ± 26.0
CWR	3.16 ± 0.35	364 ± 20.5
C_D_W_D_R_D_	2.99 ± 0.38	386 ± 12.5
C_D_WR	3.18 ± 0.21	295 ± 32.9
CW_D_R	5.17 ± 0.71	275 ± 32.1
CWR_D_	3.11 ± 0.32	374 ± 15.1
C_D_W_D_R	3.02 ± 0.20	333 ± 20.3
C_D_WR_D_	3.71 ± 0.17	320 ± 18.8
CW_D_R_D_	4.57 ± 0.27	344 ± 23.4
R_D_W_D_C_D_	NA ^c^	NA
CWN	10.0 ± 1.23	210 ± 11.4
SWR	NA	NA
S_D_W_D_R_D_	NA	NA
CERW	1.98 ± 0.10	306 ± 20.3
CWRE	1.06 ± 0.07	523 ± 44.5
CWR dimer	NA	NA

^a^ EC_1.5_: the concentration of an activator required to increase the enzyme activity by 50%; ^b^ Max A: percentage of maximum activation achieved; ^c^ NA: ‘not active’. The data are presented as means ± SD (*n* = 3).

**Table 2:** The enzyme kinetic parameters with/without tripeptide.

SIRT1 + Pep	RHKK_ac_-AMC			NAD^+^		
	K_cat_	K_m_	K_cat_/K_m_	K_cat_	K_m_	K_cat_/K_m_
	(S^−1^)	(μmol/L)	(S^−1^ mmol^−1^L)	(S^−1^)	(μmol/L)	(S^−1^ mmol^−1^L)
SIRT1	0.61 ± 0.03	223.1 ± 38.22	2.740 ± 0.78	0.65 ± 0.05	203.2 ± 39.74	3.20 ± 1.25
SIRT1 + CWR	0.79 ± 0.05	72.70 ± 20.39	10.90 ± 2.45	1.34 ± 0.05	202.8 ± 24.45	6.63 ± 2.04
SIRT1 + SWR	0.61 ± 0.05	225.9 ± 32.79	2.690 ± 1.52	0.66 ± 0.04	202.3 ± 37.89	3.26 ± 1.05

K_m_ is the Michaelis–Menten constant, K_cat_ is the catalytic constant; Pep: peptide. The data are presented as means ± SD (*n* = 3).

## Data Availability

The datasets created and analyzed during the current study are available from the corresponding author upon reasonable request.

## Supplementary Materials

The following supporting information can be downloaded at: https://www.mdpi.com/article/10.3390/molecules27092714/s1, QC data for the compounds covered in the article.

## Sample Availability Statement

Samples of the compounds are not available from the authors.
