# Supplementary material for: A Novel Mechanism for SIRT1 Activators That Does Not Rely on the Chemical Moiety Immediately C-Terminal to the Acetyl-Lysine of the Substrate
Source: Molecules. 2022 Apr 22;27(9):2714. doi: 10.3390/molecules27092714 (PMC9099470; doi:10.3390/molecules27092714)
Supplement: Supplementary file 1 [file molecules-27-02714-s001.zip › molecules-1676525-supplementary.pdf]

Quality Inspection Report

**Product Name:**	PA1702
**Lot Num:**	OP011920SF-002
**Sequence:**	CWR 分子间二硫键成环
**Molecular Weight:**	923.1
**Mass Spectral Analysis:**	see attached MS spectrogram
**HPLC Analysis:**	Peptide purity：93.57%
**Column :**	agilent ZORBAX 300SB-C18 5um 4.6*250mm
**Mobile phase:**	A:0.1%TFA in 100%H2O<br>B:ACN
**Appearance:**	white lyophilized powder
**Counter Ion:**	Trifluoroacetate
**Date of Mfg:**	03/04/20

Quality Assurance By: ______________________________
(Quality Control Department)

Date: 03/04/20

Zhejiang Ontores Biotechnologies Co.,Ltd

Address: NO.16 Road of Long Tan Hangzhou,Zhejiang , China, 311121
Email:support@ontores.com Fax: 0571-88863303 Tel: 0571-88607602 http://www.ontoresinc.com

S A M P L E   I N F O R M A T I O N

Sample Name:	PA1702	Acquired By:	System
Sample Type:	Unknown	Sample Set Name:	QC20200304
Vial:	25	Acq. Method Set:	15%_35% ACN 20min AB
Injection #:	1	Processing Method:	Agilent SB300 5um 250mm
Injection Volume:	10.00 ul	Channel Name:	220.0nm
Run Time:	20.0 Minutes	Proc. Chnl. Descr.:	PDA 220.0 nm
Date Acquired:	3/4/2020 10:29:03 AM CST		
Date Processed:	3/4/2020 10:59:14 AM CST		

Auto-Scaled Chromatogram

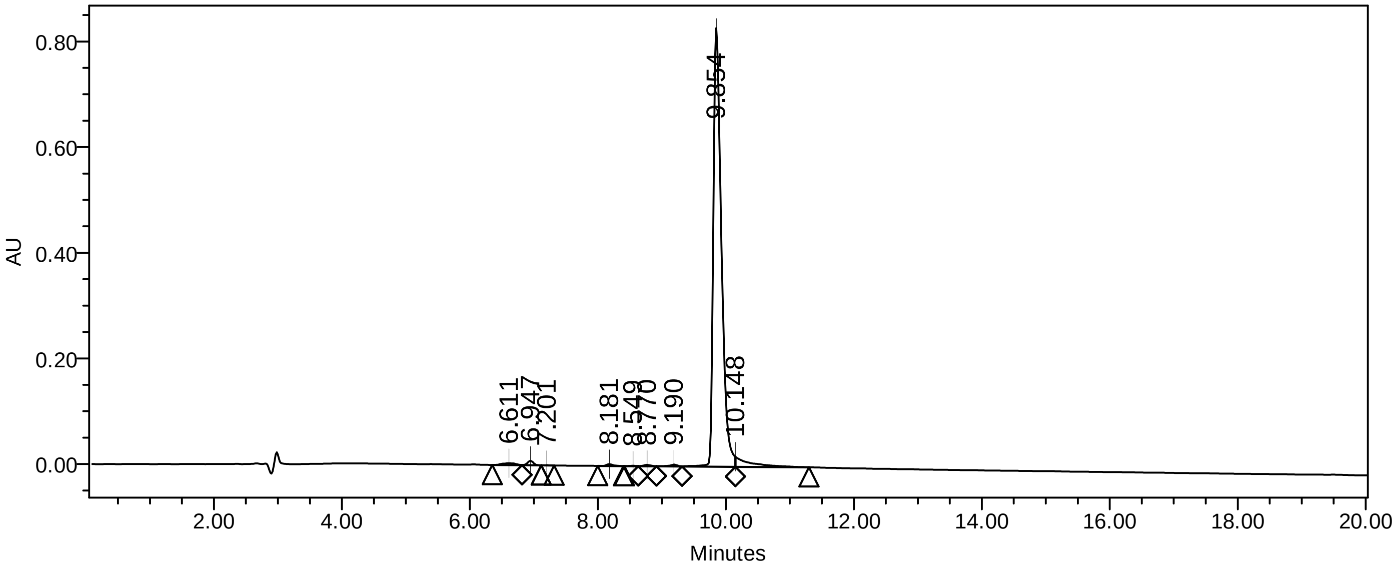

Peak Results

	Name	RT	Area	Height	% Area	Amount	Units
1		6.611	53411	3517	0.68		
2		6.947	53940	8397	0.69		
3		7.201	1879	309	0.02		
4		8.181	27056	3533	0.35		
5		8.549	6341	826	0.08		
6		8.770	23406	2748	0.30		
7		9.190	33555	3444	0.43		
8		9.854	7304372	832680	93.57		
9		10.148	302070	19039	3.87		

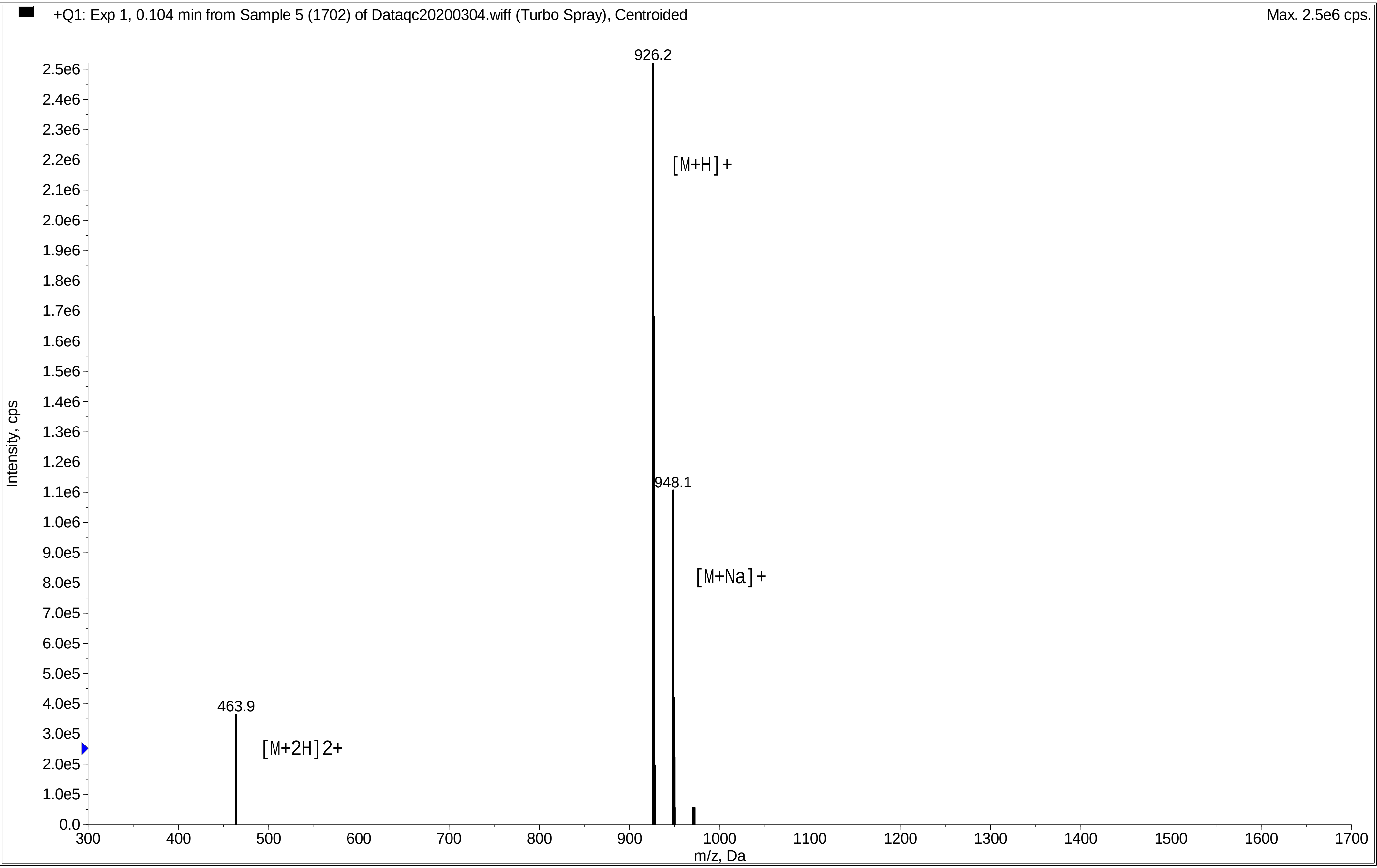

+Q1: Exp 1, 0.104 min from Sample 5 (1702) of Dataqc20200304.wiff (Turbo Spray), Centroided
Max. 2.5e6 cps.
926.2
[M+H]+
948.1
[M+Na]+
463.9
[M+2H]2+
Intensity, cps
m/z, Da

Quality Inspection Report

**Product Name:**	PA2155
**Lot Num:**	OP041720SF-031
**Sequence:**	H-D-Cys-D-Trp-D-Arg-OH
**Molecular Weight:**	462.55
**Mass Spectral Analysis:**	see attached MS spectrogram
**HPLC Analysis:**	Peptide purity：99.63%
**Column :**	agilent ZORBAX 300SB-C18 5um 4.6*250mm
**Mobile phase:**	A:0.1%TFA in 100%H2O<br>B:ACN
**Appearance:**	white lyophilized powder
**Counter Ion:**	Trifluoroacetate
**Date of Mfg:**	04/27/20

Quality Assurance By: ______________________________

(Quality Control Department)

Date: 04/27/20

Zhejiang Ontores Biotechnologies Co.,Ltd

Address: NO.16 Road of Long Tan Hangzhou,Zhejiang , China, 311121
Email:support@ontores.com Fax: 0571-88863303 Tel: 0571-88607602 http://www.ontoresinc.com

SAMPLE INFORMATION

Sample Name:	2155-1012	Acquired By:	System
Sample Type:	Unknown	Sample Set Name	QC20200426
Vial:	26	Acq. Method Set:	10_30%ACN in 20min CD
Injection #:	1	Processing Method	Agilent SB300 250mm 5um
Injection Volume:	20.00 ul	Channel Name:	220.0nm
Run Time:	20.0 Minutes	Proc. Chnl. Descr.:	PDA 220.0 nm
Date Acquired:	4/26/2020 13:25:40 PM CST		
Date Processed:	4/27/2020 8:55:16 AM CST		

Auto-Scaled Chromatogram

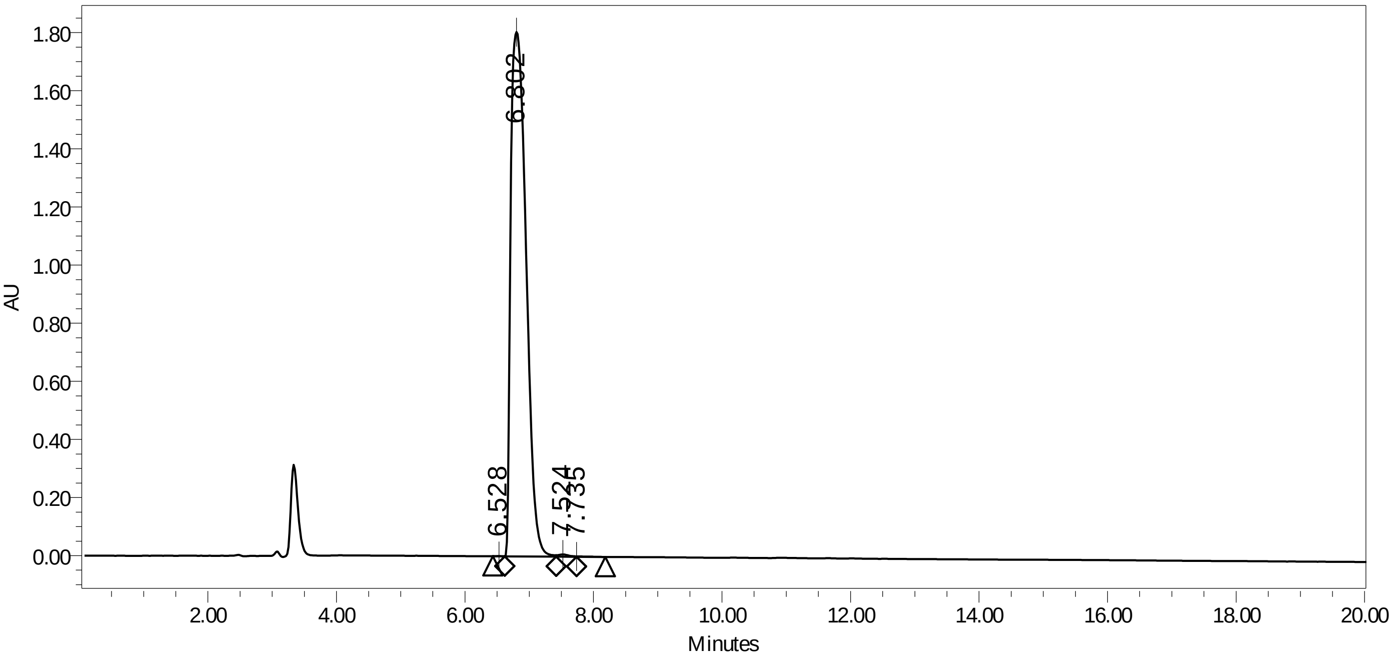

Peak Results

	Name	RT	Area	Height	% Area	Amount	Units
1		6.528	4648	695	0.02		
2		6.802	30004787	1805557	99.63		
3		7.524	90289	7745	0.30		
4		7.735	17573	1229	0.06		

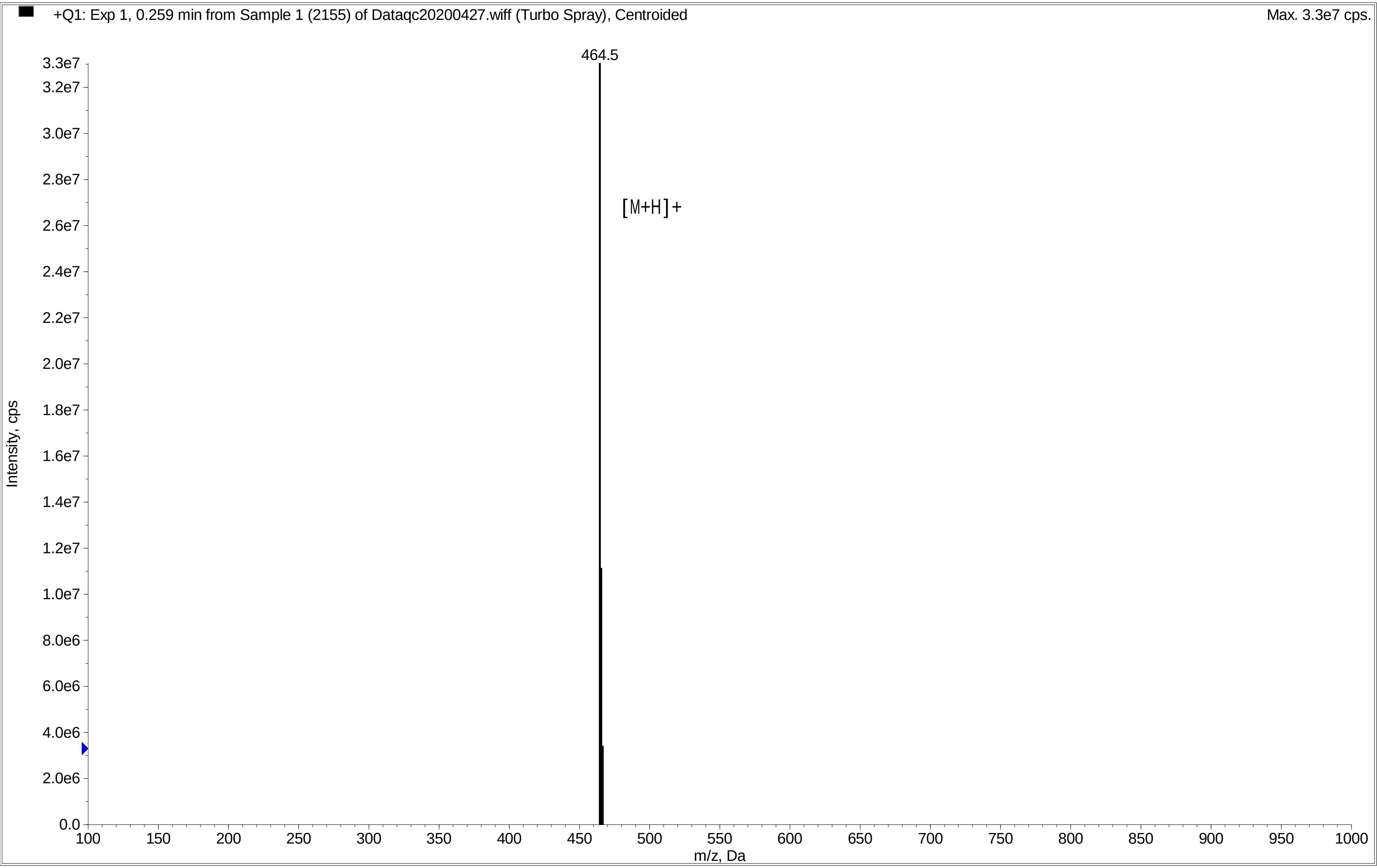

+Q1: Exp 1, 0.259 min from Sample 1 (2155) of Dataqc20200427.wiff (Turbo Spray), Centroided
Max. 3.3e7 cps.
464.5
[M+H]+
Intensity, cps
m/z, Da

ONTORES
BIOTECHNOLOGIES

Quality Inspection Report

**Product Name:**	PA2156
**Lot Num:**	OP041720SF-032
**Sequence:**	H-D-Arg-D-Trp-D-Cys-OH
**Molecular Weight:**	462.55
**Mass Spectral Analysis:**	see attached MS spectrogram
**HPLC Analysis:**	Peptide purity：96.75%
**Column :**	agilent ZORBAX 300SB-C18 5um 4.6*250mm
**Mobile phase:**	A:0.1%TFA in 100%H2O<br>B:ACN
**Appearance:**	white lyophilized powder
**Counter Ion:**	Trifluoroacetate
**Date of Mfg:**	04/27/20

Quality Assurance By: ______________________________
(Quality Control Department)

Date: 04/27/20

Zhejiang Ontores Biotechnologies Co.,Ltd

Address: NO.16 Road of Long Tan Hangzhou,Zhejiang , China, 311121
Email:support@ontores.com Fax: 0571-88863303 Tel: 0571-88607602 http://www.ontoresinc.com

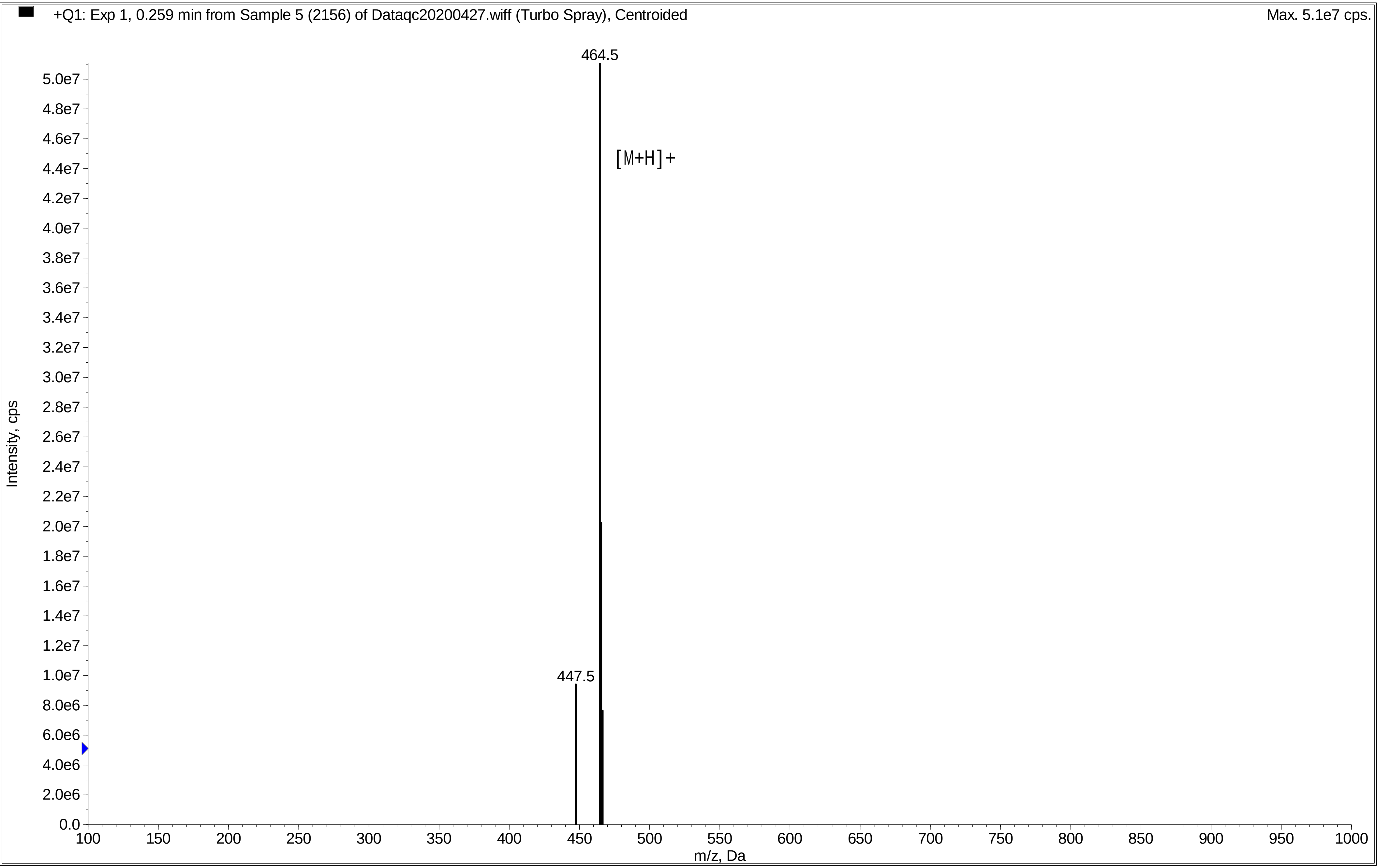

+Q1: Exp 1, 0.259 min from Sample 5 (2156) of Dataqc20200427.wiff (Turbo Spray), Centroided
Max. 5.1e7 cps.
464.5
[M+H]+
447.5
Intensity, cps
m/z, Da

Qc Reprot

SAMPLE INFORMATION

Sample Name:	PA2156 4-6	Acquired By:	System
Sample Type:	Unknown	Sample Set Name	QC20200426
Vial:	60	Acq. Method Set:	10_40%ACN in 20min CD
Injection #:	1	Processing Method	Agilent SB300 250mm 5um
Injection Volume:	20.00 ul	Channel Name:	220.0nm
Run Time:	20.0 Minutes	Proc. Chnl. Descr.:	PDA 220.0 nm
Date Acquired:	4/26/2020 12:30:13 PM CST		
Date Processed:	4/27/2020 8:56:59 AM CST		

Auto-Scaled Chromatogram

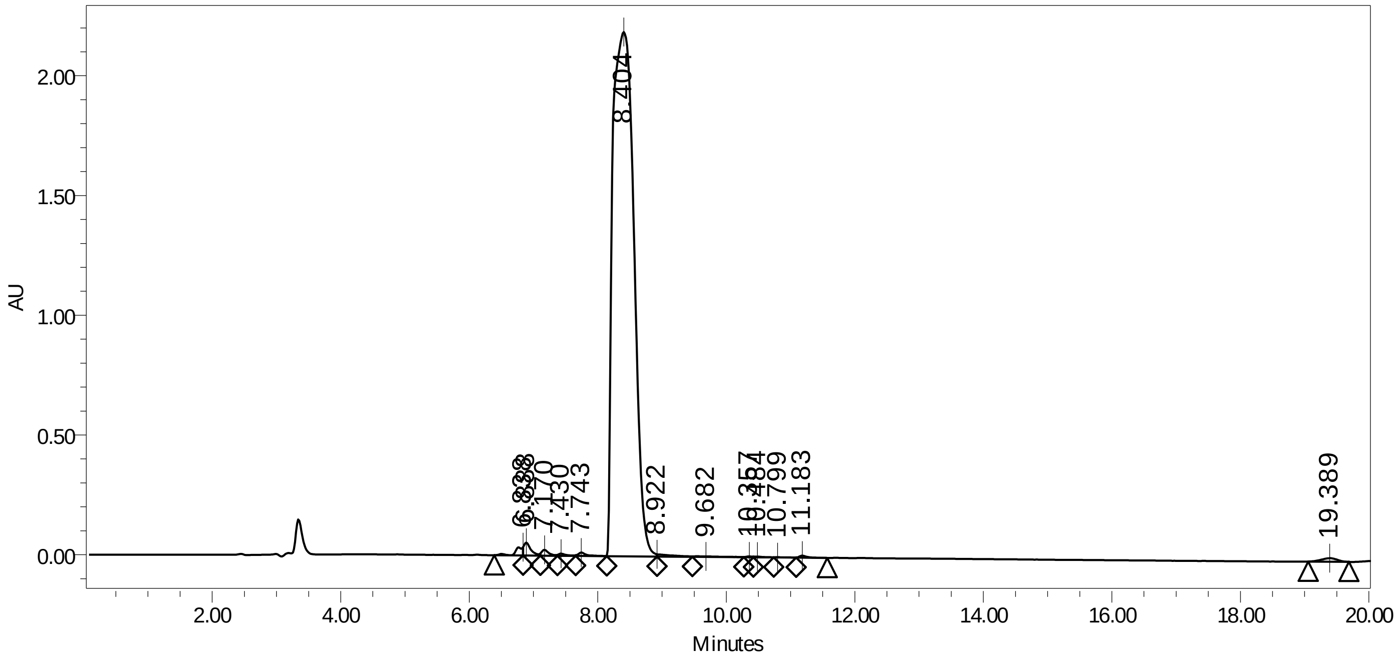

Peak Results

	Name	RT	Area	Height	% Area	Amount	Units
1		6.838	273732	34494	0.53		
2		6.888	405496	53439	0.79		
3		7.170	184527	24300	0.36		
4		7.430	75592	9097	0.15		
5		7.743	123605	14371	0.24		
6		8.404	49888088	2188833	96.75		
7		8.922	152947	9508	0.30		
8		9.682	72985	2399	0.14		
9		10.357	23904	3559	0.05		
10		10.484	31669	3201	0.06		
11		10.799	14213	1239	0.03		
12		11.183	68798	8433	0.13		
13		19.389	250413	14665	0.49		

丹港® 生物科技
DGpeptidesCo.,Ltd

CERTIFICATE OF ANALYSIS

**Product Name**	S-4842
**Lot NO.**	DG-92211
**Order ID**	DG#S20210303760
**Sequence**	Mal-WR
**Length**	2AA
**Modification**	N/A
**Molecular Weight (MW)**	511.55
**Storage**	≤-20℃

**Test Items**	**Specifications**	**Results**
**Purity by HPLC**	>90%	94.85%
**Weight**	5mg（2.5mg*2）	5mg（2.5mg*2）
**Peptide Content**	N/A	N/A
**Moisture content**	N/A	N/A
**Acetic acid content**	N/A	N/A
**Appearance**	White to off-white lyophilized powde	Conforms

Certified by:Quality Assurance Department

Date: 03/30/2021

Note: this product is intended for research use only; not for diagnostic or human use.

DGpeptides Co., Ltd

Address: FeiJiaTang Road 588, Downtown,Hangzhou city, Zhejiang province,China.

Tel：400-6153-668   http://www.dgpeptides.com

HPLC

Sample:	S-4842	Analyzed date:	2021-03-29
Sequence:	Mal-WR		
Lot. No.:	DG-92211		
Column:	4.6×250mm,Sinochrom ODS-BP 5μm		
Solvent A	A: 0.1% Trifluoroacetic Acid in 100% Acetonitrile		
Solvent B	B: 0.1% Trifluoroacetic Acid in 100% Water		

Gradient:

	A	B
0.0min	20%	80%
25.0min	45%	55%
25.1min	100%	0%
30.0min	Stop	

Volume: 5μl

Wavelength: 220nm

Flow rate: 1.0ml/min

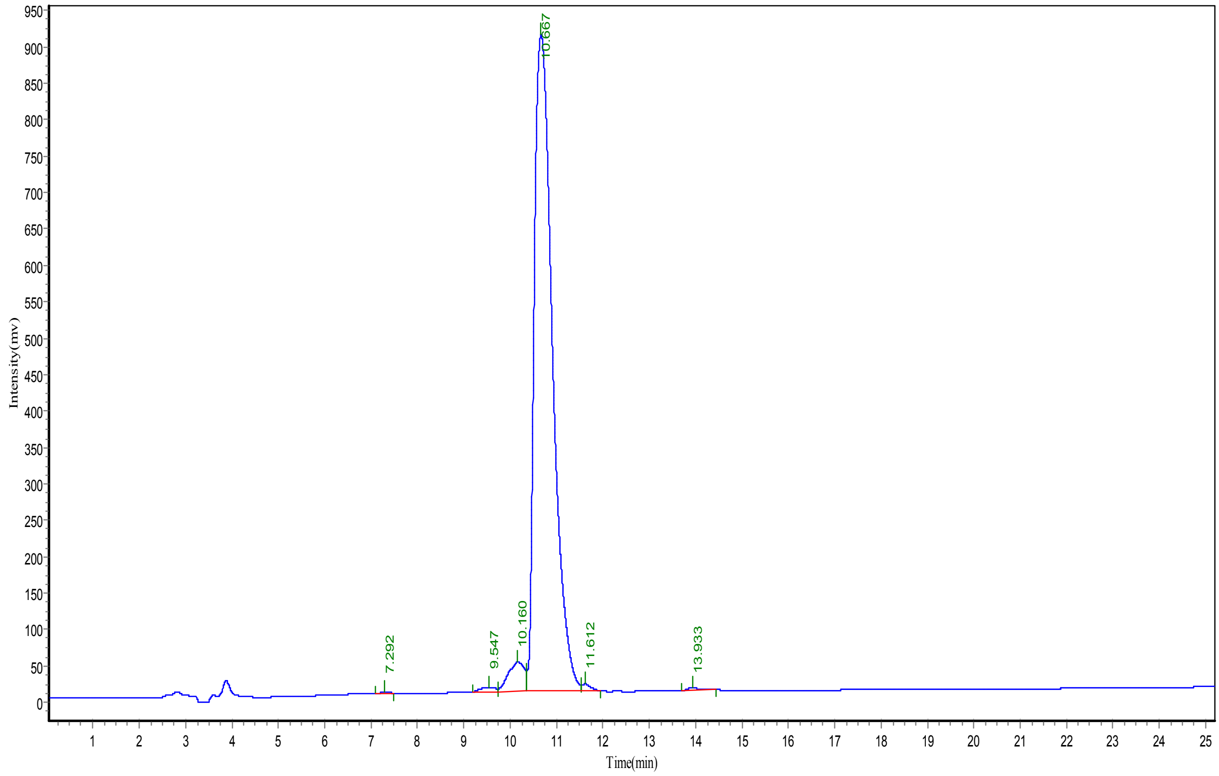

Peak	Time	Height	Area	Conc.
1	7.292	1975.566	23252.490	0.0899
2	9.547	5477.303	127220.445	0.4918
3	10.160	40193.691	976375.000	3.7744
4	10.667	901721.438	24536436.000	94.8524
5	11.612	9176.358	125763.781	0.4862
6	13.933	3450.288	78963.820	0.3053
Total				100.000

MASS

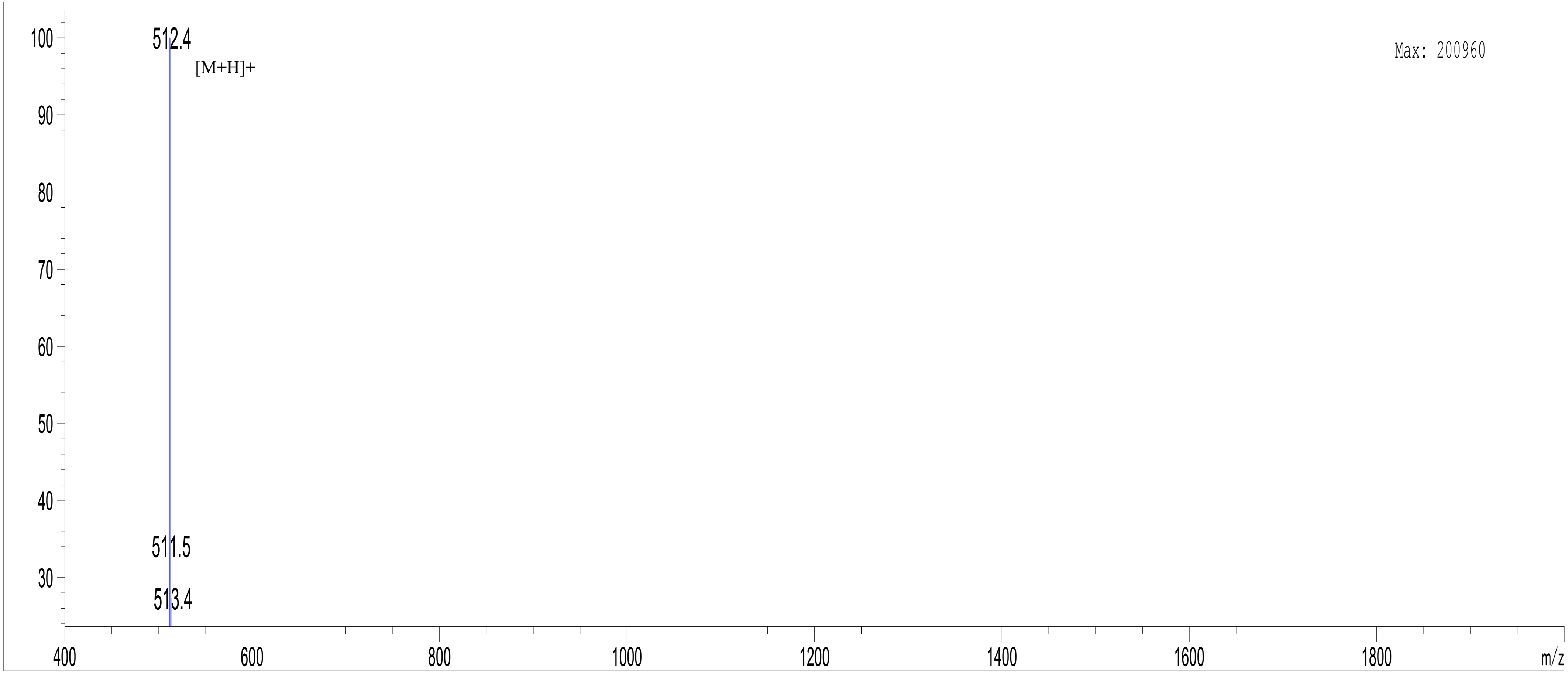

512.4
[M+H]+
511.5
513.4
Max: 200960
100
90
80
70
60
50
40
30
400
600
800
1000
1200
1400
1600
1800
m/z

CERTIFICATE OF ANALYSIS

**Product Name**	S-5491
**Lot NO.**	DG-93444
**Order ID**	DG#S20210423797
**Sequence**	Ac-RWR-NH2
**Length**	3AA
**Modification**	N/A
**Molecular Weight (MW)**	557.64
**Storage**	≤-20℃

**Test Items**	**Specifications**	**Results**
**Purity by HPLC**	>90%	96.51%
**Weight**	5mg（2.5mg*2）	5mg（2.5mg*2）
**Peptide Content**	N/A	N/A
**Moisture content**	N/A	N/A
**Acetic acid content**	N/A	N/A
**Appearance**	White to off-white lyophilized powde	Conforms

Certified by:Quality Assurance Department

Date: 05/17/2021

Note: this product is intended for research use only; not for diagnostic or human use.

DGpeptides Co., Ltd

Address: FeiJiaTang Road 588, Downtown,Hangzhou city, Zhejiang province,China.

Tel：400-6153-668 http://www.dgpeptides.com

HPLC

Sample: S-5491 Analyzed date: 2021-05-13
Sequence: Ac-RWR-NH2

Lot. No.: DG-93444
Column: Symmetrix ODS-R, 4.6*250mm, 5µm
Solvent A A: 0.1% Trifluoroacetic Acid in 100% Acetonitrile
Solvent B B: 0.1% Trifluoroacetic Acid in 100% Water

	A	B
0.0min	8%	92%
25.0min	33%	67%
25.1min	100%	0%
30.0min	Stop	

Volume: 20µl
Wavelength: 220nm
Flow rate: 1.0ml/min

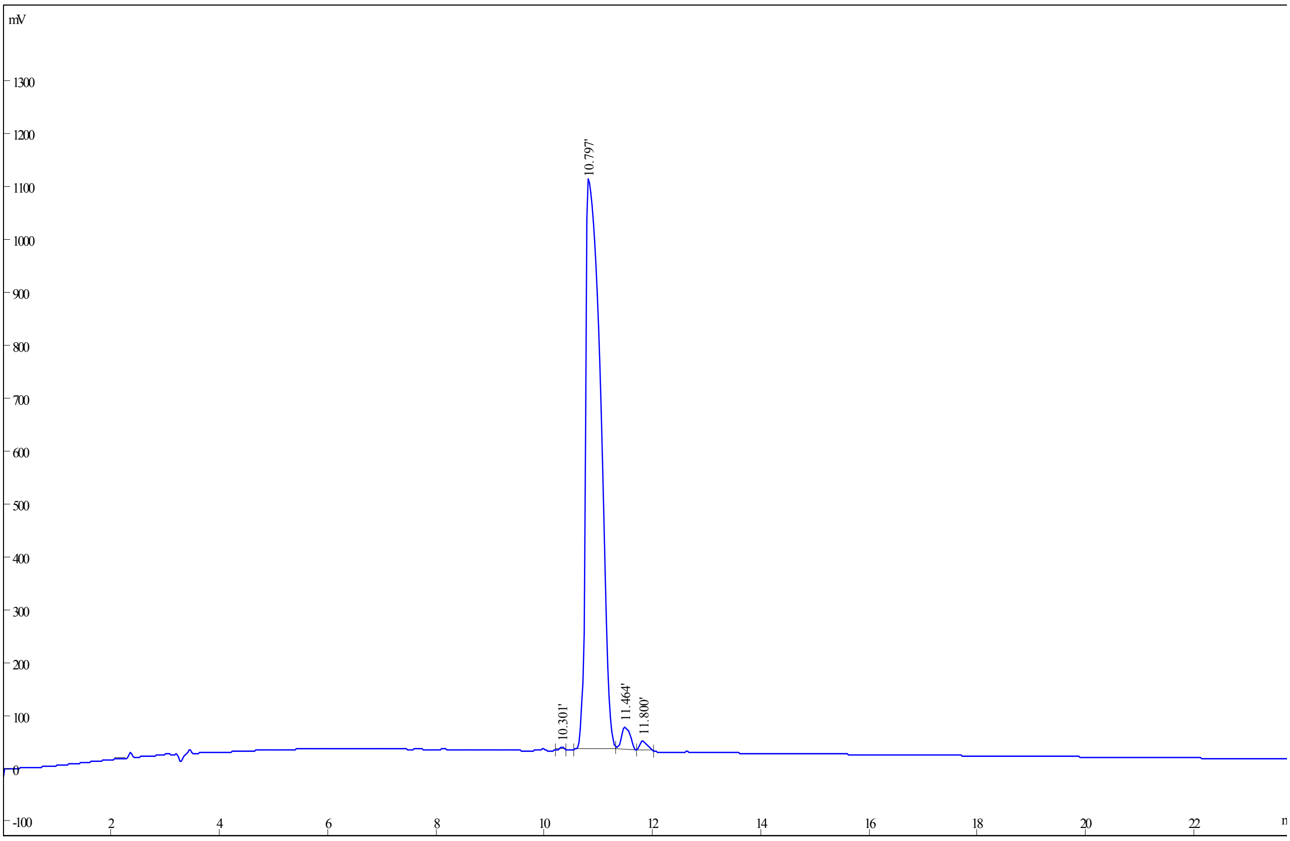

Rank	Time	Conc.	Area	Height
1	10.301	0.1307	27823	5387
2	10.797	96.51	20541898	1080767
3	11.464	2.452	521895	45649
4	11.800	0.9137	194490	18931
Total		100	21286106	1150734

MASS

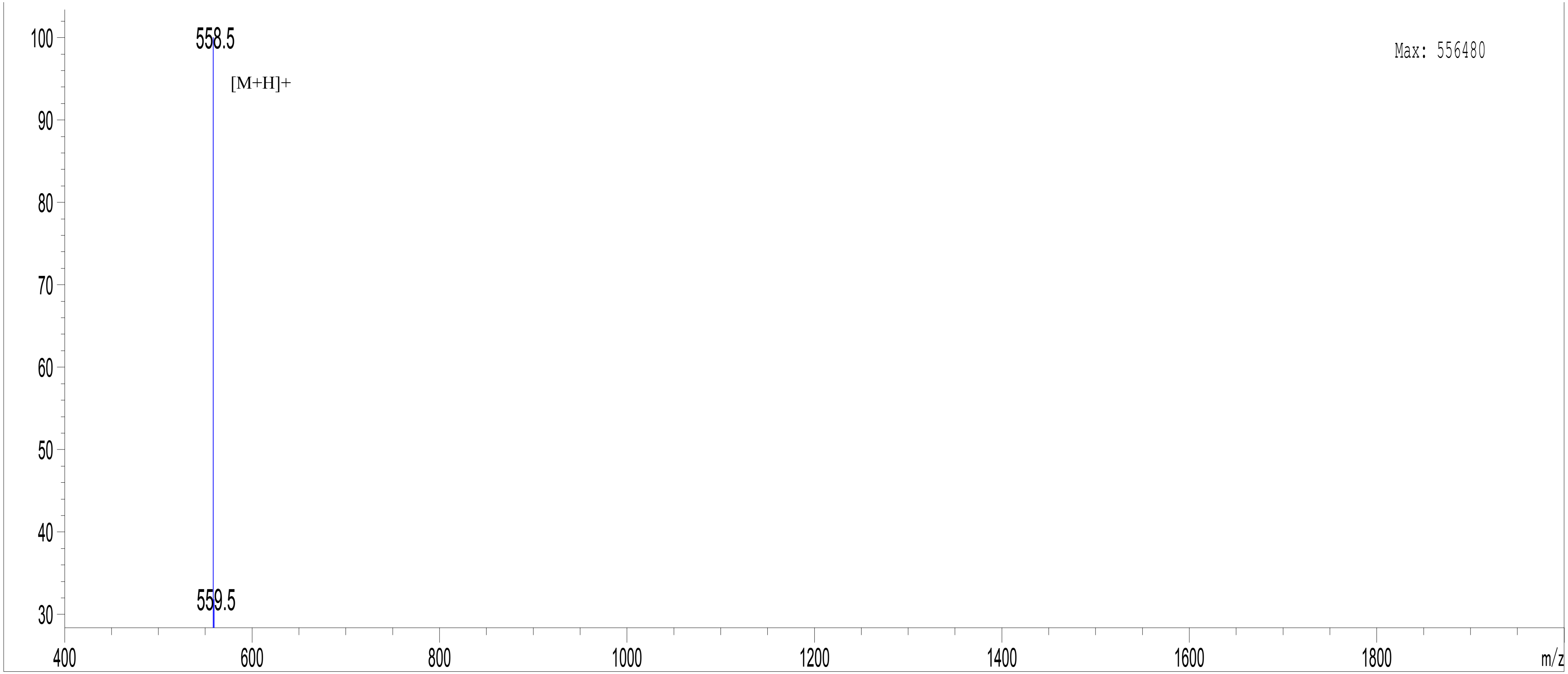

558.5
[M+H]+
559.5
Max: 556480
100
90
80
70
60
50
40
30
400
600
800
1000
1200
1400
1600
1800
m/z

丹港®生物科技
DGpeptidesCo.,Ltd

CERTIFICATE OF ANALYSIS

**Product Name**	S-5535
**Lot NO.**	DG-96743
**Order ID**	DG#S20210628822
**Sequence**	Abu-WR
**Length**	3AA
**Modification**	N/A
**Molecular Weight (MW)**	445.53
**Storage**	≤-20℃

**Test Items**	**Specifications**	**Results**
**Purity by HPLC**	>90%	95.03%
**Weight**	5mg（2.5mg*2）	5mg（2.5mg*2）
**Peptide Content**	N/A	N/A
**Moisture content**	N/A	N/A
**Acetic acid content**	N/A	N/A
**Appearance**	White to off-white lyophilized powde	Conforms

Certified by:Quality Assurance Department

Date: 07/06/2021

Note: this product is intended for research use only; not for diagnostic or human use.

DGpeptides Co., Ltd

Address: FeiJiaTang Road 588, Downtown,Hangzhou city, Zhejiang province,China.

Tel：400-6153-668 http://www.dgpeptides.com

HPLC

Sample:	S-5535	Analyzed date:	2021-07-02
Sequence:	Abu-WR		
Lot. No.:	DG-96743		
Column:	4.6×250mm,Sinochrom ODS-BP 5μm		
Solvent A	A: 0.1% Trifluoroacetic Acid in 100% Acetonitrile		
Solvent B	B: 0.1% Trifluoroacetic Acid in 100% Water		

Gradient:

	A	B
0.0min	12%	88%
25.0min	37%	63%
25.1min	100%	0%
30.0min	Stop	

Volume: 5μl

Wavelength: 220nm

Flow rate: 1.0ml/min

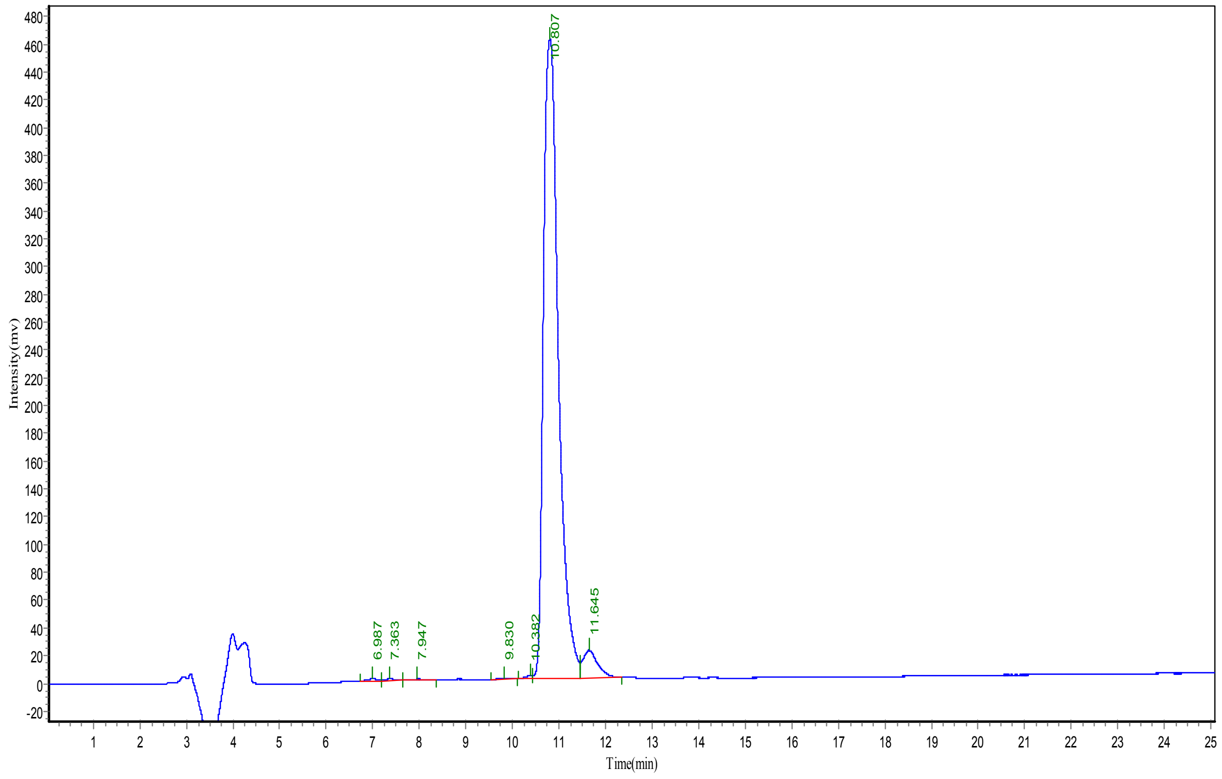

Peak	Time	Height	Area	Conc.
1	6.987	1552.113	25219.510	0.2327
2	7.363	1281.179	19965.799	0.1843
3	7.947	832.685	18263.203	0.1685
4	9.830	711.628	12630.402	0.1166
5	10.382	2286.322	22126.855	0.2042
6	10.807	460361.125	10297321.000	95.0307
7	11.645	19908.828	440254.750	4.0630
Total				100.000

MASS

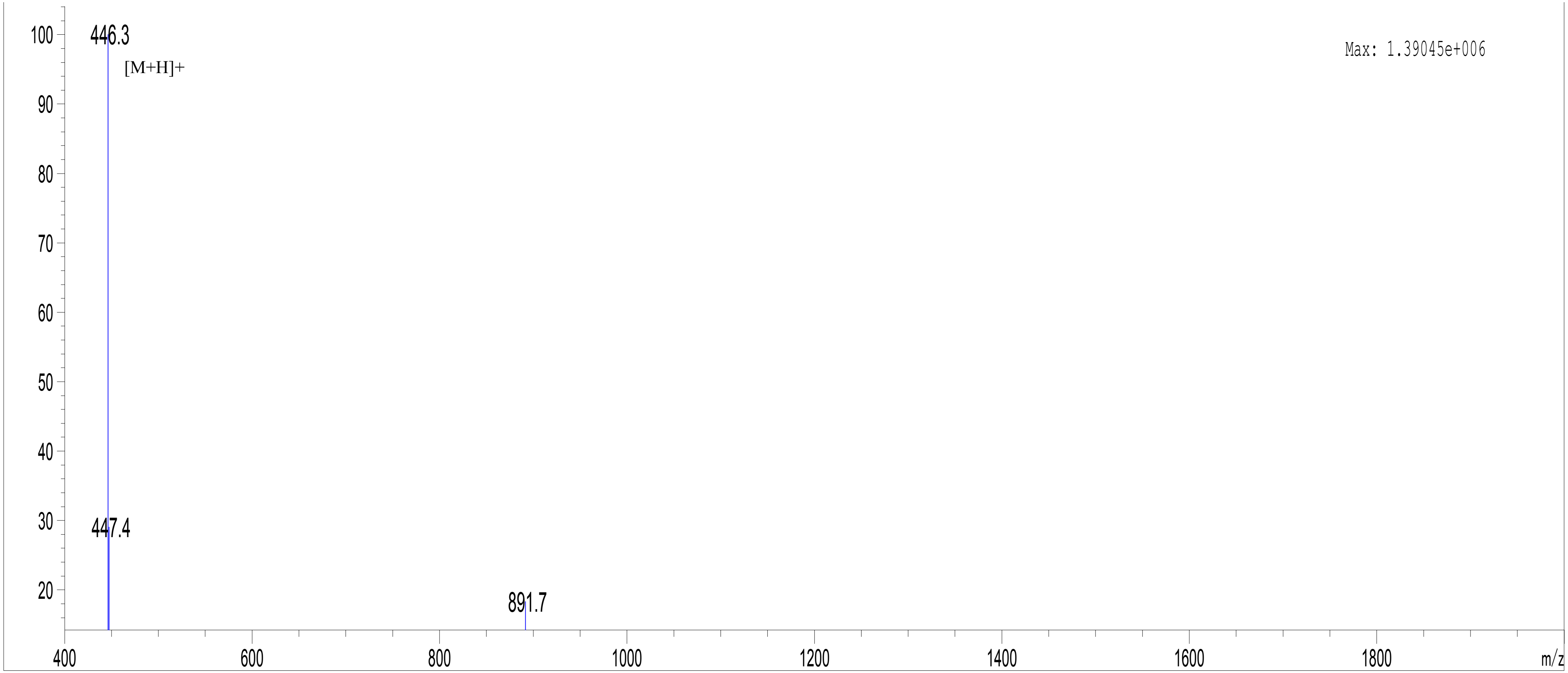

446.3
[M+H]+
447.4
891.7
Max: 1.39045e+006
100
90
80
70
60
50
40
30
20
400
600
800
1000
1200
1400
1600
1800
m/z

丹港®生物科技
DGpeptidesCo.,Ltd

CERTIFICATE OF ANALYSIS

**Product Name**	S-5543
**Lot NO.**	DG-96805
**Order ID**	DG#S20210712831
**Sequence**	Ac-CWR
**Length**	3AA
**Modification**	N/A
**Molecular Weight (MW)**	505.59
**Storage**	≤-20℃

**Test Items**	**Specifications**	**Results**
**Purity by HPLC**	>90%	95.14%
**Weight**	5mg（2.5mg*2）	5mg（2.5mg*2）
**Peptide Content**	N/A	N/A
**Moisture content**	N/A	N/A
**Acetic acid content**	N/A	N/A
**Appearance**	White to off-white lyophilized powde	Conforms

Certified by:Quality Assurance Department

Date: 07/23/2021

Note: this product is intended for research use only; not for diagnostic or human use.

DGpeptides Co., Ltd

Address: FeiJiaTang Road 588, Downtown,Hangzhou city, Zhejiang province,China.

Tel：400-6153-668   http://www.dgpeptides.com

HPLC

Sample: S-5543 Analyzed date: 2021-07-21
Sequence: Ac-CWR
Lot. No.: DG-96805
Column: 4.6×250mm,Sinochrom ODS-BP 5μm
Solvent A A: 0.1% Trifluoroacetic Acid in 100% Acetonitrile
Solvent B B: 0.1% Trifluoroacetic Acid in 100% Water
Gradient:

	A	B
0.0min	16%	84%
25.0min	41%	59%
25.1min	100%	0%
30.0min	Stop	

Volume: 5μl
Wavelength: 220nm
Flow rate: 1.0ml/min

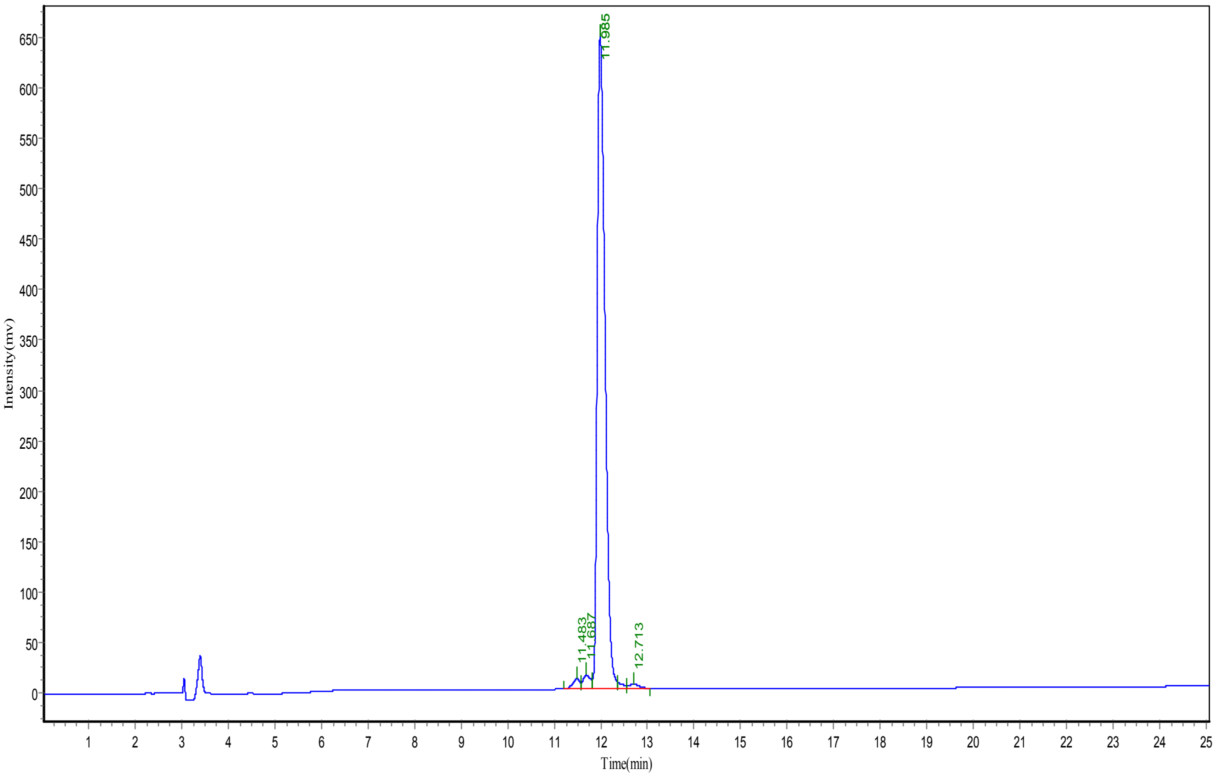

Peak	Time	Height	Area	Conc.
1	11.483	10436.898	103925.859	1.3185
2	11.687	13909.161	156132.625	1.9808
3	11.985	646229.438	7499330.500	95.1406
4	12.316	6477.459	52070.277	0.6606
5	12.713	4584.422	70906.195	0.8996
Total				100.000

MASS

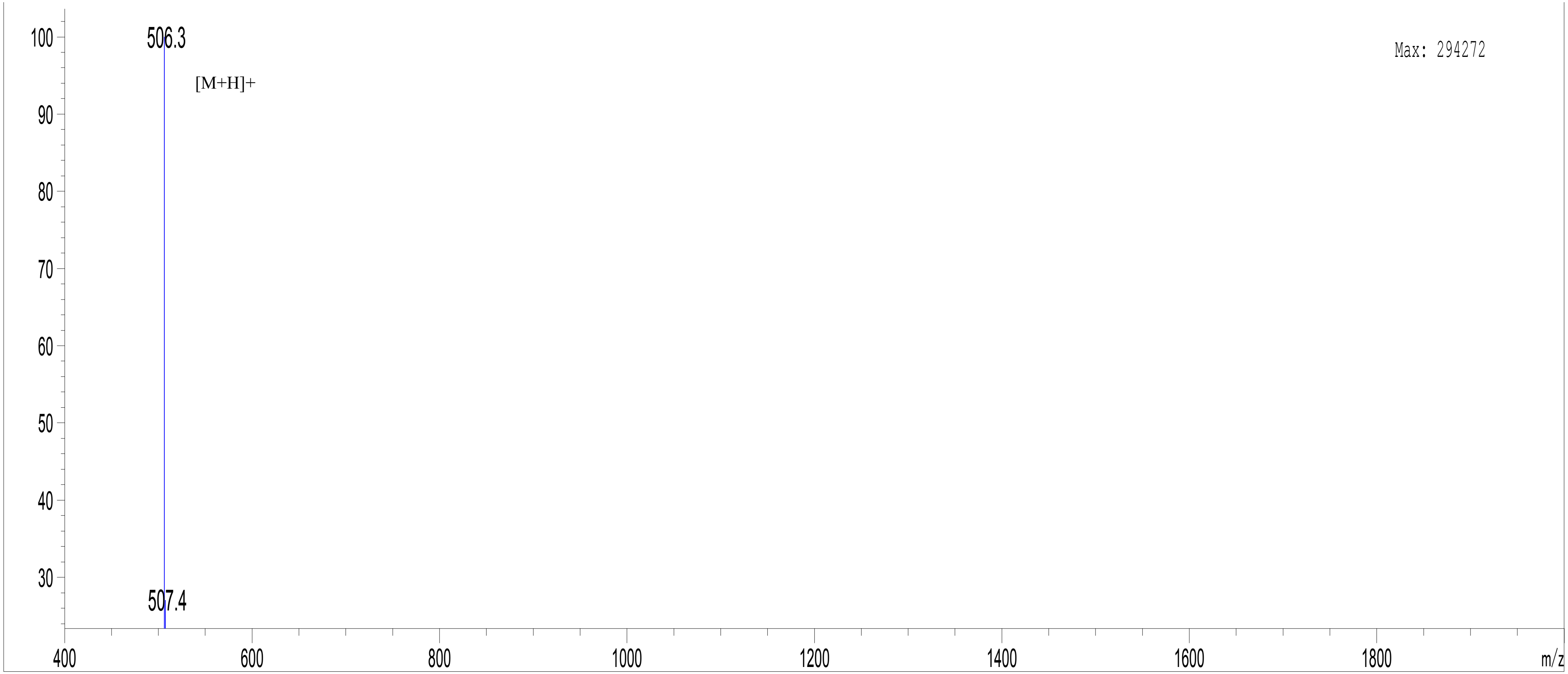

506.3
[M+H]+
Max: 294272
507.4
100
90
80
70
60
50
40
30
400
600
800
1000
1200
1400
1600
1800
m/z

丹港® 生物科技
DGpeptidesCo.,Ltd

CERTIFICATE OF ANALYSIS

**Product Name**	S-5544
**Lot NO.**	DG-96806
**Order ID**	DG#S20210712831
**Sequence**	CWR-NH2
**Length**	3AA
**Modification**	N/A
**Molecular Weight (MW)**	462.56
**Storage**	≤-20℃

**Test Items**	**Specifications**	**Results**
**Purity by HPLC**	>90%	95.61%
**Weight**	5mg（2.5mg*2）	5mg（2.5mg*2）
**Peptide Content**	N/A	N/A
**Moisture content**	N/A	N/A
**Acetic acid content**	N/A	N/A
**Appearance**	White to off-white lyophilized powde	Conforms

Certified by:Quality Assurance Department

Date: 07/23/2021

Note: this product is intended for research use only; not for diagnostic or human use.

HPLC

Sample:	S-5544	Analyzed date:	2021-07-21
Sequence:	CWR-NH2		

Lot. No.: DG-96806

Column: Symmetrix ODS-R, 4.6*250mm, 5µm

Solvent A A: 0.1% Trifluoroacetic Acid in 100% Acetonitrile

Solvent B B: 0.1% Trifluoroacetic Acid in 100% Water

	A	B
0.0min	6%	94%
25.0min	31%	69%
25.1min	100%	0%
30.0min	Stop	

Volume: 20µl

Wavelength: 220nm

Flow rate: 1.0ml/min

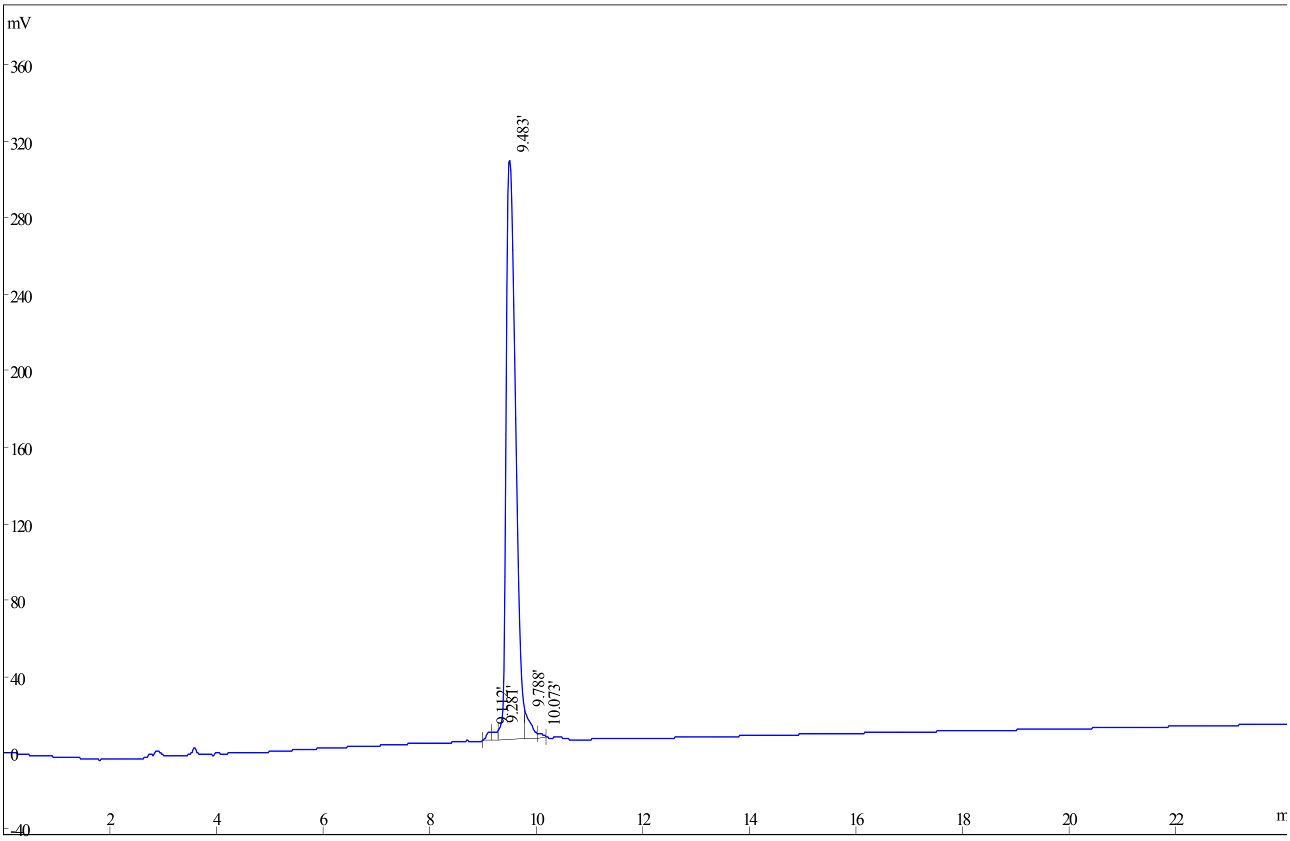

Rank	Time	Conc.	Area	Height
1	9.112	0.7145	25748	4023
2	9.281	0.8722	31433	4570
3	9.483	95.61	3445715	302253
4	9.788	2.427	87473	12298
5	10.073	0.3737	13467	1947
Total		100	3603836	325091

MASS

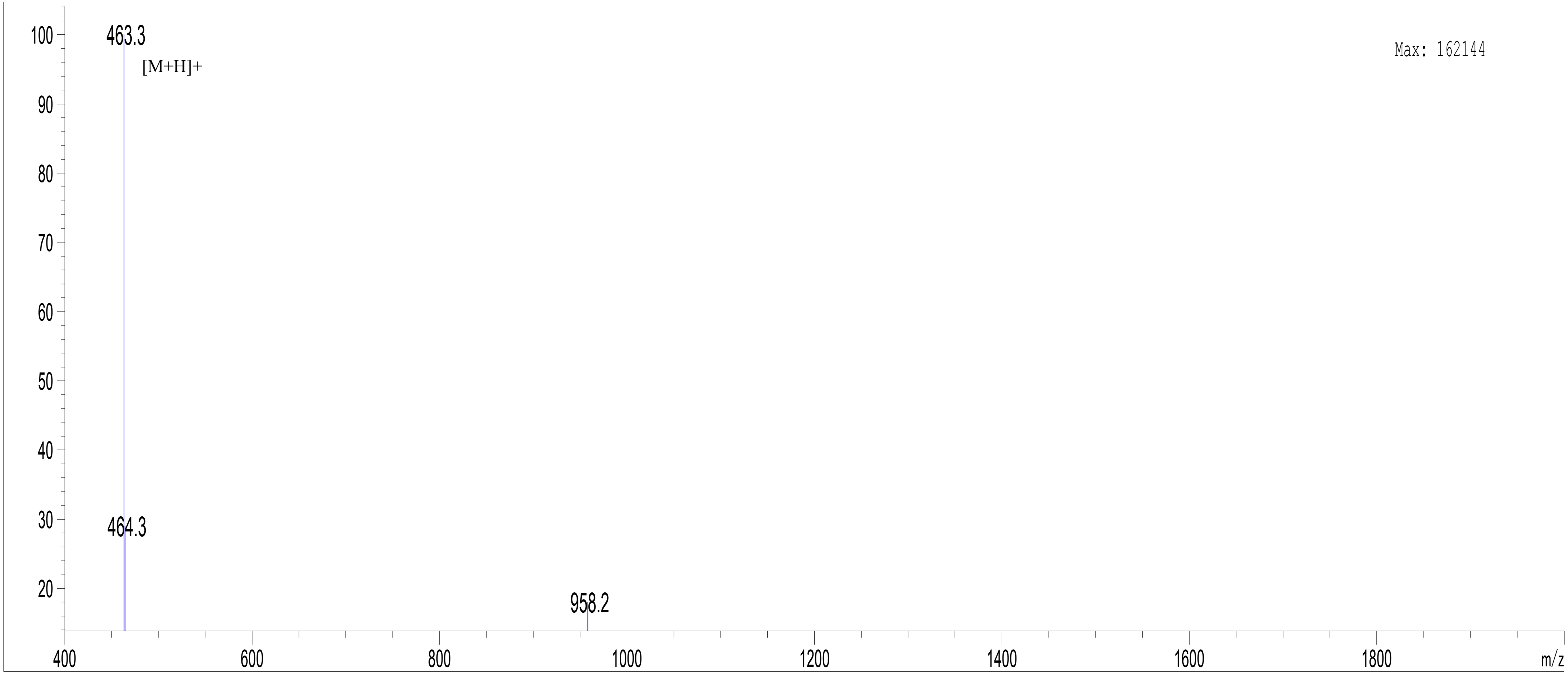

463.3
[M+H]+
Max: 162144
464.3
958.2
100
90
80
70
60
50
40
30
20
400
600
800
1000
1200
1400
1600
1800
m/z

ONTORES
BIOTECHNOLOGIES

Quality Inspection Report

**Product Name:**	PA3175
**Lot Num:**	OP1204020SF-004
**Sequence:**	CWR，L 型氨基酸
**Molecular Weight:**	463.55
**Mass Spectral Analysis:**	see attached MS spectrogram
**HPLC Analysis:**	Peptide purity：97.59%
**Column :**	agilent ZORBAX 300SB-C18 5um 4.6*250mm
**Mobile phase:**	A:0.1%TFA in 100%H2O<br>B:ACN
**Appearance:**	white lyophilized powder
**Counter Ion:**	Trifluoroacetate
**Date of Mfg:**	12/15/20

浙江昂拓莱司生物技术有限公司
质检专用章

Quality Assurance By: ____________________
(Quality Control Department)

Date: 12/15/20

Zhejiang Ontores Biotechnologies Co.,Ltd

Address: NO.16 Road of Long Tan Hangzhou,Zhejiang , China, 311121
Email:support@ontores.com Fax: 0571-88863303 Tel: 0571-88607602 http://www.ontoresinc.com

Qc Report

SAMPLE INFORMATION

Sample Name:	PA3175	Acquired By:	System
Sample Type:	Unknown	Sample Set Name:	QC20201215B
Vial:	25	Acq. Method Set:	6%_26% ACN 20min AB
Injection #:	1	Processing Method:	Agilent SB300 5um 250mm1
Injection Volume:	5.00 ul	Channel Name:	220.0nm
Run Time:	20.0 Minutes	Proc. Chnl. Descr.:	PDA 220.0 nm
Date Acquired:	12/15/2020 12:19:41 PM CST		
Date Processed:	12/15/2020 12:40:49 PM CST		

Auto-Scaled Chromatogram

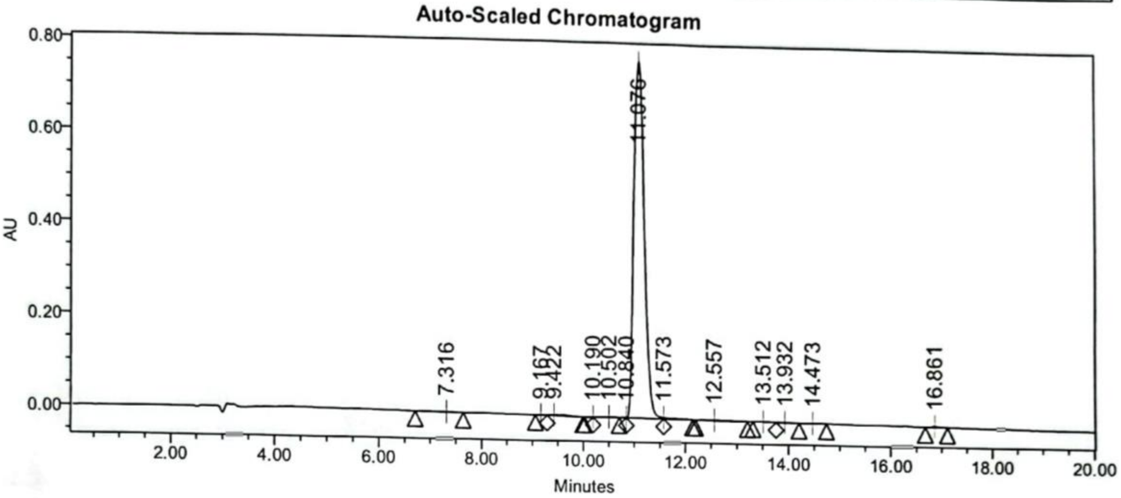

Peak Results

	Name	RT	Area	Height	% Area	Amount	Units
1		7.316	32874	1039	0.29		
2		9.167	6954	662	0.06		
3		9.422	54969	2190	0.49		
4		10.190	1844	320	0.02		
5		10.502	17711	1136	0.16		
6		10.840	2345	638	0.02		
7		11.076	11058083	775613	97.59		
8		11.573	49200	2747	0.43		
9		12.557	39274	1003	0.35		
10		13.512	14440	854	0.13		
11		13.932	13315	889	0.12		

Peak Results

	Name	RT	Area	Height	% Area	Amount	Units
12		14.473	13429	818	0.12		
13		16.861	27130	2613	0.24		

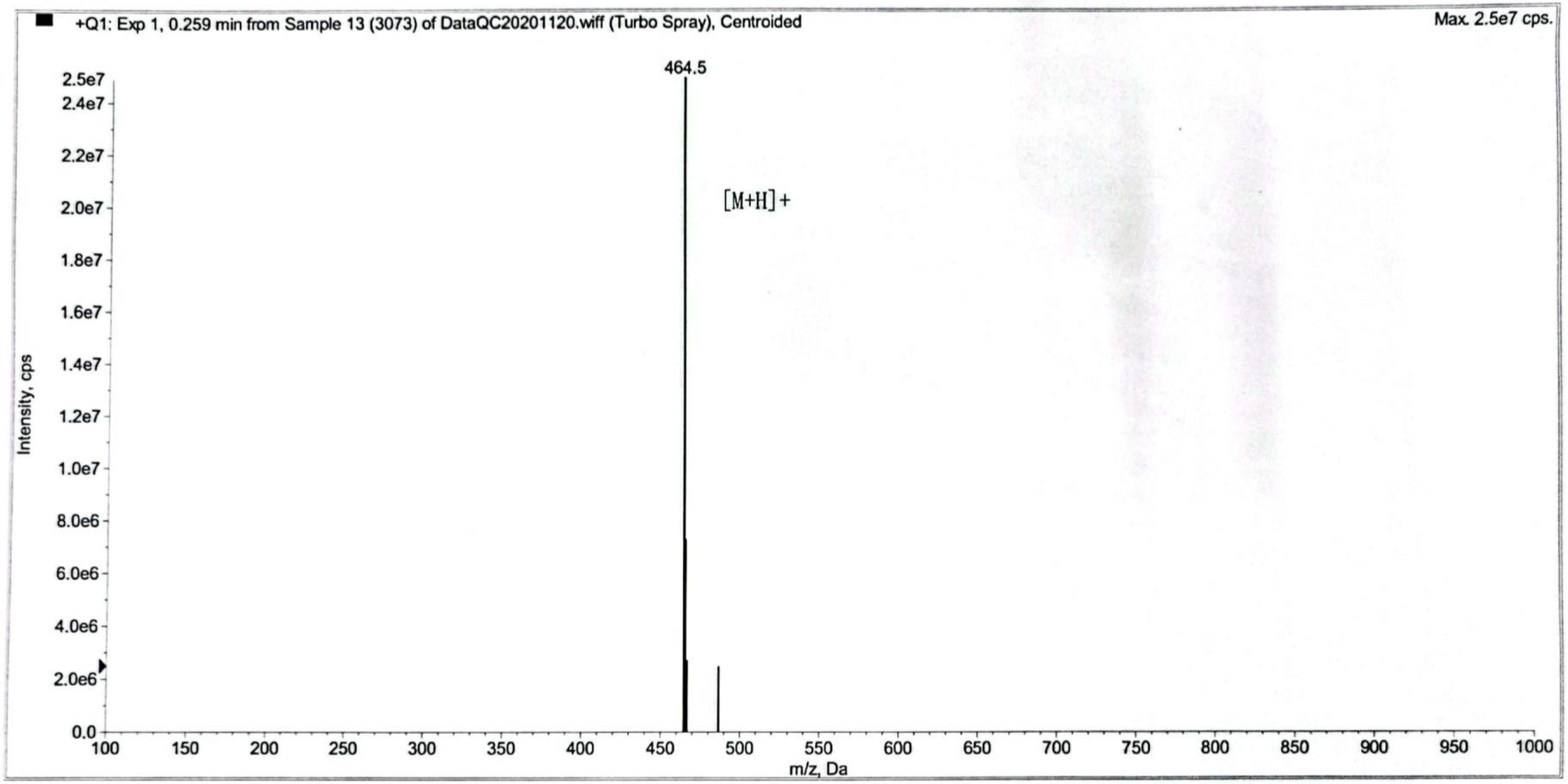
+Q1: Exp 1, 0.259 min from Sample 13 (3073) of DataQC20201120.wiff (Turbo Spray), Centroided
Max. 2.5e7 cps.
464.5
[M+H]+
Intensity, cps
2.5e7
2.4e7
2.2e7
2.0e7
1.8e7
1.6e7
1.4e7
1.2e7
1.0e7
8.0e6
6.0e6
4.0e6
2.0e6
0.0
100
150
200
250
300
350
400
450
500
550
600
650
700
750
800
850
900
950
1000
m/z, Da

ONTORES
BIOTECHNOLOGIES

Quality Inspection Report

**Product Name:**	PA3174
**Lot Num:**	OP1204020SF-003
**Sequence:**	SWR，L 型氨基酸
**Molecular Weight:**	447.5
**Mass Spectral Analysis:**	see attached MS spectrogram
**HPLC Analysis:**	Peptide purity：94.81%
**Column :**	agilent ZORBAX 300SB-C18 5um 4.6*250mm
**Mobile phase:**	A:0.1%TFA in 100%H2O<br>B:ACN
**Appearance:**	white lyophilized powder
**Counter Ion:**	Trifluoroacetate
**Date of Mfg:**	12/14/20

浙江昂拓莱司生物技术有限公司 质检专用章

Quality Assurance By: ____________________
(Quality Control Department)

Date: 12/14/20

Zhejiang Ontores Biotechnologies Co.,Ltd

Address: NO.16 Road of Long Tan Hangzhou,Zhejiang , China, 311121
Email:support@ontores.com Fax: 0571-88863303 Tel: 0571-88607602 http://www.ontoresinc.com

Empower2 software

Qc Report

SAMPLE INFORMATION

Sample Name:	3174-414	Acquired By:	System
Sample Type:	Unknown	Sample Set Name:	QC20201211B
Vial:	56	Acq. Method Set:	6%_26% ACN 20min AB
Injection #:	1	Processing Method:	Agilent SB300 5um 250mm1
Injection Volume:	1.00 ul	Channel Name:	214.0nm
Run Time:	20.0 Minutes	Proc. Chnl. Descr.:	PDA 214.0 nm
Date Acquired:	12/11/2020 4:01:20 PM CST		
Date Processed:	12/14/2020 9:23:10 AM CST		

Auto-Scaled Chromatogram

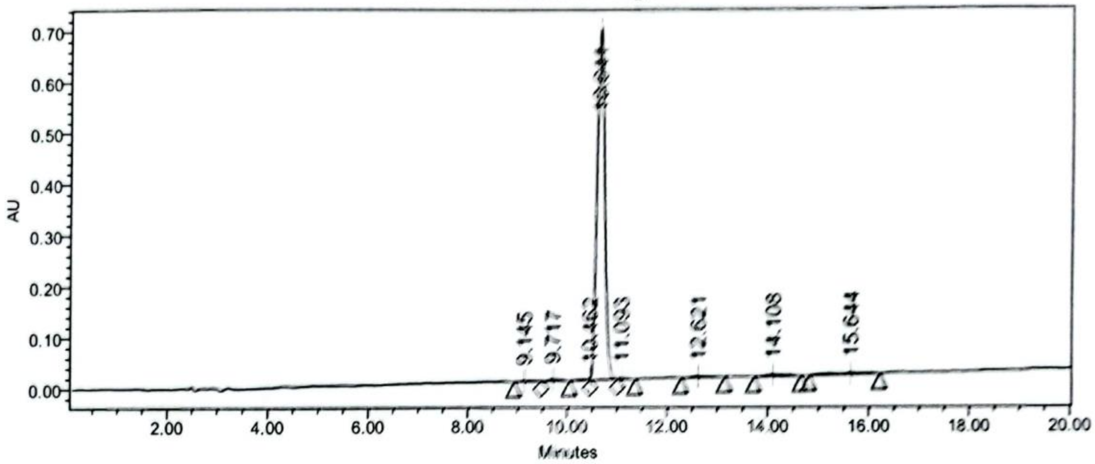

Peak Results

	Name	RT	Area	Height	% Area	Amount	Units
1		9.145	34190	1401	0.44		
2		9.717	55939	3395	0.71		
3		10.462	25531	2371	0.33		
4		10.641	7422789	690054	94.81		
5		11.093	29226	2227	0.37		
6		12.621	38172	1903	0.49		
7		14.108	99318	3425	1.27		
8		15.644	123943	2676	1.58		

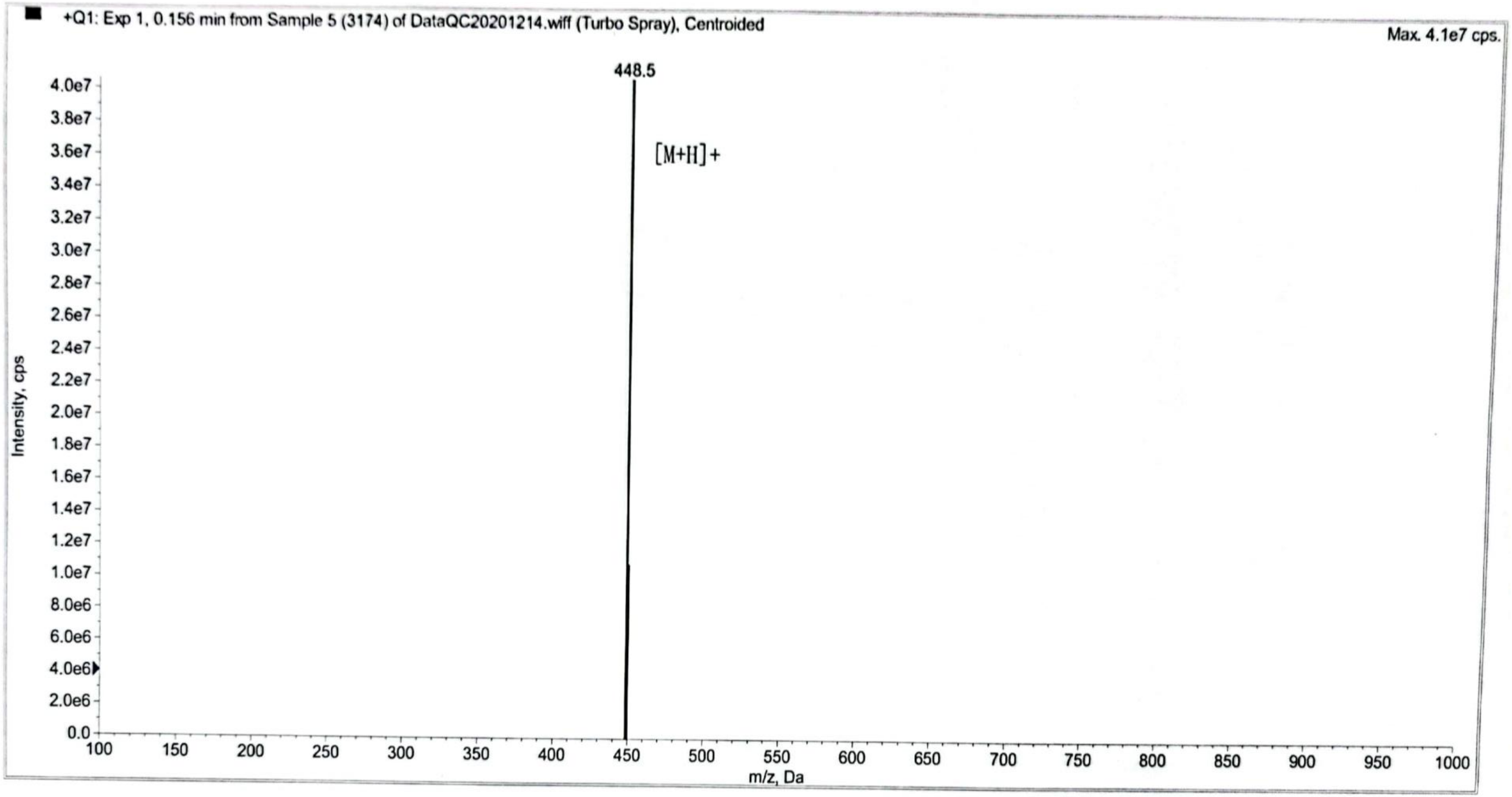

+Q1: Exp 1, 0.156 min from Sample 5 (3174) of DataQC20201214.wiff (Turbo Spray), Centroided
Max. 4.1e7 cps.
448.5
[M+H]+
Intensity, cps
4.0e7
3.8e7
3.6e7
3.4e7
3.2e7
3.0e7
2.8e7
2.6e7
2.4e7
2.2e7
2.0e7
1.8e7
1.6e7
1.4e7
1.2e7
1.0e7
8.0e6
6.0e6
4.0e6
2.0e6
0.0
100
150
200
250
300
350
400
450
500
550
600
650
700
750
800
850
900
950
1000
m/z, Da

ONTORES
BIOTECHNOLOGIES

Quality Inspection Report

**Product Name:**	PA3074
**Lot Num:**	OP1111020SF-002
**Sequence:**	CwR 小写为 D 型氨基酸
**Molecular Weight:**	462.55
**Mass Spectral Analysis:**	see attached MS spectrogram
**HPLC Analysis:**	Peptide purity：97.75 %
**Column :**	agilent ZORBAX 300SB-C18 5um 4.6*250mm
**Mobile phase:**	A:0.1%TFA in 100%H2O<br>B:ACN
**Appearance:**	white lyophilized powder
**Counter Ion:**	Trifluoroacetate
**Date of Mfg:**	11/20/20

浙江昂拓莱司生物技术有限公司 质检专用章

Quality Assurance By: ____________________
(Quality Control Department)

Date: 11/20/20

Zhejiang Ontores Biotechnologies Co.,Ltd

Address: NO.16 Road of Long Tan Hangzhou,Zhejiang , China, 311121
Email:support@ontores.com Fax: 0571-88863303 Tel: 0571-88607602 http://www.ontoresinc.com

Empower 3 SOFTWARE

Qc Reprot

SAMPLE INFORMATION

Sample Name:	3074 234	Acquired By:	System
Sample Type:	Unknown	Sample Set Name	QC20201119A
Vial:	85	Acq. Method Set:	10%_30% ACN in 20min in CD
Injection #:	1	Processing Method	Agilent SB300 250mm 5um
Injection Volume:	20.00 ul	Channel Name:	220.0nm
Run Time:	20.0 Minutes	Proc. Chnl. Descr.:	PDA 220.0 nm
Date Acquired:	11/19/2020 17:04:30 PM CST		
Date Processed:	11/20/2020 9:14:43 AM CST		

Auto-Scaled Chromatogram

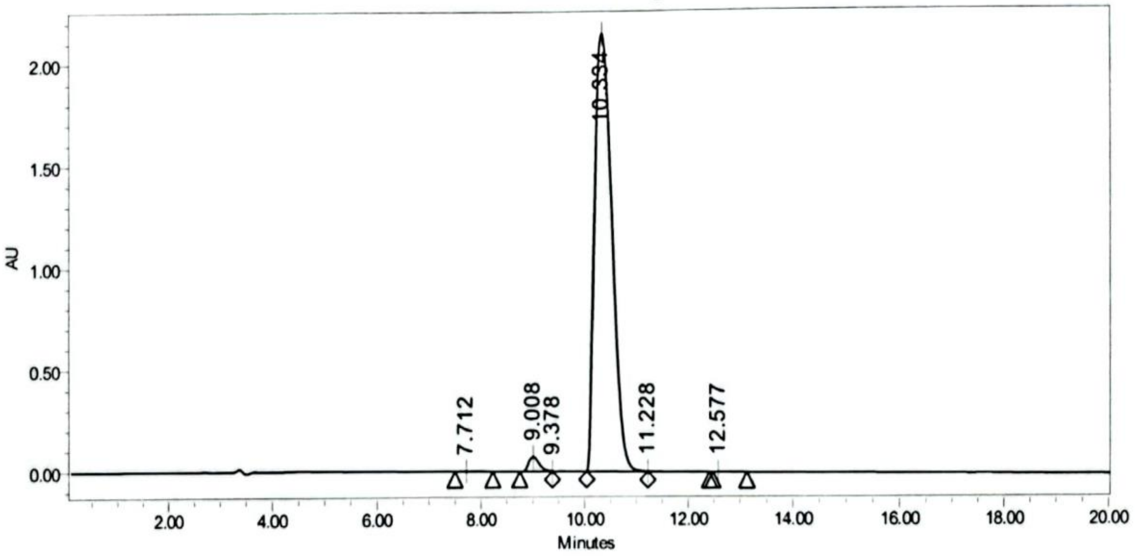

Peak Results

	Name	RT	Area	Height	% Area	Amount	Units
1		7.712	13133	604	0.03		
2		9.008	1026444	71274	2.00		
3		9.378	40481	2284	0.08		
4		10.334	50077617	2152938	97.75		
5		11.22ε	62301	2479	0.12		
6		12.577	7855	451	0.02		

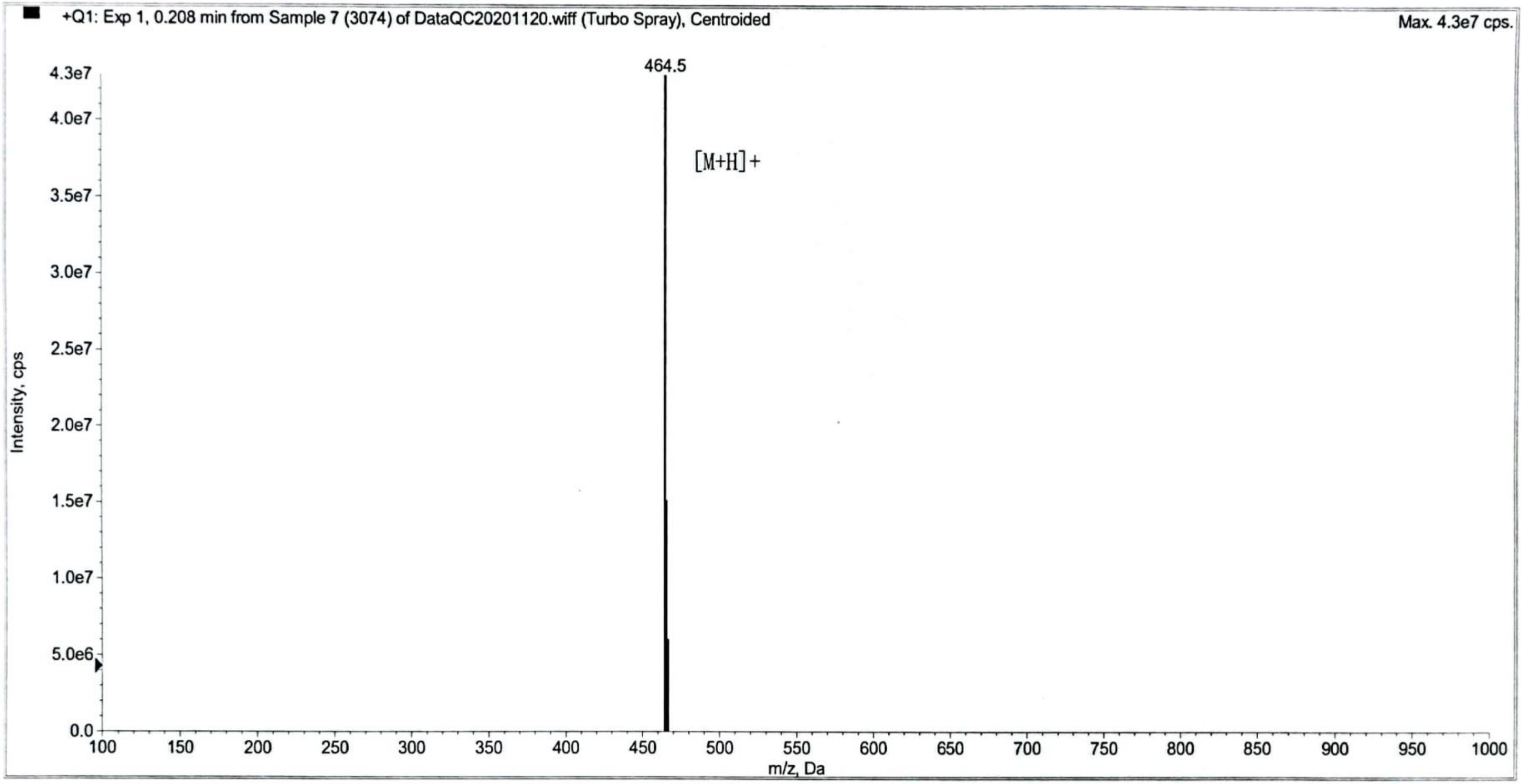
+Q1: Exp 1, 0.208 min from Sample 7 (3074) of DataQC20201120.wiff (Turbo Spray), Centroided
Max. 4.3e7 cps.
464.5
[M+H]+
Intensity, cps
4.3e7
4.0e7
3.5e7
3.0e7
2.5e7
2.0e7
1.5e7
1.0e7
5.0e6
0.0
100
150
200
250
300
350
400
450
500
550
600
650
700
750
800
850
900
950
1000
m/z, Da

ONTORES
BIOTECHNOLOGIES

Quality Inspection Report

**Product Name:**	PA3075
**Lot Num:**	OP1111020SF-003
**Sequence:**	CWr 小写为D型氨基酸
**Molecular Weight:**	462.55
**Mass Spectral Analysis:**	see attached MS spectrogram
**HPLC Analysis:**	Peptide purity：98.98%
**Column :**	agilent ZORBAX 300SB-C18 5um 4.6*250mm
**Mobile phase:**	A:0.1%TFA in 100%H2O<br>B:ACN
**Appearance:**	white lyophilized powder
**Counter Ion:**	Trifluoroacetate
**Date of Mfg:**	11/20/20

浙江昂拓莱司生物技术有限公司 质检专用章

Quality Assurance By: ____________________
(Quality Control Department)

Date: 11/20/20

Zhejiang Ontores Biotechnologies Co.,Ltd

Address: NO.16 Road of Long Tan Hangzhou,Zhejiang , China, 311121
Email:support@ontores.com **Fax:** 0571-88863303 **Tel:** 0571-88607602 http://www.ontoresinc.com

Empower 2 software

Qc Report

SAMPLE INFORMATION

Sample Name:	3075-36	Acquired By:	System
Sample Type:	Unknown	Sample Set Name:	QC20201119B
Vial:	34	Acq. Method Set:	7%_27% ACN 20min AB
Injection #:	1	Processing Method:	Agilent SB300 5um 250mm
Injection Volume:	20.00 ul	Channel Name:	220.0nm
Run Time:	20.0 Minutes	Proc. Chnl. Descr.:	PDA 220.0 nm
Date Acquired:	11/19/2020 6:34:46 PM CST		
Date Processed:	11/20/2020 9:45:10 AM CST		

Auto-Scaled Chromatogram

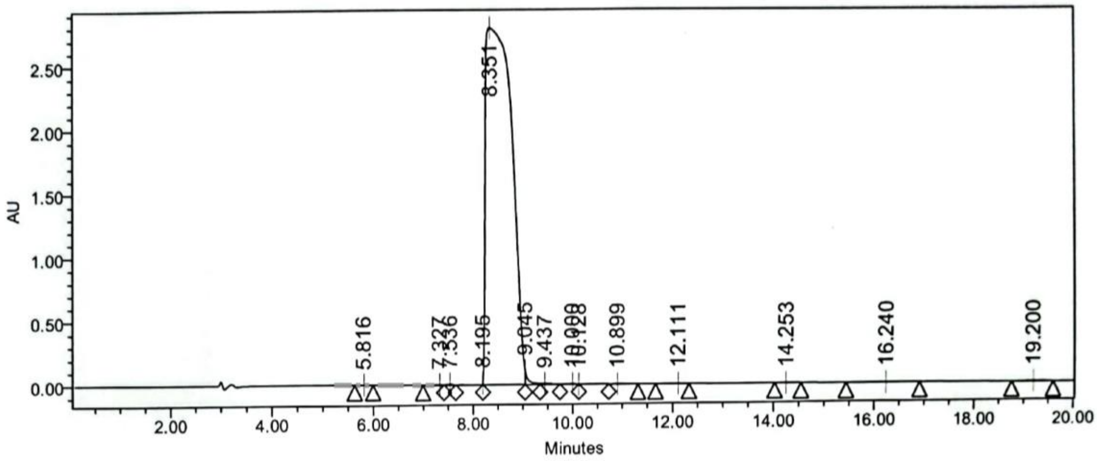

Peak Results

	Name	RT	Area	Height	% Area	Amount	Units
1		5.816	10237	1656	0.01		
2		7.327	43509	4118	0.04		
3		7.536	67363	7585	0.07		
4		8.195	37027	12012	0.04		
5		8.351	100826346	2797308	98.98		
6		9.045	464182	108856	0.46		
7		9.437	128234	7949	0.13		
8		10.000	68530	5694	0.07		
9		10.128	31873	1417	0.03		
10		10.899	20624	1230	0.02		
11		12.111	25322	3062	0.02		

Peak Results

	Name	RT	Area	Height	% Area	Amount	Units
12		14.253	14845	1054	0.01		
13		16.240	74862	1448	0.07		
14		19.200	57377	2587	0.06		

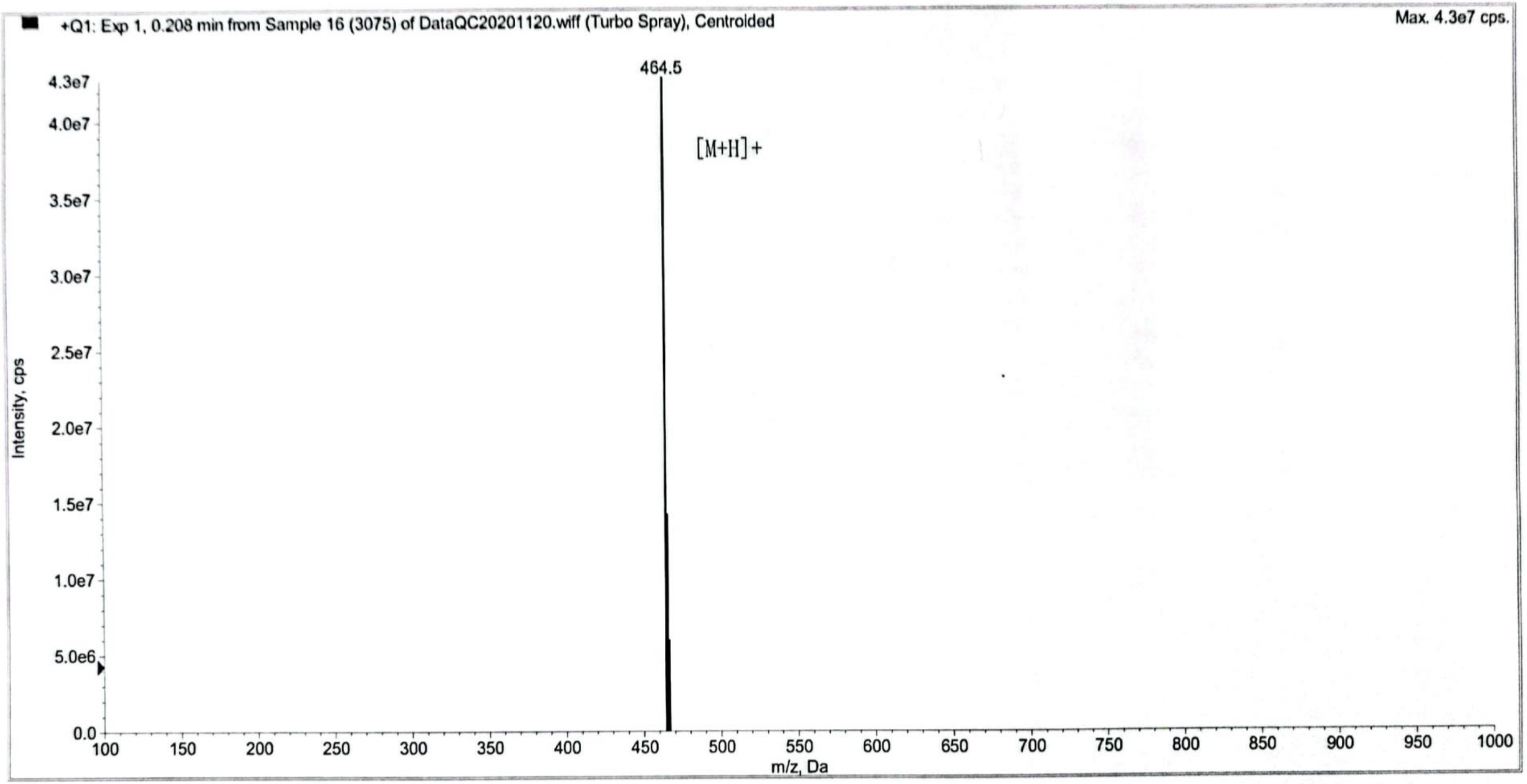

+Q1: Exp 1, 0.208 min from Sample 16 (3075) of DataQC20201120.wiff (Turbo Spray), Centroided
Max. 4.3e7 cps.
464.5
[M+H]+
Intensity, cps
4.3e7
4.0e7
3.5e7
3.0e7
2.5e7
2.0e7
1.5e7
1.0e7
5.0e6
0.0
100
150
200
250
300
350
400
450
500
550
600
650
700
750
800
850
900
950
1000
m/z, Da

ONTORES
BIOTECHNOLOGIES

Quality Inspection Report

**Product Name:**	PA3076
**Lot Num:**	OP1111020SF-004
**Sequence:**	cwR 小写为 D 型氨基酸
**Molecular Weight:**	482.55
**Mass Spectral Analysis:**	see attached MS spectrogram
**HPLC Analysis:**	Peptide purity：99.39 %
**Column :**	agilent ZORBAX 300SB-C18 5um 4.6*250mm
**Mobile phase:**	A:0.1%TFA in 100%H2O<br>B:ACN
**Appearance:**	white lyophilized powder
**Counter Ion:**	Trifluoroacetate
**Date of Mfg:**	11/23/20

Quality Assurance By: ______________________
(Quality Control Department)

浙江昂拓莱司生物技术有限公司
质检专用章

Date: 11/23/20

Zhejiang Ontores Biotechnologies Co.,Ltd

Address: NO.16 Road of Long Tan Hangzhou,Zhejiang , China, 311121
Email:support@ontores.com Fax: 0571-88863303 Tel: 0571-88607802 http://www.ontoresinc.com

Empower 3

Qc Reprot

SAMPLE INFORMATION

Sample Name:	3076-37	Acquired By:	System
Sample Type:	Unknown	Sample Set Name	QC20201119A
Vial:	31	Acq. Method Set:	6%_26% ACN in 20min in CD
Injection #:	1	Processing Method	Agilent SB300 250mm 5um
Injection Volume:	10.00 ul	Channel Name:	220.0nm
Run Time:	20.0 Minutes	Proc. Chnl. Descr.:	PDA 220.0 nm
Date Acquired:	11/19/2020 19:25:36 PM CST		
Date Processed:	11/23/2020 9:01:45 AM CST		

Auto-Scaled Chromatogram

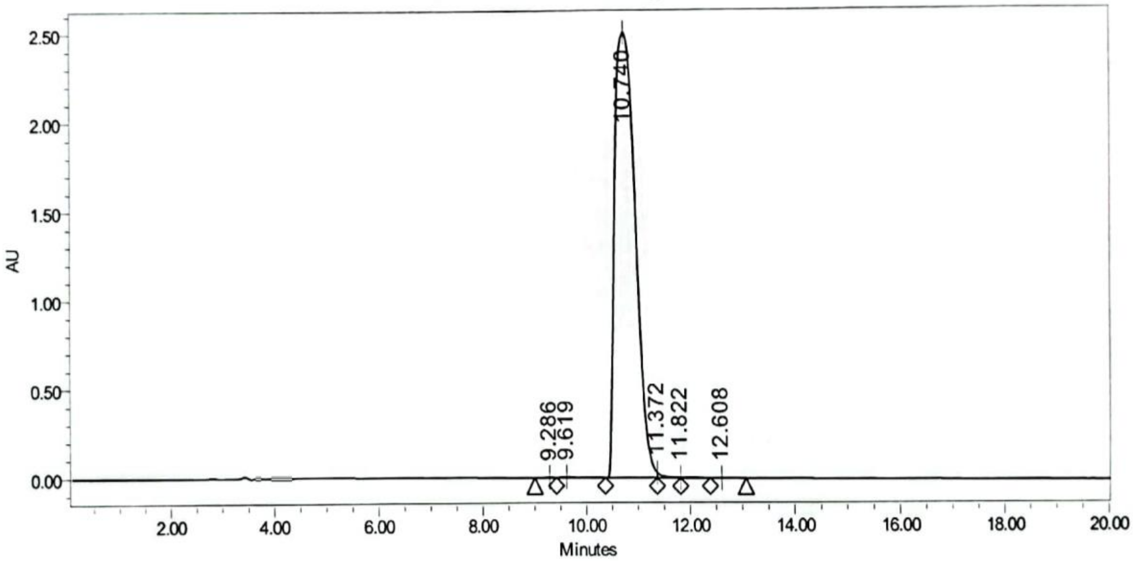

Peak Results

	Name	RT	Area	Height	% Area	Amount	Units
1		9.286	20273	1555	0.03		
2		9.619	98417	5275	0.14		
3		10.740	69035742	2507946	99.39		
4		11.372	228171	29116	0.33		
5		11.822	56239	2349	0.08		
6		12.608	18785	678	0.03		

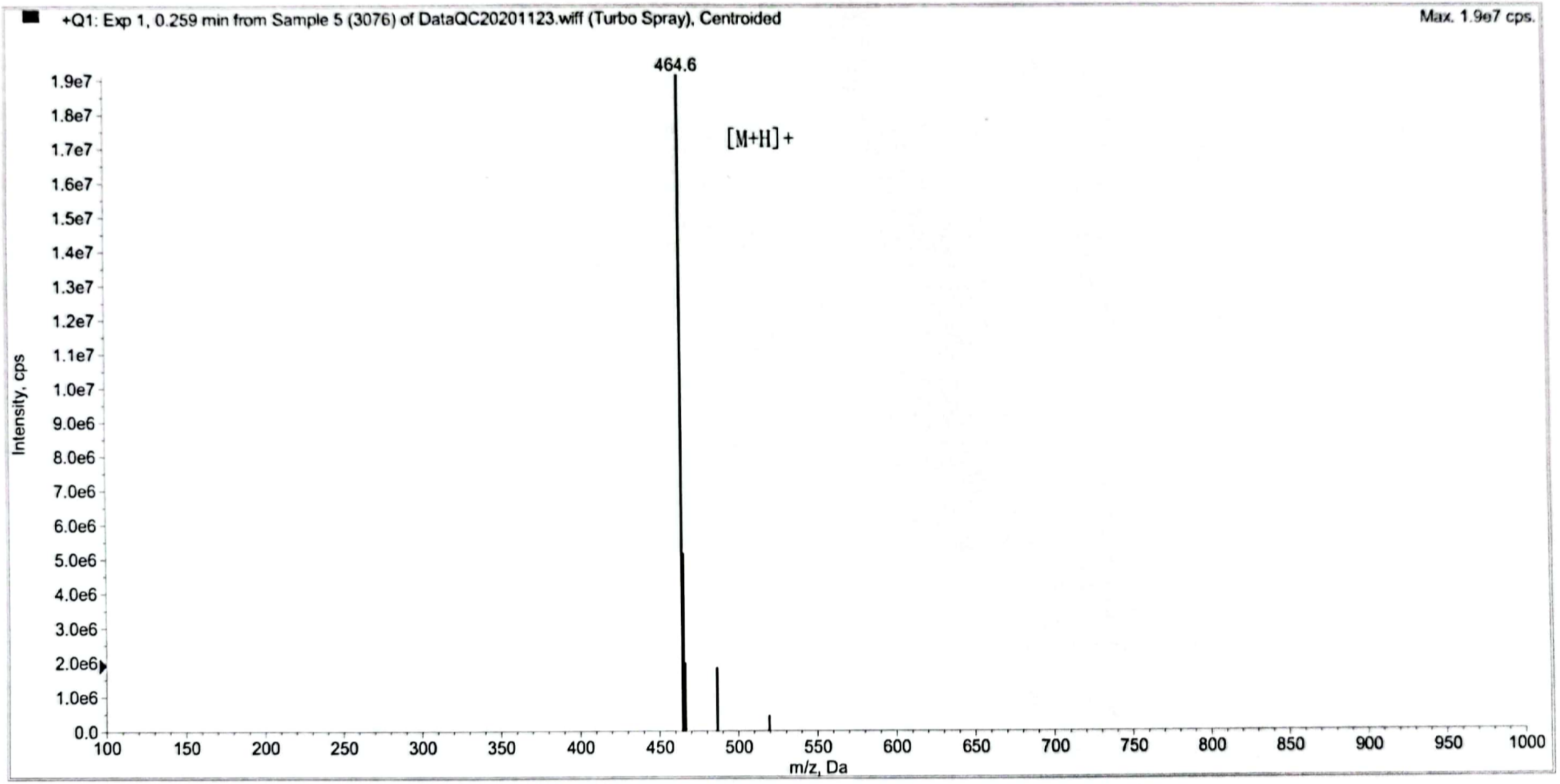
+Q1: Exp 1, 0.259 min from Sample 5 (3076) of DataQC20201123.wiff (Turbo Spray), Centroided
Max. 1.9e7 cps.
464.6
[M+H]+
Intensity, cps
1.9e7
1.8e7
1.7e7
1.6e7
1.5e7
1.4e7
1.3e7
1.2e7
1.1e7
1.0e7
9.0e6
8.0e6
7.0e6
6.0e6
5.0e6
4.0e6
3.0e6
2.0e6
1.0e6
0.0
100
150
200
250
300
350
400
450
500
550
600
650
700
750
800
850
900
950
1000
m/z, Da

ONTORES
BIOTECHNOLOGIES

Quality Inspection Report

**Product Name:**	PA3077
**Lot Num:**	OP1111020SF-005
**Sequence:**	cWr 小写为 D 型氨基酸
**Molecular Weight:**	462.55
**Mass Spectral Analysis:**	see attached MS spectrogram
**HPLC Analysis:**	Peptide purity：97.47%
**Column :**	agilent ZORBAX 300SB-C18 5um 4.6*250mm
**Mobile phase:**	A:0.1%TFA in 100%H2O<br>B:ACN
**Appearance:**	white lyophilized powder
**Counter Ion:**	Trifluoroacetate
**Date of Mfg:**	11/20/20

浙江昂拓莱司生物技术有限公司 质检专用章

Quality Assurance By: ______________________
(Quality Control Department)

Date: 11/20/20

Zhejiang Ontores Biotechnologies Co.,Ltd

Address: NO.16 Road of Long Tan Hangzhou,Zhejiang , China, 311121
Email:support@ontores.com Fax: 0571-88863303 Tel: 0571-88607602 http://www.ontoresinc.com

Empower 2 software

Qc Report

SAMPLE INFORMATION

Sample Name:	3077-46	Acquired By:	System
Sample Type:	Unknown	Sample Set Name:	QC20201119B
Vial:	87	Acq. Method Set:	10%_30% ACN 20min AB
Injection #:	1	Processing Method:	Agilent SB300 5um 250mm
Injection Volume:	20.00 ul	Channel Name:	220.0nm
Run Time:	20.0 Minutes	Proc. Chnl. Descr.:	PDA 220.0 nm
Date Acquired:	11/19/2020 4:44:47 PM CST		
Date Processed:	11/20/2020 9:44:15 AM CST		

Auto-Scaled Chromatogram

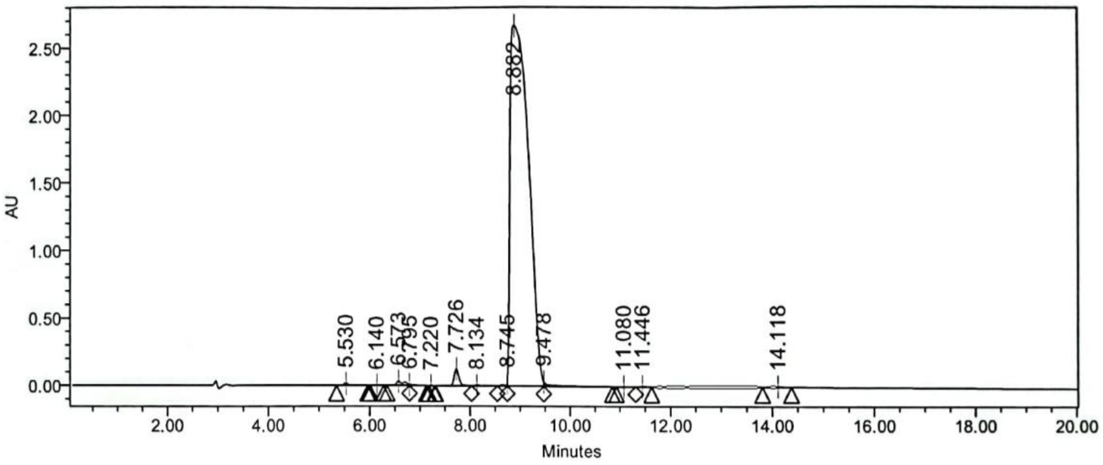

Peak Results

	Name	RT	Area	Height	% Area	Amount	Units
1		5.530	110957	14559	0.16		
2		6.140	7327	914	0.01		
3		6.573	358144	31736	0.51		
4		6.795	40305	7198	0.06		
5		7.220	1106	268	0.00		
6		7.726	820997	126034	1.17		
7		8.134	40457	3482	0.06		
8		8.745	55218	21807	0.08		
9		8.882	68185959	2672518	97.47		
10		9.478	263195	32841	0.38		
11		11.080	7427	569	0.01		

Peak Results

	Name	RT	Area	Height	% Area	Amount	Units
12		11.446	23860	3084	0.03		
13		14.118	39913	4755	0.06		

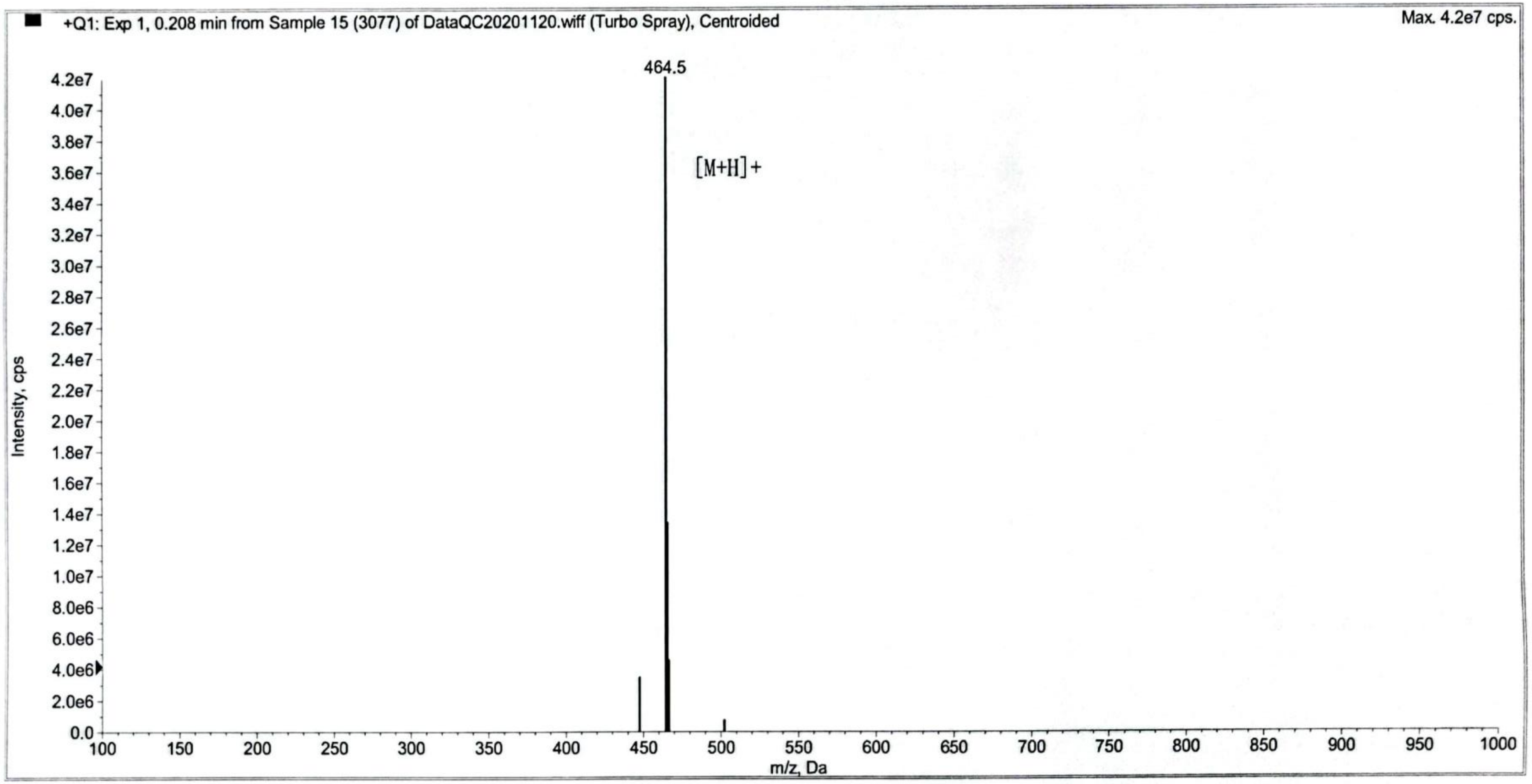

+Q1: Exp 1, 0.208 min from Sample 15 (3077) of DataQC20201120.wiff (Turbo Spray), Centroided
Max. 4.2e7 cps.
464.5
[M+H]+
Intensity, cps
4.2e7
4.0e7
3.8e7
3.6e7
3.4e7
3.2e7
3.0e7
2.8e7
2.6e7
2.4e7
2.2e7
2.0e7
1.8e7
1.6e7
1.4e7
1.2e7
1.0e7
8.0e6
6.0e6
4.0e6
2.0e6
0.0
100
150
200
250
300
350
400
450
500
550
600
650
700
750
800
850
900
950
1000
m/z, Da

ONTORES
BIOTECHNOLOGIES

Quality Inspection Report

**Product Name:**	PA3073
**Lot Num:**	OP1111020SF-001
**Sequence:**	cWR 小写为 D 型氨基酸
**Molecular Weight:**	462.55
**Mass Spectral Analysis:**	see attached MS spectrogram
**HPLC Analysis:**	Peptide purity：97.39%
**Column :**	agilent ZORBAX 300SB-C18 5um 4.6*250mm
**Mobile phase:**	A:0.1%TFA in 100%H2O<br>B:ACN
**Appearance:**	white lyophilized powder
**Counter Ion:**	Trifluoroacetate
**Date of Mfg:**	11/20/20

浙江昂拓莱司生物技术有限公司 质检专用章

Quality Assurance By: ____________________
(Quality Control Department)

Date: 11/20/20

Zhejiang Ontores Biotechnologies Co.,Ltd

Address: NO.16 Road of Long Tan Hangzhou,Zhejiang , China, 311121
Email:support@ontores.com Fax: 0571-88863303 Tel: 0571-88607602 http://www.ontoresinc.com

Empower 3

Qc Reprot

SAMPLE INFORMATION

Sample Name:	3073-57	Acquired By:	System
Sample Type:	Unknown	Sample Set Name	QC20201119A
Vial:	94	Acq. Method Set:	10%_30% ACN in 20min in CD
Injection #:	1	Processing Method	Agilent SB300 250mm 5um
Injection Volume:	20.00 ul	Channel Name:	220.0nm
Run Time:	20.0 Minutes	Proc. Chnl. Descr.:	PDA 220.0 nm
Date Acquired:	11/19/2020 15:41:20 PM CST		
Date Processed:	11/20/2020 9:44:52 AM CST		

Auto-Scaled Chromatogram

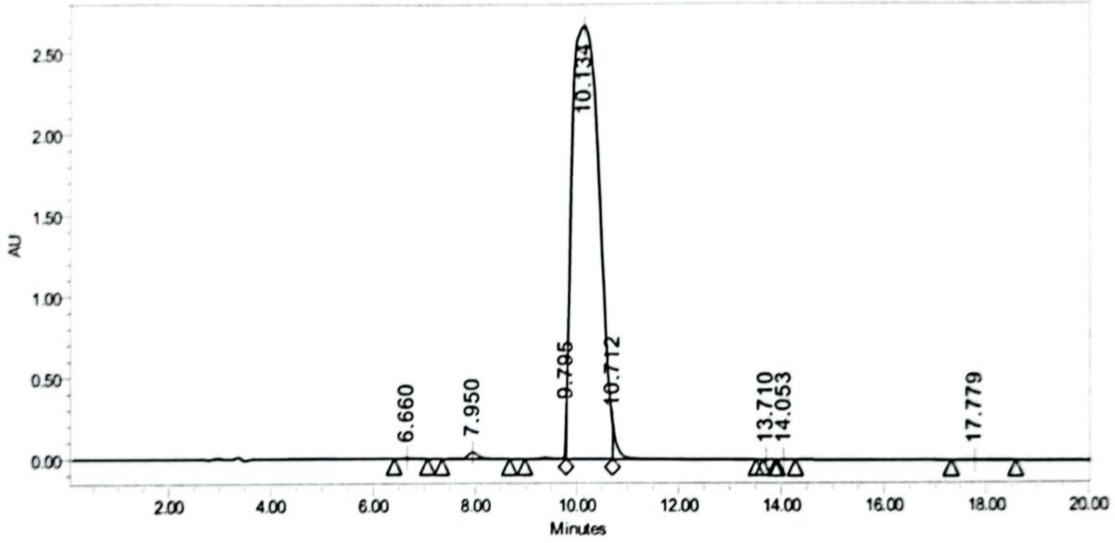

Peak Results

	Name	RT	Area	Height	% Area	Amount	Units
1		6.660	63809	4464	0.06		
2		7.950	619446	38431	0.61		
3		9.795	440753	272450	0.43		
4		10.134	99269428	2672349	97.39		
5		10.712	1483654	221948	1.46		
6		13.710	1734	169	0.00		
7		14.053	6256	597	0.01		
8		17.779	43131	996	0.04		

Tel(Shanghai):+86-21-[illegible]
Tel(Suzhou):+86-512-63930660
E-mail:inside@chinapeptides.com
outside@chinapeptides.com
Http://www.chinapeptides.com

Certificate of Analysis

**Product Name:**	04010054451
**Sequence:**	(MAA)-Gwr (Remark: MAA 是 2-甲基丙烯酸,w is D-Trp,r is D-Arg)
**Sequence(Three Letters Code):**	(MAA)-Gly-(D-Trp)-(D-Arg) (Remark: MAA 是 2-甲基丙烯酸)
**Purity:**	97.74%
**Molecular Weight:**	485.56
**Solubility:**	1mg/ml in 17%ACN/83%H_2O

Test	Specification	Result
**Purity:**	HPLC<br>(See attached RP-HPLC chromatogram)	Conforms
**MS Analysis:**	ESI-MS<br>(See attached MS spectrum)	Conforms
**Counter Ion:**	Trifluoroacetate	Conforms
**Appearance:**	Lyophilized powder or Crystallization	Conforms

Quality Assurance By: 周炎　　**Position:** Manager　　**Date:** 2020-09-16

苏州强耀生物科技有限公司 检测专用章

Important: The peptides can be used for research only. Most of the peptides are lyophilized white or faint yellow powder while fluorescent modified ones have special colors. The state of peptides with strong hydrophilic properties may be crystalline or liquid which does not affect for use. Before experiment, please choose proper solvent for your experiment to dissolve peptides. If peptides cannot be dissolved under harsh conditions, we can carry out feasibility study. Storage conditions:-20°C, seal, avoid light, dry.

Please test the sample within two weeks after receiving it.

注意：本品仅供科研，生产用途，不得直接用于人体。

Tel(Shanghai):+86-21-50795728
Tel(Suzhou):+86-512-63930660
E-mail:inside@chinapeptides.com
outside@chinapeptides.com
Http://www.chinapeptides.com

HPLC Analysis Report

Measurement:	Peak Area	Run Time:	20min
Calculation Type:	Percent	Wavelength :	220nm
Flow Rate :	1.0ml/min	Inj.Vol:	10uL
Column:	Kromasil 100-5C18,4.6mmX250mm,5 micron	Column Temp:	30℃
Buffer A :	0.1%TFA in Acetonitrile	Buffer B:	0.1%TFA in water

Gradient(linear):

	A	B
0.0min	22%	78%
20min	47%	53%
20.1min	100%	0%

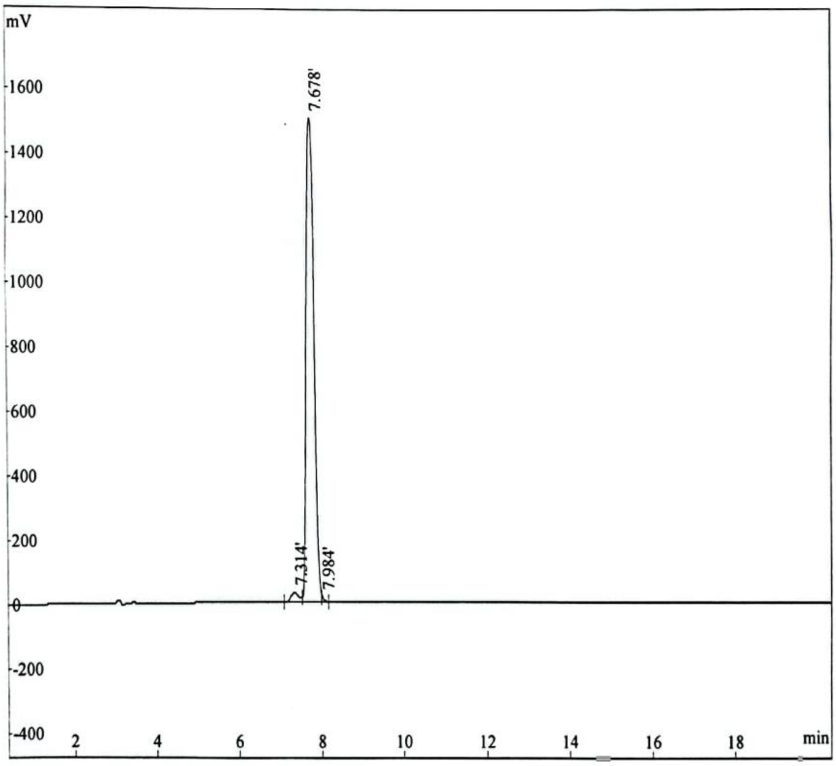

Rank	Time	Name	Conc.	Area
1	7.314		2.063	386722
2	7.678		97.74	18324982
3	7.984		0.1979	37113
Total			100	18748817

Tel(Shanghai):+86-21-50795728
Tel(Suzhou):+86-512-63930660
E-mail:inside@chinapeptides.com
outside@chinapeptides.com
Http://www.chinapeptides.com

MS Analysis Report

Ion Source: ESI	Capillary(KV):±(2500~3000)
Desolvation(L/hr):800	Desolvation Temp:450℃
Cone(V): 30~50	Run Time: 1min

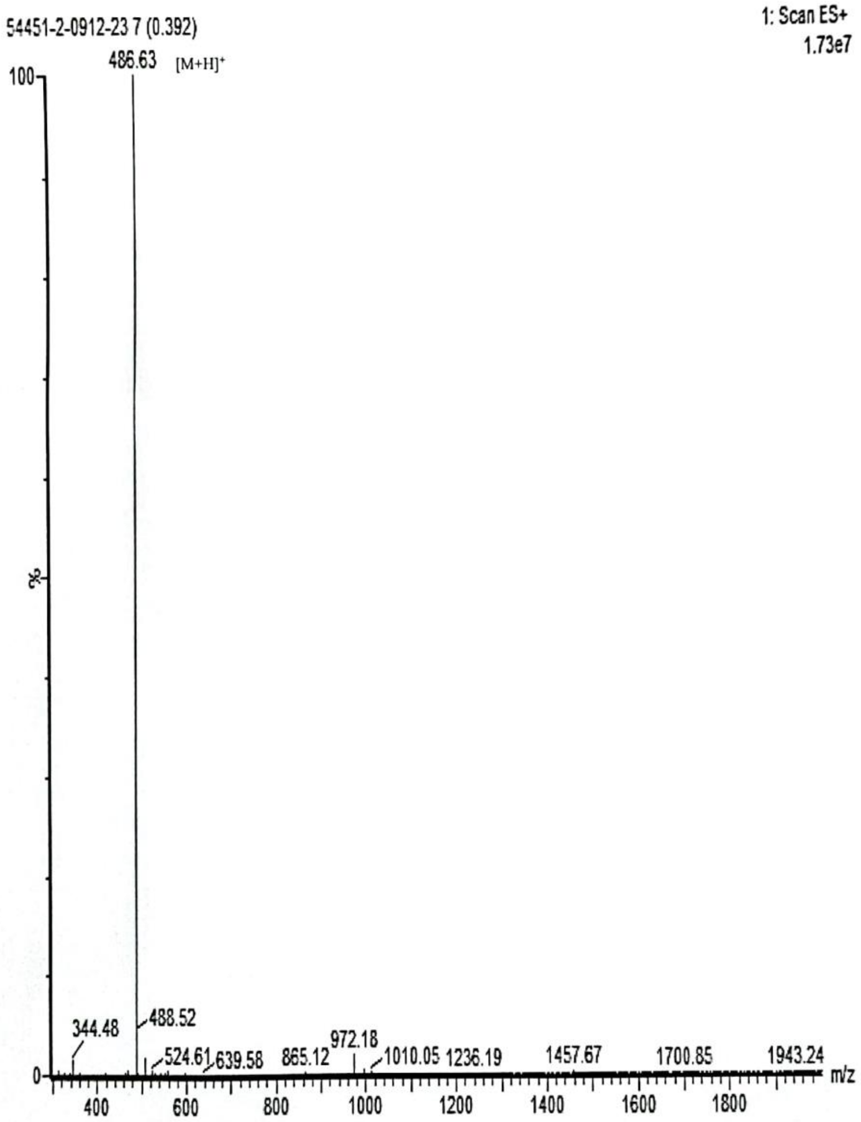

ONTORES
BIOTECHNOLOGIES

Quality Inspection Report

**Product Name:**	PA3078
**Lot Num:**	OP1111020SF-006
**Sequence:**	Cwr 小写为 D 型氨基酸
**Molecular Weight:**	462.55
**Mass Spectral Analysis:**	see attached MS spectrogram
**HPLC Analysis:**	Peptide purity：99.26%
**Column :**	agilent ZORBAX 300SB-C18 5um 4.6*250mm
**Mobile phase:**	A:0.1%TFA in 100%H2O<br>B:ACN
**Appearance:**	white lyophilized powder
**Counter Ion:**	Trifluoroacetate
**Date of Mfg:**	11/20/20

浙江昂拓莱司生物技术有限公司
质检专用章

Quality Assurance By: ____________________
(Quality Control Department)

Date: 11/20/20

Zhejiang Ontores Biotechnologies Co.,Ltd

Address: NO.16 Road of Long Tan Hangzhou,Zhejiang , China, 311121
Email:support@ontores.com Fax: 0571-88863303 Tel: 0571-88607602 http://www.ontoresinc.com

Empower 3 SOFTWARE

Qc Reprot

SAMPLE INFORMATION

Sample Name:	3078	Acquired By:	System
Sample Type:	Unknown	Sample Set Name	QC20201119A
Vial:	93	Acq. Method Set:	9%_29% ACN in 20min in CD
Injection #:	1	Processing Method	Agilent SB300 250mm 5um
Injection Volume:	20.00 ul	Channel Name:	220.0nm
Run Time:	20.0 Minutes	Proc. Chnl. Descr.:	PDA 220.0 nm
Date Acquired:	11/19/2020 16:36:47 PM CST		
Date Processed:	11/20/2020 13:39:34 PM CST		

Auto-Scaled Chromatogram

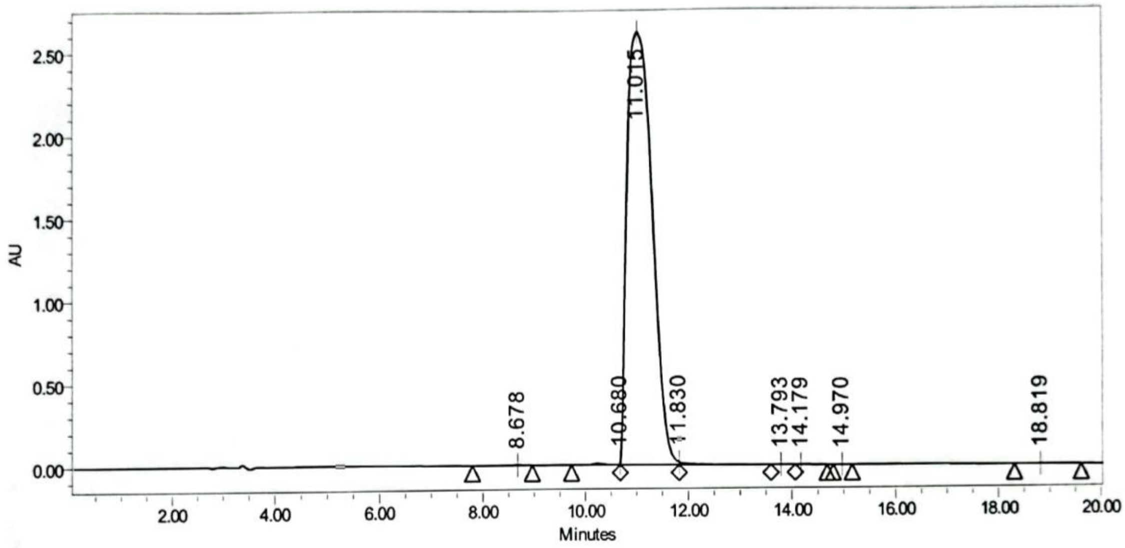

Peak Results

	Name	RT	Area	Height	% Area	Amount	Units
1		8.678	41601	2046	0.05		
2		10.680	199147	13754	0.22		
3		11.015	88968229	2625791	99.26		
4		11.830	322420	16437	0.36		
5		13.793	29408	1485	0.03		
6		14.179	12812	749	0.01		
7		14.970	7925	663	0.01		
8		18.819	47365	1267	0.05		

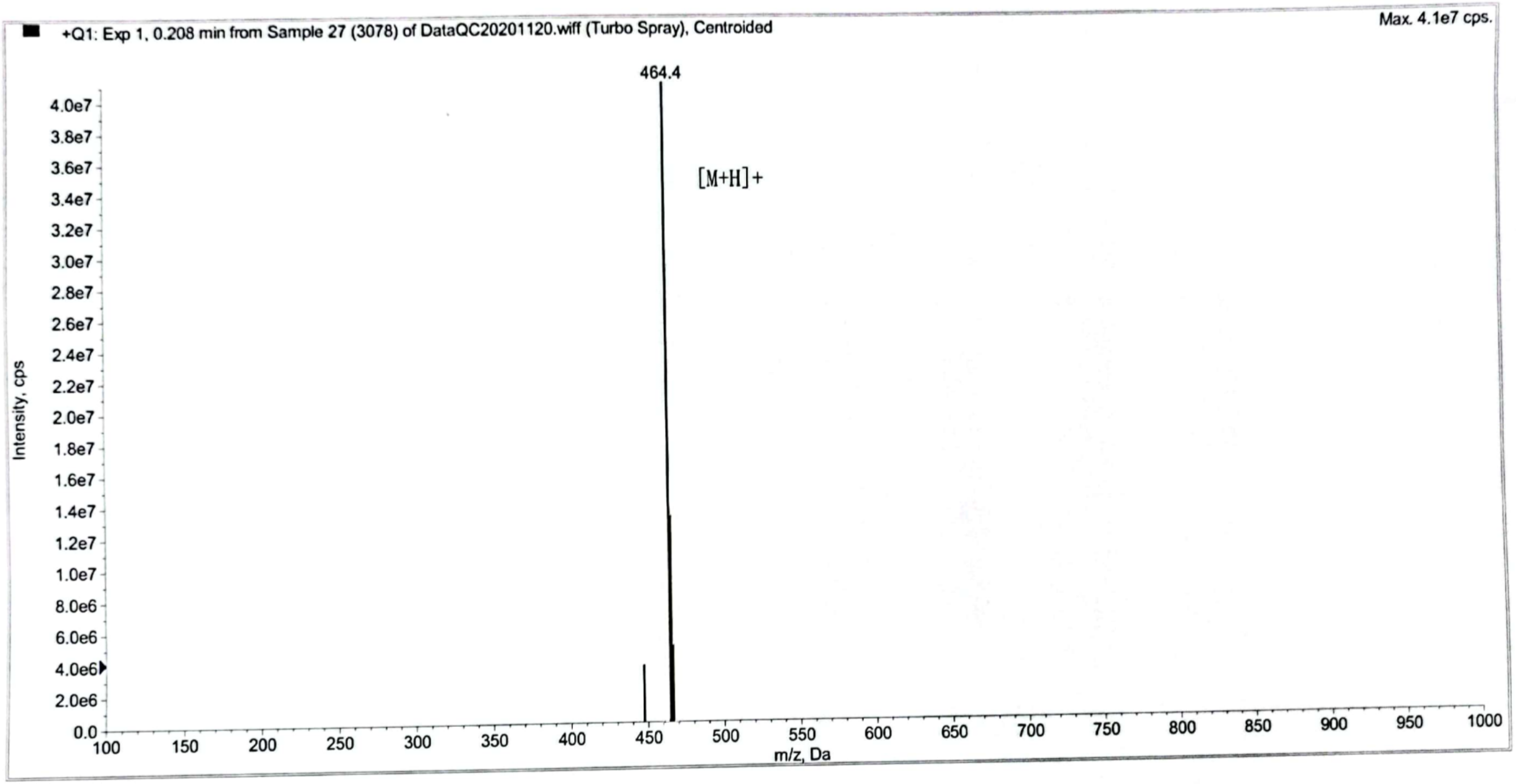
+Q1: Exp 1, 0.208 min from Sample 27 (3078) of DataQC20201120.wiff (Turbo Spray), Centroided
Max. 4.1e7 cps.
464.4
[M+H]+
Intensity, cps
4.0e7
3.8e7
3.6e7
3.4e7
3.2e7
3.0e7
2.8e7
2.6e7
2.4e7
2.2e7
2.0e7
1.8e7
1.6e7
1.4e7
1.2e7
1.0e7
8.0e6
6.0e6
4.0e6
2.0e6
0.0
100
150
200
250
300
350
400
450
500
550
600
650
700
750
800
850
900
950
1000
m/z, Da

Tel(Shanghai):+86-21-50795728
Tel(Suzhou):+86-512-63930660
E-mail:inside@chinapeptides.com
outside@chinapeptides.com
Http://www.chinapeptides.com

Certificate of Analysis

Product Name: 04010054452

Sequence: (2-Mal)-wr (Remark: 2-Mal 代表 2-马来酰亚胺基乙酸,w is D-Trp,r is D-Arg)

Sequence(Three Letters Code): (2-Mal)-(D-Trp)-(D-Arg)(Remark: 2-Mal 代表 2-马来酰亚胺基乙酸)

Purity: 96.69%

Molecular Weight: 497.53

Solubility: 1mg/ml in 17%ACN/83%H_2O

Test	Specification	Result
Purity:	HPLC<br>(See attached RP-HPLC chromatogram)	Conforms
MS Analysis:	ESI-MS<br>(See attached MS spectrum)	Conforms
Counter Ion:	Trifluoroacetate	Conforms
Appearance:	Lyophilized powder or Crystallization	Conforms

Quality Assurance By: 周炎 **Position:** Manager **Date:** 2020-09-16

[Stamp: 强耀生物科技有限公司 检测专用章]

Important: The peptides can be used for research only. Most of the peptides are lyophilized white or faint yellow powder while fluorescent modified ones have special colors. The state of peptides with strong hydrophilic properties may be crystalline or liquid which does not affect for use. Before experiment, please choose proper solvent for your experiment to dissolve peptides. If peptides cannot be dissolved under harsh conditions, we can carry out feasibility study. Storage conditions:-20°C, seal, avoid light, dry.

Please test the sample within two weeks after receiving it.

注意：本品仅供科研，生产用途，不得直接用于人体。

Tel(Shanghai):+86-21-56795728
Tel(Suzhou):+86-512-63930660
E-mail:inside@chinapeptides.com
outside@chinapeptides.com
Http://www.chinapeptides.com

HPLC Analysis Report

Measurement:	Peak Area	Run Time:	20min
Calculation Type:	Percent	Wavelength :	220nm
Flow Rate :	1.0ml/min	Inj.Vol:	10uL
Column:	Kromasil 100-5C18,4.6mmX250mm,5 micron	Column Temp:	30℃
Buffer A :	0.1%TFA in Acetonitrile	Buffer B:	0.1%TFA in water

Gradient(linear):

	A	B
0.0min	22%	78%
20min	47%	53%
20.1min	100%	0%

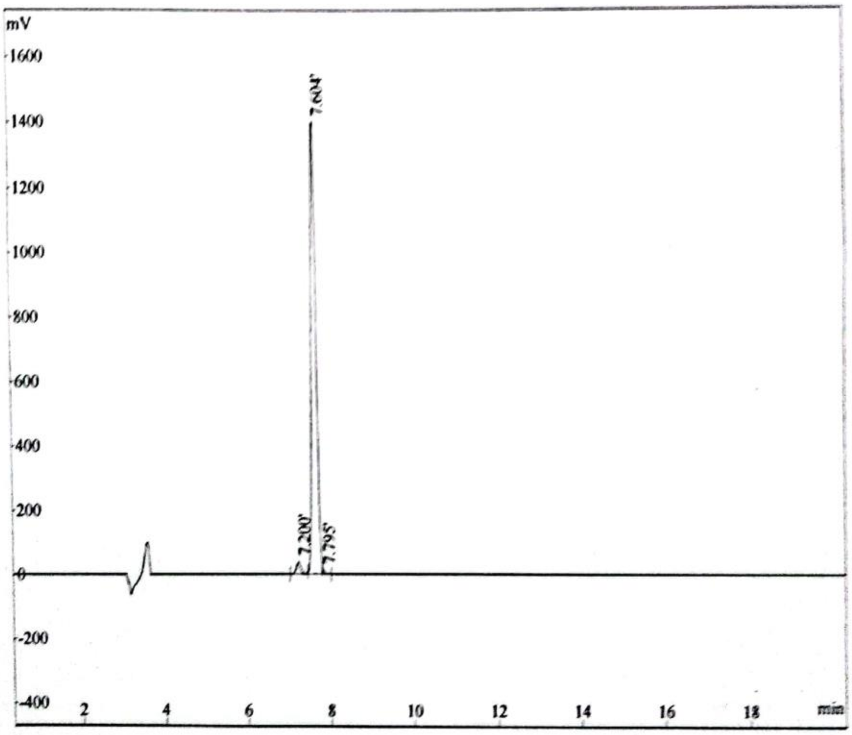

Rank	Time	Name	Conc.	Area
1	7.200		2.801	296243
2	7.604		96.69	10226035
3	7.795		0.5054	53450
Total			100	10575728

Tel(Shanghai):+86-21-50795728
Tel(Suzhou):+86-512-63930660
E-mail:inside@chinapeptides.com
outside@chinapeptides.com
Http://www.chinapeptides.com

MS Analysis Report

Ion Source: ESI   Capillary(KV):±(2500~3000)

Desolvation(L/hr):800   Desolvation Temp:450℃

Cone(V): 30~50   Run Time: 1min

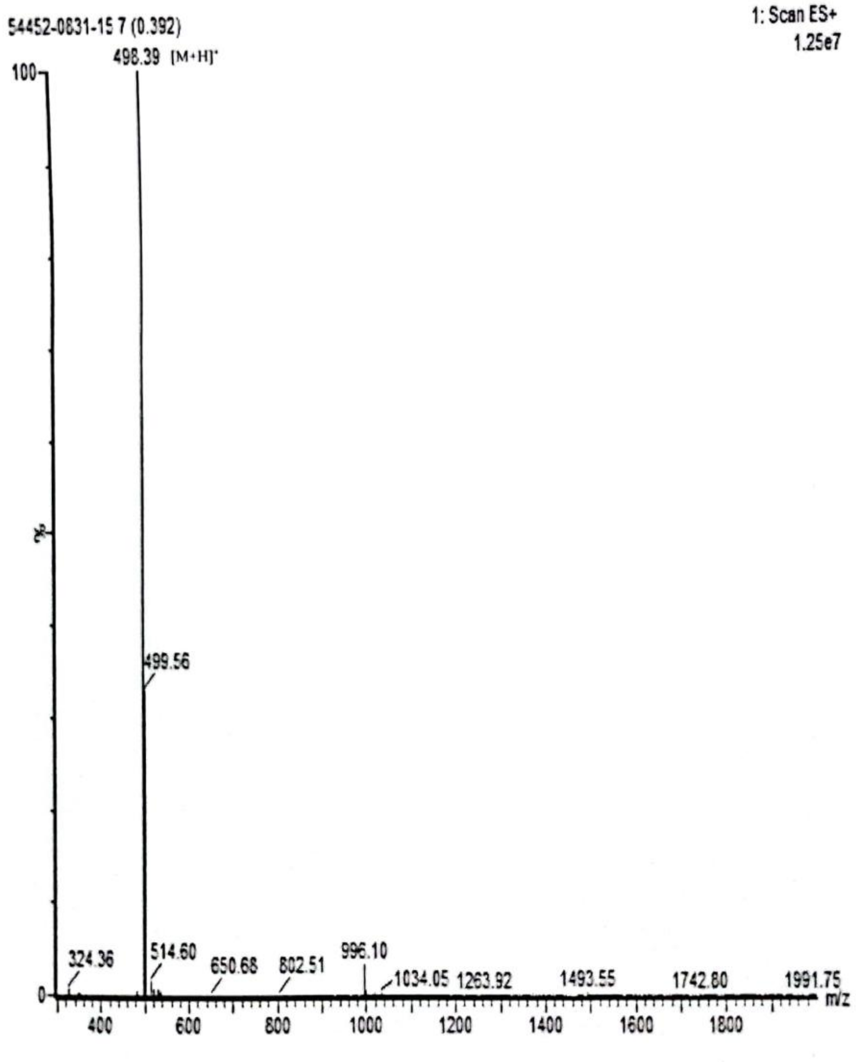

CERTIFICATE OF ANALYSIS

Product Name	N-C
Order ID	C543RGK220_1
Lot No.	C543RGK220-1/PE1654
Sequence	{MPA}WRE
Modification	N/A
Length	4AA
Storage	-20°C
Recommended Solvent*	Dimethyl sulfoxide(Analytical grade)
Comments	TFA salt

Test Items	Specifications	Results
Molecular Weight	Theoretical MW: 577.66	Consistent
HPLC purity	≥95.0%	95.9%
Appearance	White lyophilized powder	Conforms
Gross Weight	9 mg	3*3.0mg

*Note: Above recommended solvents for reference only. If there is any request for detailed dissolution conditions,we suggest you choose our 'Peptide Solubility Test Service'.

Caution:
For laboratory or further manufacturing use only. Not intended for household use. If you have any questions about the Certificate of Analysis, please contact our customer service representative at 1-877-436-7274 (Toll-Free), or 1-732-885-9188.

Certified by: Sinting Wang Date:12/15/2021

Thank you for your patronage to our Peptide services! To maintain this working relationship, we shall be grateful if you can add our webpage URL into your lab website. As a token of appreciation, you will be rewarded by 1,000 EZcoupon™ points. For more information, please contact us by e-mail at web@genscript.com

Sample Name :N-C
Sample ID :C543RGK220-1
Time Processed :3:24:27 PM
Month-Day-Year Processed :12/14/2021

Pump A : 0.065% trifluoroacetic in 100% water (v/v)
Pump B : 0.05% trifluoroacetic in 100% acetonitrile (v/v)
Total Flow:1 ml/min
Wavelength:220 nm
<<LC Time Program>>

Time	Module	Command	Value
0.01	Pumps	B.Conc	5
25.00	Pumps	B.Conc	65
25.01	Pumps	B.Conc	95
27.00	Pumps	B.Conc	95
27.01	Pumps	B.Conc	5
35.00	Pumps	B.Conc	5
35.01	Controller	Stop	

<<Column Performance>>
<Detector A>
Column :Inertsil ODS-SP 4.6 x 250 mm
Equipment: ZJ21010376

<Chromatogram>

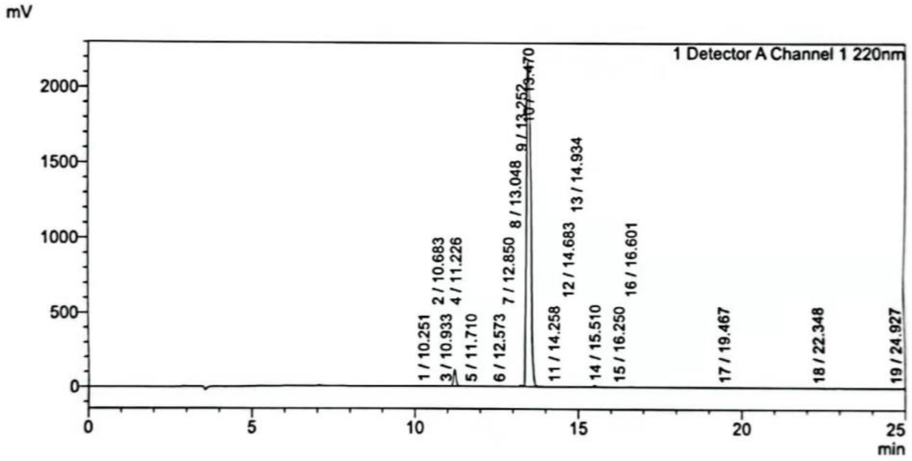

<Peak Table>

Detector A Channel 1 220nm

Peak#	Ret. Time	Area	Height	Area%
1	10.251	9694	832	0.046
2	10.683	1277	165	0.006
3	10.933	3167	266	0.015
4	11.226	606641	108704	2.909
5	11.710	4300	556	0.021
6	12.573	28558	1156	0.137
7	12.850	3788	652	0.018
8	13.048	1510	270	0.007
9	13.252	58740	9651	0.282
10	13.470	19996717	2163687	95.890
11	14.258	23229	1019	0.111
12	14.683	3679	588	0.018
13	14.934	12063	1673	0.058
14	15.510	73856	9906	0.354
15	16.250	4178	239	0.020
16	16.601	8272	787	0.040

Peak#	Ret. Time	Area	Height	Area%
17	19.467	5369	421	0.026
18	22.348	5880	483	0.028
19	24.927	2988	515	0.014
Total		20853906	2301571	100.000

Mass Spectrum

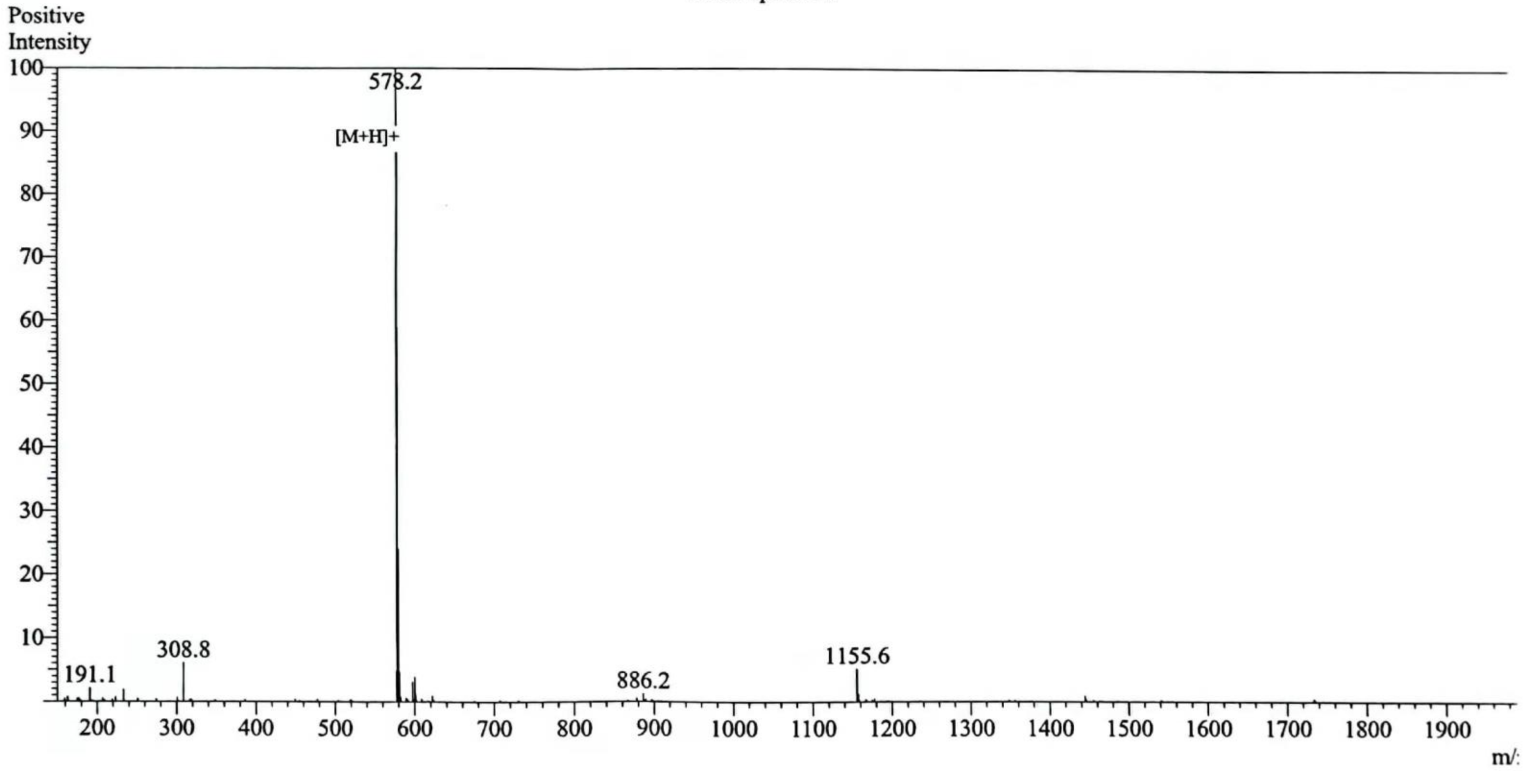

Sample Information	
Month-Day Processed :	12/14/21
Time Processed :	15:17:06
Injection Volume :	0.2
Sample Name :	N-C
Sample ID :	C543RGK220-1
Theoretical MW :	577.66
Observed MW :	577.2

Interface	:ESI
Nebulizing Gas Flow	:1.5L/min
CDL Temp	:250
Block Temp	:200

Equipment	: ZJ21010035
Interface Bias	: +4.5 kV
Drying Gas Flow	:5 L/min
T.Flow	:0.2 ml/min
B.conc	:50%H2O/50%MeOH
